# The hepatokine fetuin-A disrupts functional maturation of pancreatic beta cells

**DOI:** 10.1007/s00125-021-05435-1

**Published:** 2021-03-25

**Authors:** Felicia Gerst, Elisabeth Kemter, Estela Lorza-Gil, Gabriele Kaiser, Ann-Kathrin Fritz, Rita Nano, Lorenzo Piemonti, Marie Gauder, Andreas Dahl, Silvio Nadalin, Alfred Königsrainer, Falko Fend, Andreas L. Birkenfeld, Robert Wagner, Martin Heni, Norbert Stefan, Eckhard Wolf, Hans-Ulrich Häring, Susanne Ullrich

**Affiliations:** 1 Institute for Diabetes Research and Metabolic Diseases of the Helmholtz Center Munich at the Eberhard Karls University of Tuebingen (IDM), Tuebingen, Germany; 2 Internal Medicine IV, Endocrinology, Diabetology and Nephrology, University Hospital Tuebingen, Tuebingen, Germany; 3 German Center for Diabetes Research (DZD e.V.), Neuherberg, Germany; 4 Department of Molecular Animal Breeding and Biotechnology, Gene Center and Department of Veterinary Sciences, Ludwig Maximilians University, Munich, Germany; 5 Diabetes Research Institute, IRCCS San Raffaele Scientific Institute, Milan, Italy; 6 Quantitative Biology Center (QBiC) Eberhard Karls University of Tuebingen, Tuebingen, Germany; 7 Biotechnology Center TU Dresden, Dresden, Germany; 8 Department of General, Visceral and Transplant Surgery, University Hospital Tuebingen, Tuebingen, Germany; 9 Department of General Pathology and Pathological Anatomy, University Hospital Tuebingen, Tuebingen, Germany

**Keywords:** Adaptive proliferation, Fetuin-A, FOXM1, Functional maturity, Pancreatic beta cells, TGFBR–SMAD2/3

## Abstract

**Aims/hypothesis:**

Neonatal beta cells carry out a programme of postnatal functional maturation to achieve full glucose responsiveness. A partial loss of the mature phenotype of adult beta cells may contribute to a reduction of functional beta cell mass and accelerate the onset of type 2 diabetes. We previously found that fetuin-A, a hepatokine increasingly secreted by the fatty liver and a determinant of type 2 diabetes, inhibits glucose-stimulated insulin secretion (GSIS) of human islets. Since fetuin-A is a ubiquitous fetal glycoprotein that declines peripartum, we examined here whether fetuin-A interferes with the functional maturity of beta cells.

**Methods:**

The effects of fetuin-A were assessed during in vitro maturation of porcine neonatal islet cell clusters (NICCs) and in adult human islets. Expression alterations were examined via microarray, RNA sequencing and reverse transcription quantitative real-time PCR (qRT-PCR), proteins were analysed by western blotting and immunostaining, and insulin secretion was quantified in static incubations.

**Results:**

NICC maturation was accompanied by the gain of glucose-responsive insulin secretion (twofold stimulation), backed up by mRNA upregulation of genes governing beta cell identity and function, such as *NEUROD1*, *UCN3*, *ABCC8* and *CASR* (Log_2_ fold change [Log_2_FC] > 1.6). An active TGFβ receptor (TGFBR)–SMAD2/3 pathway facilitates NICC maturation, since the TGFBR inhibitor SB431542 counteracted the upregulation of aforementioned genes and de-repressed *ALDOB*, a gene disallowed in mature beta cells. In fetuin-A-treated NICCs, upregulation of beta cell markers and the onset of glucose responsiveness were suppressed. Concomitantly, SMAD2/3 phosphorylation was inhibited. Transcriptome analysis confirmed inhibitory effects of fetuin-A and SB431542 on TGFβ-1- and SMAD2/3-regulated transcription. However, contrary to SB431542 and regardless of c*MYC* upregulation, fetuin-A inhibited beta cell proliferation (0.27 ± 0.08% vs 1.0  ± 0.1% Ki67-positive cells in control NICCs). This effect was sustained by reduced expression (Log_2_FC ≤ −2.4) of *FOXM1*, *CENPA*, *CDK1* or *TOP2A*. In agreement, the number of insulin-positive cells was lower in fetuin-A-treated NICCs than in control NICCs (14.4 ± 1.2% and 22.3 ± 1.1%, respectively). In adult human islets fetuin-A abolished glucose responsiveness, i.e. 1.7- and 1.1-fold change over 2.8 mmol/l glucose in control- and fetuin-A-cultured islets, respectively. In addition, fetuin-A reduced SMAD2/3 phosphorylation and suppressed expression of proliferative genes. Of note, in non-diabetic humans, plasma fetuin-A was negatively correlated (*p* = 0.013) with islet beta cell area.

**Conclusions/interpretation:**

Our results suggest that the perinatal decline of fetuin-A relieves TGFBR signalling in islets, a process that facilitates functional maturation of neonatal beta cells. Functional maturity remains revocable in later life, and the occurrence of a metabolically unhealthy milieu, such as liver steatosis and elevated plasma fetuin-A, can impair both function and adaptive proliferation of beta cells.

**Data availability:**

The RNAseq datasets and computer code produced in this study are available in the Gene Expression Omnibus (GEO): GSE144950; https://www.ncbi.nlm.nih.gov/geo/query/acc.cgi?acc=GSE144950

**Graphical abstract:**

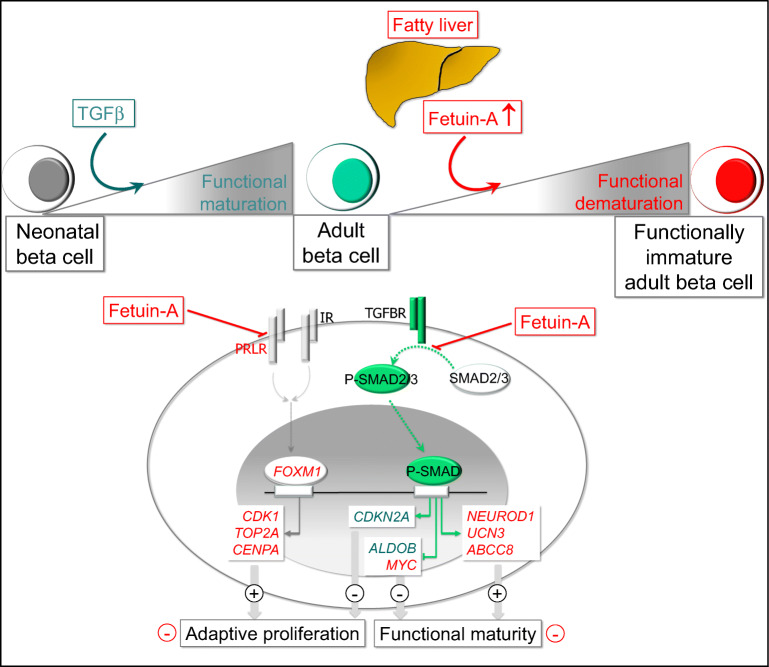

**Supplementary Information:**

The online version contains peer-reviewed but unedited supplementary material available at 10.1007/s00125-021-05435-1.

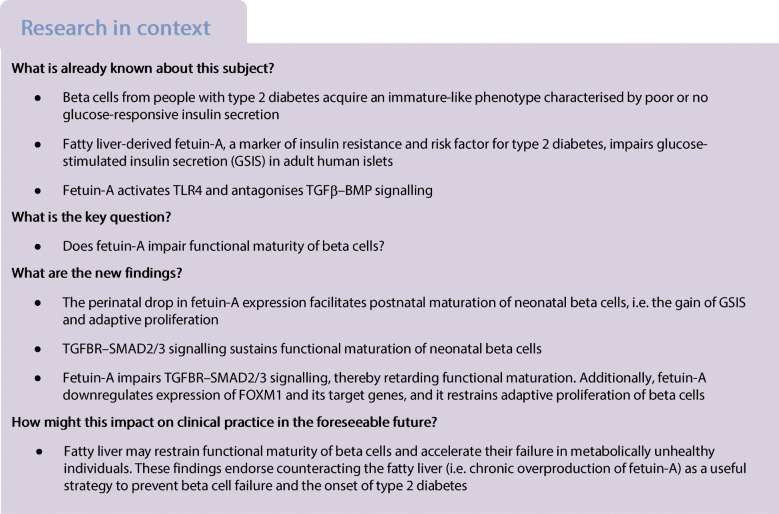



## Introduction

The functional characteristic of mature beta cells is glucose-stimulated insulin secretion (GSIS), defined as cell’s ability to secrete appropriate amounts of insulin in response to a stimulatory concentration of glucose [1, 2]. To achieve such a functional phenotype, neonatal beta cells undergo a tightly regulated process of maturation. A well-timed orchestrated upregulation of transcription factors, components of secretory machinery, proteins governing cell–cell and cell–extracellular matrix (ECM) interactions, as well as cell cycle inhibitors, are prerequisites for functional maturation [3–7]. Downregulation of genes abundantly expressed in immature beta cells complement the process [8]. The preservation of glucose responsiveness of adult beta cells is essential for healthy glucose homeostasis. However, GSIS declines with ageing or due to metabolic stress, resulting in insulin deficiency, which is the ultimate cause of diabetes onset. Growing evidence suggests that metabolic stress and insulin resistance curtail both endocrine identity and adaptive proliferation of beta cells [9].

Pancreatic endocrine specification is regulated by a plethora of signals including TGFβ–bone morphogenetic protein (BMP) [10]. In its most simple form, TGFβ–BMP signalling consists of receptor-mediated phosphorylation of SMAD transcription factors, a mandatory event for their nuclear translocation and activity. While the canonical TGFβ receptor (TGFBR) pathway relies on SMAD2/3, and BMP receptor activates SMAD1/5/8, there is crosstalk between the two signalling arms [11]. In addition, inhibitory SMAD6 and SMAD7 counteract receptor-mediated activation of SMAD transcription factors [12]. Previous work reported beneficial effects of TGFBR–SMAD signalling on beta cell function [10]. Thus, mice overexpressing SMAD7 in their islets developed diabetes, and induced pluripotent stem cell (iPSC)-derived beta cells treated with a TGFBR inhibitor prior to transplantation failed to restore normoglycaemia in recipient diabetic mice [13, 14].

Depending on cellular context, TGFBR signalling triggers either proliferation or differentiation, as the transcriptional outcome depends on post-translational modifications of SMADs, recruitment of co-factors and the epigenetic status of the chromatin [12]. In addition, inhibitory ligands compete for receptor binding [15]. Likewise, fetuin-A can bind TGFβ, thereby interfering with receptor activation [16]. Fetuin-A is a fetal glycoprotein that declines peripartum, but is increasingly secreted by hepatocytes of the fatty liver [17, 18]. Fetuin-A augments adipose tissue inflammation via activation of toll-like receptor 4 (TLR4), thereby being a major determinant of insulin resistance and type 2 diabetes [19, 20]. We previously found that fetuin-A inhibits GSIS in adult human islets [21].

Here, we investigated the mechanisms underlying the role of fetuin-A on beta cell maturation and proliferation. We used porcine neonatal islet cell clusters (NICCs), a primary cell system validated as a translational model for human islets [22]. In addition to their committed endocrine phenotype, NICCs possess intrinsic proliferative and maturation capacities, thereby being essentially different from adult beta cells, which are fully differentiated and refractory to proliferation.

## Methods

### Reagents and resources

Details of materials used are provided in the electronic supplementary material (ESM) Table 1.

### Isolation and culture of NICCs

German Landrace and Landrace-Yorkshire pigs were bred in the animal breeding facility of the Chair for Molecular Animal Breeding and Biotechnology (Munich, Germany) and NICCs were isolated as previously described [23]. Porcine pancreas (2–12-day-old piglets) was minced in Hanks’ balanced salt solution (HBSS, see ESM Methods) supplemented with 0.25% BSA, 10 mmol/l HEPES, 100 U/ml penicillin and 0.1 mg/ml streptomycin, transferred to HBSS containing 2 mg/ml collagenase V, and incubated for 120 min at 37°C. The digest was filtered and cultured in maintenance medium (see ESM Methods). At culture day (d)5–6, NICCs were placed in maturation medium supplemented with 0.6 mg/ml human serum albumin (HSA; see ESM Methods) or with human fetuin-A (0.6 mg/ml) and cultured for an additional 5 days with medium change every second day. TGFβ-1 (2 ng/ml), TLR4 inhibitor CLI-095 (5 μmol/l) or TGFBR1 inhibitor SB431542 (10 μmol/l) were added when indicated. Since FCS contains high amounts of fetuin-A, NICC maturation was conducted in FCS-free medium [24]. Domestic pig handling and NICCs isolation was approved by the veterinary authorities (district government of Bavaria) and was conducted in accordance with the German Animal Welfare Act.

### Isolated human islets

Human islets (ESM Table 2) were procured through the European Consortium for Islet Transplantation (ECIT) and cultured overnight in CMRL1066 medium (see ESM Methods). Afterwards, the islets were transferred to FCS-free medium supplemented with 0.6 mg/ml human fetuin-A or HSA (control) and cultured for an additional 2 days. CLI-095 (5 µmol/l) and TGFbeta-1 (2 ng/ml) were added during the 2 days or the last 1 h of culture, respectively. Ethics approval for the use of human islets was obtained from the Ethics Commission of the Medical Faculty of the University Hospital of Tuebingen (533/2010BO2 and 098/2017BO1) and from the ECIT centres. All experiments were performed in accordance with the abovementioned approvals, guidelines and regulations.

### Insulin secretion

NICCs and human islets were incubated in KRB containing (in mmol/l): 135 NaCl, 4.8 KCl, 1.2 Mg_2_SO_4_, 1.2 KH_2_PO_4_, 1.3 CaCl_2_, 5 NaHCO_3_, 10 HEPES (pH 7.4) and supplemented with 0.5% BSA. The islets were pre-incubated for 1 h in KRB supplemented with 2.8 mmol/l glucose followed by 1 h incubation in KRB containing glucose and test substances as indicated. Secreted insulin and insulin content were measured by ELISA or radioimmunoassay.

### Measurement of secreted TGFβ-1

At culture d5, NICCs were transferred to standard maturation medium (100 NICCs/3 ml) and cultured for an additional 5 days without medium change. On maturation days 1 (culture d6), 3 (culture d8) and 5 (culture d10) medium was collected for quantification of secreted TGFβ-1 and NICCs were lysed in RIPA buffer for protein measurement.

### Transcriptome analysis

#### RNA extraction and qRT-PCR

Total cellular RNA extracted from NICCs and human islets using a commercial kit was transcribed using random primers (Transcriptor First Strand kit, Roche, Germany). For reverse transcription quantitative real-time PCR (qRT-PCR), normalised gene expression was calculated as ratio of the cycle threshold (C_t_) values of target vs housekeeping gene (RPS13) transcripts (${2}^{-\Delta  {\mathrm{C}}_{\mathrm{t}}}$).

#### NICC transcriptome profiling with RNA sequencing

The mRNA was isolated from 0.2 μg total RNA (RNA integrity number [RIN] ≥ 9) and subjected to RNA sequencing (RNAseq) workflow as described in ESM Methods: Transcriptome analysis.

### Affymetrix microarray of adult human islets

The analysis was performed with RNA isolated from three distinct human islet preparations mixed in a ratio of 1:1:1. Therefore the differences in gene expression represent a mean of *n* = 3 independent islet preparations and all changes induced by fetuin-A are robust, donor-independent changes. Microarray analysis was performed using the Affymetrix GeneChip HG-U133 Plus2 GeneChip Array platform (Thermo Fischer Scientific, MA, USA) and primary data analysis was performed with Affymetrix software GeneChip Operating System (GCOS) v1.4.

### Human pancreatic resections

Patients (*n* = 22 non-diabetic; *n* = 32 with impaired glucose tolerance [IGT]/impaired fasting glucose [IFG] and *n* = 24 with type 2 diabetes, ESM Table 3) undergoing surgery for removal of pancreatic tumours provided written informed consent. Collection of human material and the study was approved by the Ethic Commission of Medical Faculty of the University of Tuebingen (697/2011BO1 and 355/2012BO2). Fasting blood samples were collected prior to anaesthesia and used to assess metabolic traits. Tumour-free pancreatic tissue was dissected, immediately fixed in 10% neutral buffered formalin and further processed for insulin immunostaining. Classification of normal glucose tolerance, IGT/IFG or type 2 diabetes was performed according to clinical history, fasting glucose and HbA_1c_ levels, using ADA criteria.

### Immunostaining

Isolated human islet cells (ECIT donors), whole NICCs and NICC cryosections were fixed in 4% formalin, permeabilised with ice-cold 0.2–1% Triton-X-100-PBS, blocked with 10% FCS-PBS, and incubated with primary antibodies against SMAD2/3, Ki67, p16/Ink4a or insulin (1:100 in 10% FCS-PBS), followed by incubation with anti-rabbit/anti-mouse Alexa-Fluor488/546-IgG (1:1000 in 10% FCS-PBS). Nuclei were stained with TOPRO3 or DAPI. Fluorescent imaging was performed with laser scanning confocal microscope (Leica Microsystems, Germany) or ApoTome System (Carl Zeiss Microscopy, Germany).

Tissue sections from formalin-fixed, paraffin-embedded human pancreatic resections were incubated overnight with anti-insulin antibody (1:1000) and visualised using the Opti-View DAB IHC detection system (Roche Ventana, Switzerland). Haematoxylin was used as counterstain. Light microscopy images were performed with an EVOS M5000 microscope (Thermo Fisher Scientific, Germany) and insulin-stained areas served for quantification of islet beta cell areas (Fig. 7 and ESM Fig. 4) using ImageJ (version 1.53a, NIH, USA) software.

### Western blotting

NICCs and human islets were lysed in RIPA buffer (see ESM Methods). Proteins were resolved on 8–12% SDS-PAGE and blotted onto nitrocellulose membranes. Membranes were blocked in Tris Buffered Saline (TBS) supplemented with 0.15% Tween-20 and 5% milk, incubated with primary antibodies (1:1000 in TBS supplemented with 0.15% Tween and 5% BSA), followed by incubation with horseradish peroxidase-coupled secondary antibody (1:2000 in TBS supplemented with 0.15% Tween and 5% milk). Proteins were detected with ChemiDoc Touch Imaging System (BioRad Laboratories, Germany) and analysed using BioRad ImageLab software (version 5.2.1).

### Quantification and statistical analysis

Data are presented as mean ± SEM. The number (*n*) of replicates and independent experiments are given in the respective figure legends. Statistical analysis, except the RNAseq data (see ESM Methods: Transcriptome analysis), was performed with GraphPad Prism (version 8.4.0) using one-way ANOVA and Tukey’s post hoc testing. Differences were considered statistically significant at *p* ≤ 0.05. Pathways and Gene Ontology (GO) enrichment analysis of RNAseq data were performed with the web version of the Ingenuity Pathway Analysis software (Qiagen, Germany; https://analysis.ingenuity.com/pa/) and with web-based application GOrilla (cbl-gorilla.cs.technion.ac.il; access date 20.07–23.07.2020), respectively.

Islets (pig and human) were randomly distributed and blindly assigned for the treatments. For quantification, histological samples were blinded using numbers and the slides analysed by a trained scientist. No experiments and results were excluded.

## Results

### Fetuin-A inhibits functional maturation of NICCs

**Fig. 1:**
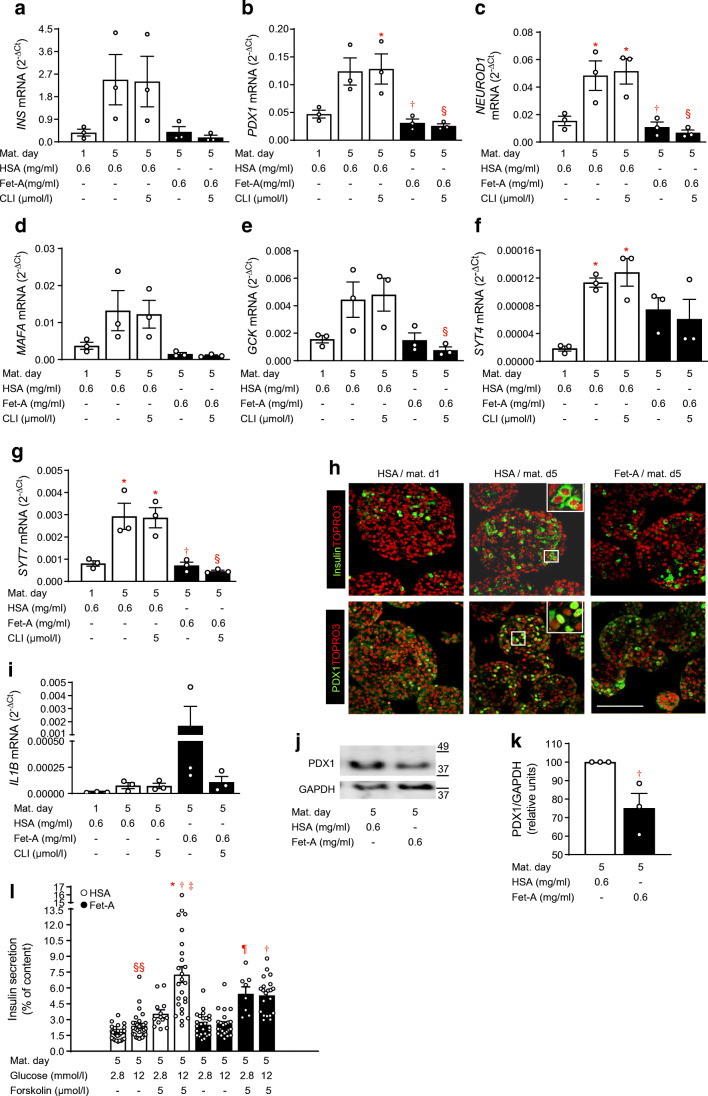
Fetuin-A impairs functional maturation of neonatal beta cells. NICCs were maturated for 5 days in maturation medium supplemented with HSA or fetuin-A and TLR4 inhibitor CLI-095 as indicated and described under Methods. (**a**–**g**; **i**) Relative mRNA levels (ΔC_t_ vs RPS13) assessed by qRT-PCR of (**a**) *INS* (encoding insulin), (**b**) *PDX1*, (**c**) *NEUROD1*, (**d**) *MAFA*, (**e**) *GCK* (encoding glucokinase), (**f**) *SYT4* (encoding synaptotagmin 4), (**g**) *SYT7* and (**i**) *IL1B*, expressed as mean ± SEM of *n* = 3 independent NICC preparations*.* (**h**) Representative confocal images from *n* = 4 independent NICC preparations immunostained for insulin (green; upper panels) or PDX1 (green; lower panels) at the beginning (HSA/mat. d1) and at the end of maturation in standard medium (HSA/mat. d5) or in the presence of fetuin-A (Fet-A/mat. d5); nuclei are stained with TOPRO3 (red); scale bar 100 μm. (**j**–**k**) Representative western blot of PDX1 in maturated NICCs in control (HSA/d5) or fetuin-A-supplemented (Fet-A/d5) medium and quantitative analysis expressed as mean ± SEM of *n* = 3 independent NICC preparations. (l) Insulin secretion expressed as % of insulin content of NICCs maturated in standard (HSA/d5; white bars) or fetuin-A-containing (Fet-A/d5; black bars) medium and presented as mean ± SEM of *n* = 8–30 replicates out of nine independent NICC preparations. (**a**–**g**, **k**) Significant differences (ANOVA) are: *p < 0.05 vs HSA/d1; ^†^*p* < 0.05 vs HSA/d5; ^§^*p* < 0.05 vs HSA + CLI/d5; (**l**) significant differences (ANOVA) are *p < 0.05 vs HSA/2.8 mmol/l glucose + forskolin; ^¶^*p* < 0.05 vs respective 2.8 mmol/l glucose (without forskolin); ^†^*p*< 0.05 vs respective 12 mmol/l glucose (without forskolin); ^‡^*p* < 0.05 vs Fet-A/12 mmol/l glucose + forskolin; ^§§^*p* < 0.01 (unpaired *t* test) vs HSA/2.8 mmol/l glucose. CLI, CLI-095; d, day; Fet-A, fetuin-A; Mat., maturation

To assess whether fetuin-A impacts on functional maturation of beta cells, NICCs were maturated in HSA- or fetuin-A-containing medium. HSA at a concentration identical to that of fetuin-A (0.6 mg/ml) was used as control, since fetuin-A and HSA are plasma components sharing similar characteristics as Ca^2+^- and fatty acid-binding proteins [25, 26]. Successful maturation was confirmed by upregulation of *INS*, *PDX1*, *NEUROD1*, *MAFA*, *GCK*, *SYT4* and *SYT7* mRNAs, paralleled by the increased number of insulin- and PDX1-positive cells (maturation d5 vs d1; Fig. 1a–h). Fetuin-A prevented upregulation of the aforementioned beta cell markers, an effect unsusceptible to the TLR4 inhibitor CLI-095. However, CLI-095 inhibited fetuin-A-induced increase of *IL1B* mRNA (Fig. 1i). Fetuin-A also reduced the number of insulin/PDX1-positive cells as well as the protein amount of PDX1 (Fig. 1h, j, k).

Since HSA concentration was rather low and fetuin-A-containing medium was albumin-free, we assessed whether HSA per se interferes with NICC maturation. Neither omission of HSA nor the increase of HSA concentration (0.6 to 1.2 mg/ml) altered NICC maturation. Moreover, addition of HSA to fetuin-A (0.6 mg/ml each) did not counteract the inhibitory effect of fetuin-A (ESM Fig. 1a–g). Despite marked upregulation of functional beta cell genes, matured NICCs (HSA/maturation d5) acquired a modest GSIS (1.4-fold increase). Nonetheless, glucose responsiveness was significantly improved (twofold increase) in the presence of forskolin, an adenylate cyclase activator that increases the intracellular level of cAMP, bypassing membrane receptors (Fig. 1l and ESM Fig. 1h). Fetuin-A-cultured NICCs were glucose unresponsive even in the presence of forskolin. Of note, forskolin-elevated insulin secretion was glucose independent (Fig. 1l).

These results suggest that fetuin-A hinders functional maturation of neonatal beta cells in a TLR4-independent manner.

### Role of TGFBR–SMAD2/3 signalling for NICC maturation

**Fig. 2:**
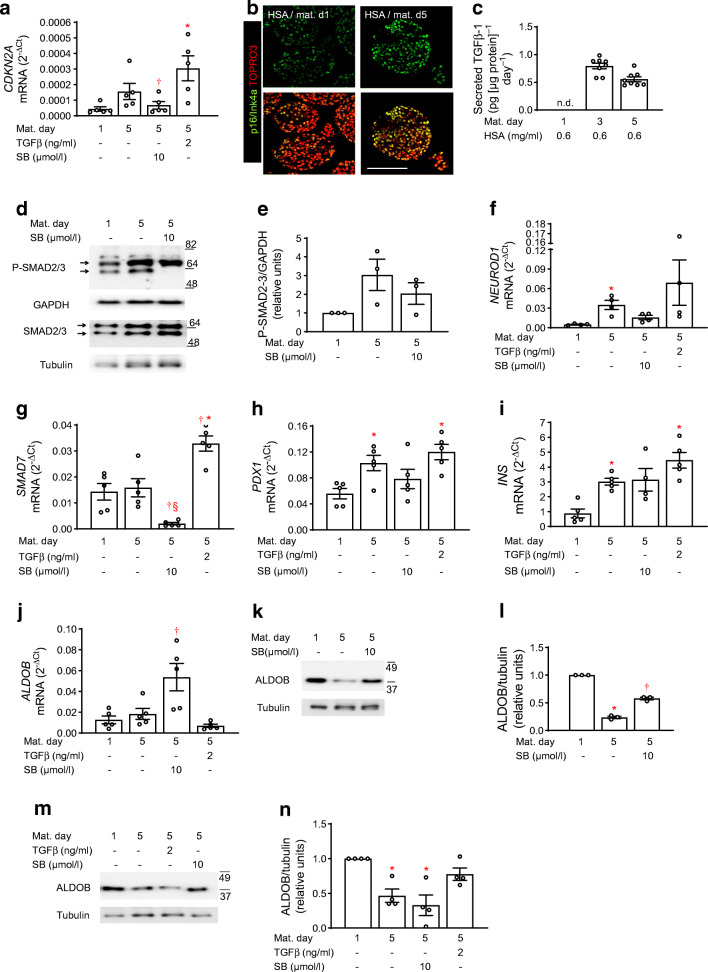
Inhibition of TGFBR signalling impairs NICC maturation. NICCs were maturated for 5 days in standard medium or in medium containing HSA + SB431542 or HSA + TGFβ-1 as indicated and described under Methods. (**a**; **f**–**j**) Relative mRNA levels (ΔC_t_ vs RPS13) assessed by qRT-PCR of (**a**) *CDKN2A* (encoding p16/Ink4a), (**f**) *NEUROD1*, (**g**) *SMAD7*, (**h**) *PDX1*, (**i**) *INS* and (**j**) *ALDOB*, expressed as mean ± SEM of *n* = 4–5 independent NICC preparations. (**b**) Representative confocal images from *n* = 4 independent NICC preparations immunostained for p16/Ink4a (green) at the beginning (HSA/mat. d1) and at the end (HSA/mat. d5) of maturation in standard medium; nuclei are stained with TOPRO3 (red); scale bar 100 μm. (**c**) TGFβ-1 (pg [μg protein]^−1^ day^−1^) secreted by NICCs into culture medium at maturation d1, d3 and d5 expressed as mean ± SEM of *n* = 8 distinct NICC preparations. (**d**, **e**; **k**–**n**) Representative western blots of (**d**, **e**) P-SMAD2/3 and SMAD2/3 and (**k**–**n**) ALDOB and respective quantitative analysis expressed as mean ± SEM of (**d**, **e**; **k**, **l**) *n* = 3 and (**m**, **n**) *n* = 4 independent NICC preparations; tubulin and GAPDH were used as loading controls. Significant effects (ANOVA) are *p < 0.05 vs maturation d1 (HSA/d1); ^†^*p* < 0.05 vs maturation d5 (HSA/d5); ^§^*p* < 0.05 vs TGFβ-1/d5. d, day; Mat. Maturation; SB, SB431542; TGFβ, TGFβ-1

Postnatal maturation of beta cells is accompanied by upregulation of p16/Ink4a (also known as cyclin-dependent kinase inhibitor 2A [encoded by *CDKN2A*]), a cell cycle inhibitor under positive regulation of TGFBR–SMAD2/3. NICC maturation (d5 vs d1) was accompanied by *CDKN2A* upregulation, while both immature (d1) and mature (d5) NICCs displayed p16/Ink4a-positive nuclei (Fig. 2a,b). Upregulation of *CDKN2A* was mediated by TGFBR–SMAD2/3 signalling, as it was inhibited by SB431542 and augmented by TGFβ-1 (Fig. 2a).

TGFBR-dependent augmentation of *CDKN2A* expression during maturation is further corroborated by TGFβ-1 accumulation in culture medium, indicating constitutive expression and release of TGFβ-1 (Fig. 2c and ESM Fig. 1i). The secreted ligand can activate TGFBR with subsequent SMAD2/3 phosphorylation and *CDKN2A* upregulation (Fig. 2a–e). On the contrary, SB431542 reduced SMAD2/3 phosphorylation, i.e. the lower SMAD2/3 phosphoprotein-band disappeared, while the upper one was minimally affected (Fig. 2d,e). *NEUROD1* and *SMAD7* were revealed to be regulated by TGFBR–SMAD2/3, since SB431542 reduced both *SMAD7* mRNA levels and the increase of *NEUROD1* mRNA levels during maturation (Fig. 2f,g). SB431542 affected neither *PDX1* nor *INS* expression (Fig. 2h,i). An active TGFBR–SMAD2/3 pathway is essential for beta cell maturation, as endorsed by the observation that SB431542 increased, while TGFβ-1 decreased, the mRNA and protein levels of aldolase B (ALDOB), a marker of functionally immature as well as of human diabetic beta cells (Fig. 2j–n) [27, 28].

These findings indicate that selective expression of beta cell markers during NICC maturation requires an active TGFBR–SMAD2/3 pathway.

### Fetuin-A impairs TGFBR–SMAD2/3 signalling in NICCs

**Fig. 3:**
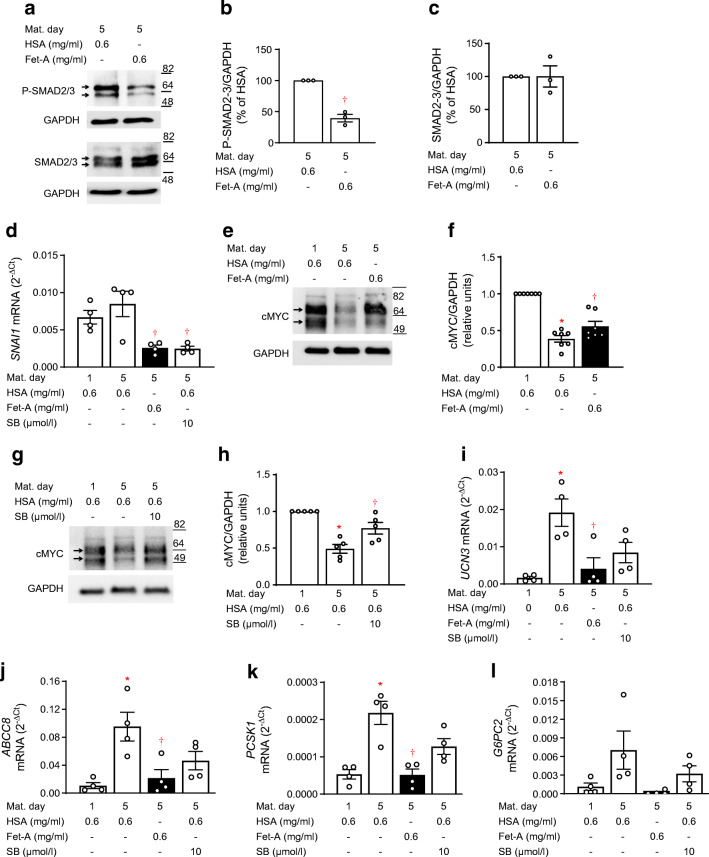
Fetuin-A inhibits TGFBR–SMAD2/3 signalling and NICC maturation. NICCs were maturated for 5 days in HSA- or fetuin-A-containing medium as described under Methods. (**a**–**c**; **e**–**h**) Representative western blots of (**a**–**c**) P-SMAD2/3 and SMAD2/3 and (**e**–**h**) cMYC and respective quantitative analysis expressed as mean ± SEM of (**a**–**c**) *n* = 3, (**e**, **f**) *n* = 7 and (**g**, **h**) *n* = 5 distinct NICC preparations. (**d**; **i**–**l**) Relative mRNA levels (ΔC_t_ vs RPS13) of (**d**) *SNAI1*, (**i**) *UCN3*, (**j**) *ABCC8*, (**k**) *PCSK1* and (**l**) *G6PC2* expressed as mean ± SEM of *n* = 4 independent NICC preparations; significant effects (ANOVA) are *p < 0.05 vs maturation d1; ^†^*p* < 0.05 vs HSA/maturation d5. d, day; Fet-A, fetuin-A; Mat. Maturation; SB, SB431542

Next, we examined whether the effect of fetuin-A on NICC maturation was mediated by inhibition of TGFBR–SMAD2/3 signalling. Indeed, fetuin-A inhibited phosphorylation of SMAD2/3 (Fig. 3a–c). In accordance, fetuin-A recapitulated the effects of SB431542 on *SNAI1* and cMYC expression, two known targets of SMAD2/3 (Fig. 3d–h). Furthermore, fetuin-A counteracted upregulation of *UCN3*, *ABCC8*, *PCSK1* and *G6PC2*, an effect also exerted by SB431542 (Fig. 3i–l).

**Fig. 4:**
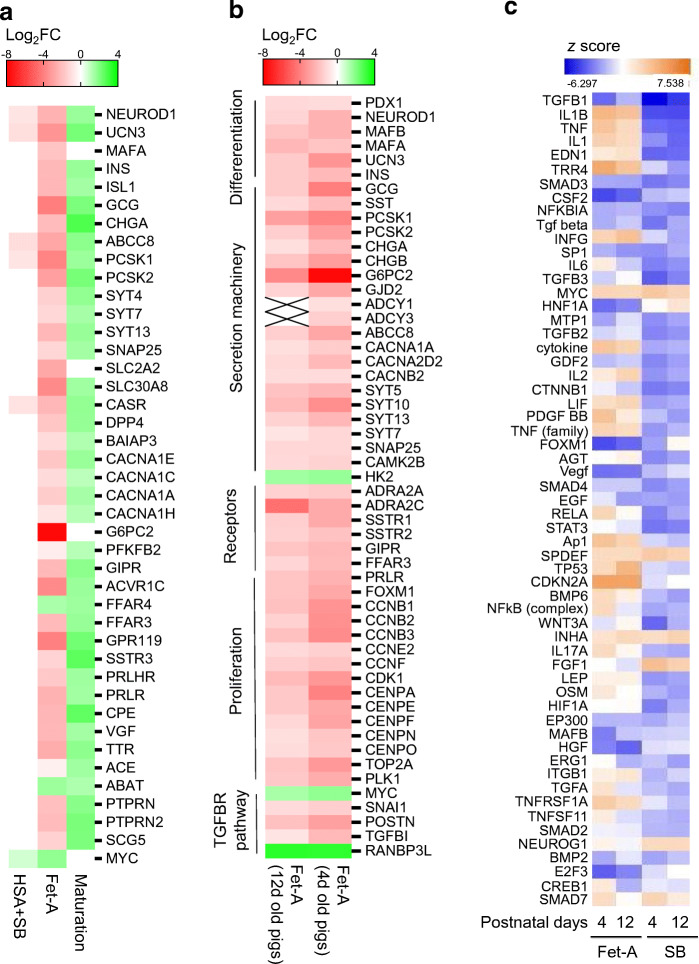
Transcriptome analysis of fetuin-A- and SB431542-treated NICCs. (**a**–**c**) RNAseq analysis of mRNA isolated from NICCs was performed as described in the Methods; (**a**) RNAseq-based heat map showing functional beta cell genes attributed to GO:0010817 (regulation of hormone levels) and upregulated (Log_2_FC > 1) in NICCs maturated in standard (HSA/d5 vs HSA/d1; *n* = 4) medium and changed (+1 > Log_2_FC < −1) by fetuin-A (Fet-A/d5 vs HSA/d5; *n* = 2) or by SB431542 (HSA + SB/d5 vs HSA/d5; *n* = 2). (**b**) Heat map showing differentially expressed (−1 > Log_2_FC > 1; Fet-A/d5 vs HSA/d5) genes in NICCs cultured with fetuin-A and isolated from 4-day-old (*n* = 2) and 12-day-old (*n* = 2) animals. (**c**) Upstream regulator analysis showing *z* score-based heat map of transcriptional regulators activated (*z* > 2) or inhibited (*z* <−2) in NICCs cultured in fetuin-A or HSA + SB431542 containing medium as indicated. NICCs were isolated from 4-day-old and 12-day-old animals. d, day; Fet-A, fetuin-A; SB, SB431542

To gain insight into the common and distinct effects of fetuin-A and SB431542 on NICC maturation, and to identify SMAD2/3-regulated genes potentially affected by fetuin-A, we performed comparative bulk RNAseq-based transcriptome analysis of NICCs cultured with fetuin-A, HSA, or HSA + SB431542. Using +1 < Log_2_FC < −1 and *p* < 0.05 adjusted for multiple comparisons as thresholds, we found 882 up- and 328 downregulated genes following maturation (HSA; d5 vs d1) (ESM Table 4). In the fetuin-A-treated NICCs (Fet-A/d5 vs HSA/d5) 423 genes were up- and 1198 were downregulated, while in the presence of SB431542, 156 and 289 genes were up- and downregulated, respectively (ESM Tables 5 and 6). In addition to the shared beta cell markers *NEUROD1, UCN3, ABCC8, PCSK1* and *CASR* (Fig. 4a), fetuin-A inhibited a larger set of genes than SB431542, including terms controlling differentiation (*PDX1*, *MAFA*, *MAFB*), insulin secretion (*G6PC2*, *SLC2A2*, *SLC30A8*, *SYT4/7/13*, membrane receptors) and adaptive proliferation (*FOXM1*, *CDK1*, *CENPA/F/E*, *TOP2A*) (Fig. 4a,b).

A comparison of the gene sets altered by SB431542 and fetuin-A revealed 172 common targets, suggesting that fetuin-A regulates these genes via inhibition of TGFBR–SMAD2/3 signalling (ESM Fig. 2a). In accordance with reduced SMAD2/3 phosphorylation, fetuin-A altered expression of typical SMAD2/3 targets, such as c*MYC*, *SNAI1*, *TGFBI* or *POSTN* (Fig. 4b). It is noteworthy that the effect of fetuin-A on gene expression was age-independent, although slightly reduced in NICCs from older piglets (12d vs 4d old animals; Fig. 4b).

To identify common contributors to the transcriptional phenotypes of fetuin-A- and SB431542-treated NICCs, the respective differentially expressed genes (DEGs) were subjected to upstream regulator analysis (Ingenuity). The analysis relies on coordinated changes which impact on downstream targets of these contributors, regardless of whether the contributor’s own expression is altered. *z* scores predict activation or inhibition of such regulators. While *TGFβ1* emerged as the top ranking contributor, the analysis identified *SMAD2/3*, *SP1*, *FOXM1* and *MAFB* as transcriptional regulators inhibited by both fetuin-A and SB431542 (Fig. 4c).

These results endorse inhibition of TGFBR–SMAD2/3-dependent transcription as an important contributor to the phenotype of fetuin-A-treated NICCs.

**Fig. 5:**
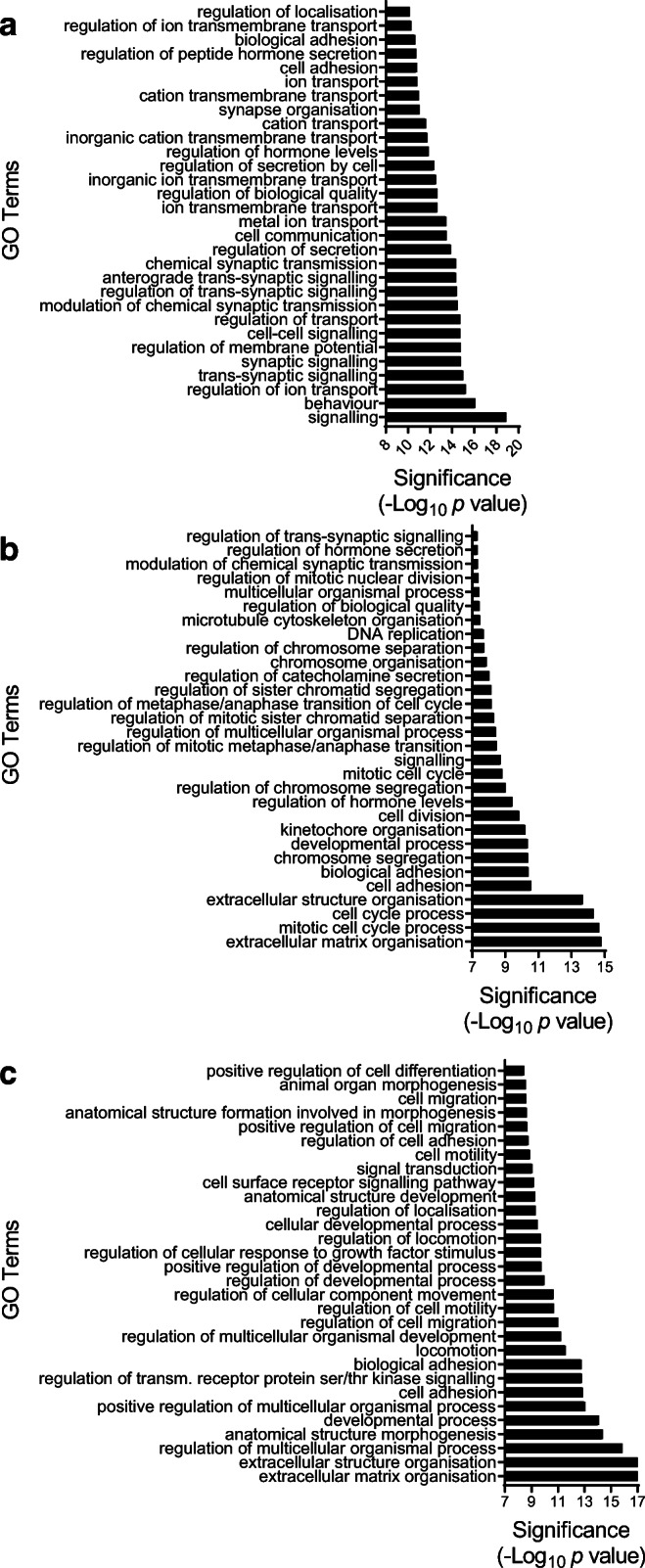
Pathway analysis of RNA sequencing in NICCs. RNAseq analysis of mRNA isolated from NICCs was performed as described under Methods. (**a**–**c**) Top 30 GO terms significantly enriched (*p* ≤ 10^−8^) in (**a**) DEGs upregulated upon maturation in standard medium and in (**b**, **c**) DEGs downregulated upon culture with (**b**) fetuin-A- and (**c**) HSA + SB431542-containing medium; false discovery rate< 2

To reveal cellular processes potentially affected by the DEGs, a GO overrepresentation analysis was performed. The maturation-upregulated gene set was enriched for terms related to insulin secretion, membrane potential, cell adhesion and cell–cell communication (Fig. 5a and ESM Fig. 2b). The fetuin-A-downregulated gene set was enriched for GO terms attributed to hormone level, cell adhesion, cell division and ECM organisation (Fig. 5b and ESM Fig. 2c). Similarly, the DEGs downregulated by SB431542 were enriched for terms associated with ECM organisation and cell adhesion (Fig. 5c and ESM Fig. 2d). Several GO terms were common for maturation-, fetuin-A- or SB431542-altered gene sets, indicating that fetuin-A and SB431542 impact on identical cellular processes, such as ECM organisation and cell adhesion. The GO ranking suggested regulation of insulin secretion as most probable consequence of gene upregulation upon maturation, while ECM organisation, cell adhesion and cell cycle processes were consequences of fetuin-A-downregulated genes (ESM Fig. 2b,c).

### Fetuin-A impairs NICC proliferation

TGFBR–SMAD2/3 signalling inhibits beta cell proliferation via induction of p16/Ink4a. While SB431542 reduced the mRNA level of *CDKN2A* (see Fig. 2a), the transcriptome analysis suggested a stimulatory effect of fetuin-A on *CDKN2A*-dependent transcription (see Fig. 4c). On the other hand, fetuin-A and SB431542 increased cMYC expression (see Fig. 3e–h), a proliferative gene under negative regulation of SMAD2/3. Therefore, we examined the effects of fetuin-A and SB431542 on beta cell proliferation in more detail.

**Fig. 6:**
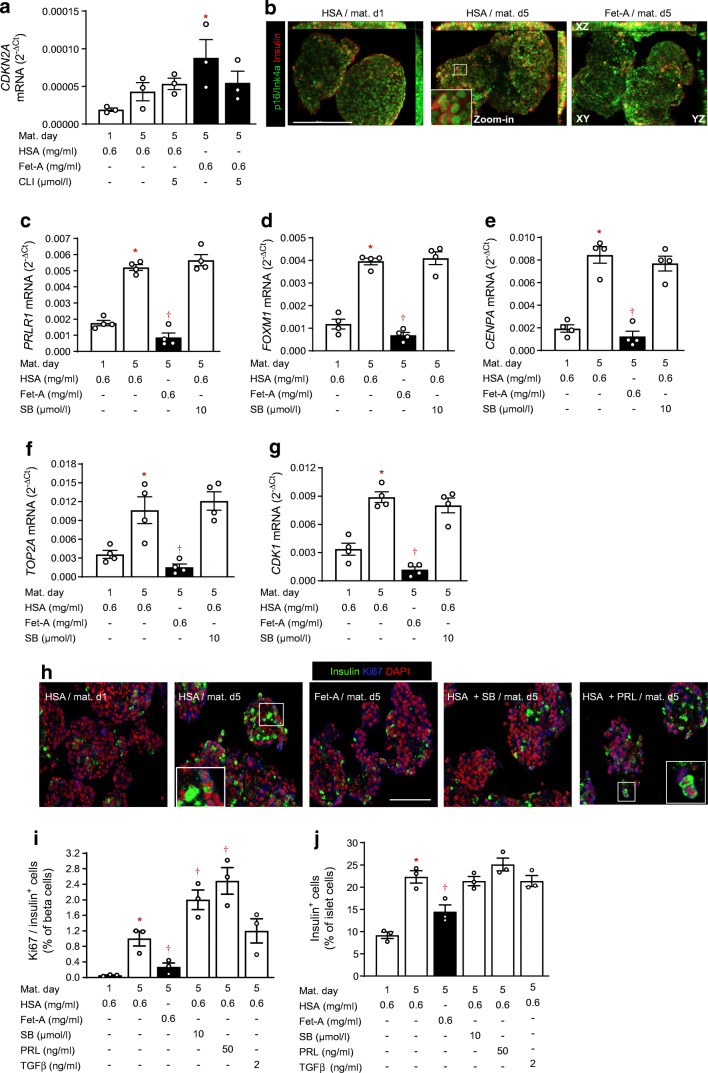
Fetuin-A inhibits beta cell proliferation. NICCs were cultured as described under Methods. (**a**; **c**–**g**) Relative mRNA levels (ΔC_t_ vs RPS13) assessed by qRT-PCR of (**a**) *CDKN2A*, (**c**) *PRLR1*, (**d**) *FOXM1*, (**e**) *CENPA*, (**f**) *TOP2A* and (**g**) *CDK1* expressed as mean ± SEM of *n* = 3–4 independent NICC preparations. (**b**) Maximum intensity projections of NICCs immunostained for p16/Ink4a (green) and insulin (red) preceding (HSA/mat. d1) and following maturation in standard medium (HSA/mat. d5) or in fetuin-A-containing medium (Fet-A/mat. d5); scale bar 200 μm. (**h**) Representative confocal microscopy pictures of NICCs immunostained for insulin (green) and Ki67 (blue) preceding (HSA/mat. d1) and following maturation in standard medium (HSA/mat. d5) or in medium containing fetuin-A (Fet-A/mat. d5), HSA + SB431542 (HSA + SB), or HSA + prolactin (10 ng/ml, HSA + PRL), as indicated; nuclei were stained with DAPI (red); scale bar 100 μm. (**i**, **j**) Percentage of (**i**) Ki67/insulin co-stained and (**j**) insulin-stained cells in NICCs cultured as indicated in (**h**). Results are expressed as mean ± SEM of *n* = 3 independent NICC preparations. Significant effects (*p* < 0.05, ANOVA) are *p< 0.05 vs immature NICCs (HSA/mat. d1); ^†^*p* < 0.05 vs maturated NICCs (HSA/mat. d5). CLI, CLI-095 (TLR4 inhibitor); d, day; Fet-A, fetuin-A; Mat., maturation; PRL, prolactin; SB, SB431542

In contrast to SB431542 and in spite of reduced SMAD2/3 phosphorylation, fetuin-A increased *CDKN2A* expression, an effect counteracted by the TLR4 inhibitor CLI-095 (Fig. 6a). Fetuin-A did not alter the cellular distribution of p16/Ink4a in beta cells (Fig. 6b). Confirming the RNAseq data (Fig. 4b), fetuin-A reduced expression of genes regulating beta cell proliferation, i.e. *PRLR1*, *FOXM1*, *CENPA*, *TOP2A* and *CDK1* (Fig. 6c–g). Distinct to fetuin-A, SB431542 did not alter the expression of these genes. In line with this expression pattern, fetuin-A reduced (0.27 ± 0.08%), while SB431542 and prolactin increased (2.00 ± 0.21% and 2.49 ± 0.28%, respectively) the number of proliferating (i.e. Ki67-positive) beta cells (Fig. 6h–i).

These findings suggest that fetuin-A limits the proliferative expansion of beta cell mass. Indeed, the fetuin-A-treated NICCs displayed significantly lower numbers of beta cells (i.e. insulin-positive), in comparison with control NICCs (14.4 ± 1.2% and 22.3 ± 1.1%, respectively) (Fig. 6j). Despite increasing beta cell proliferation, SB431542 and prolactin had no effect on beta cell number (Fig. 6j).

### Fetuin-A impairs TGFBR signalling and glucose responsiveness of adult human islets

**Fig. 7:**
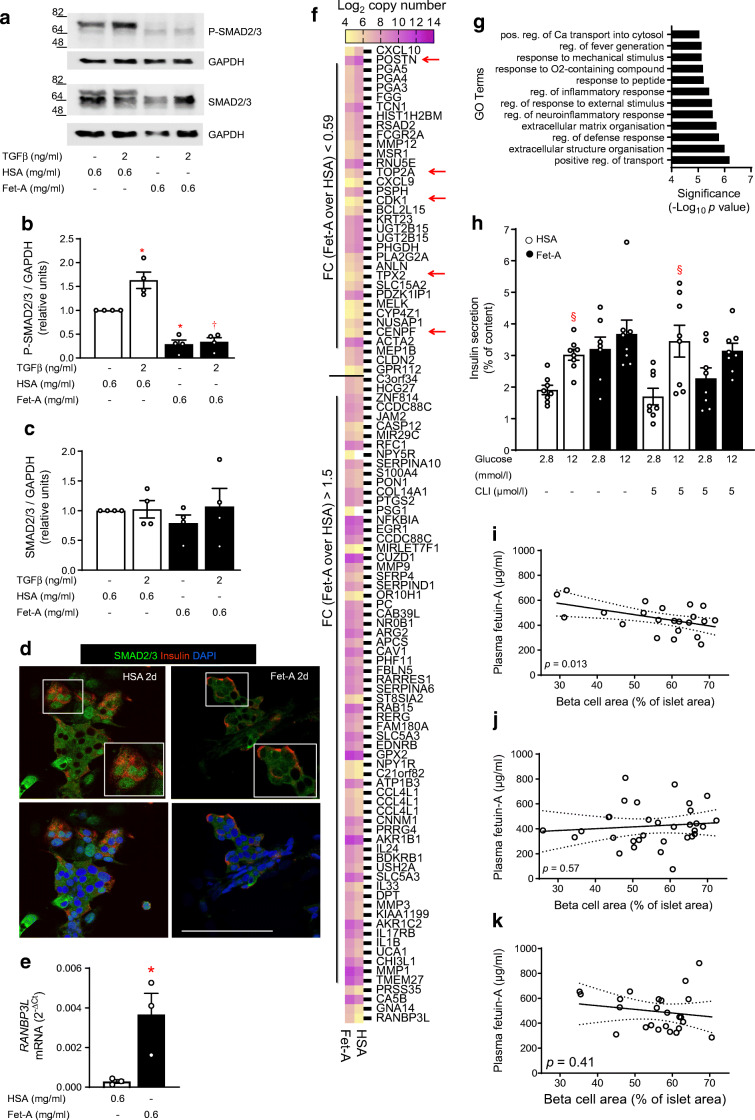
Fetuin-A inhibits TGFBR signalling and reduces functional maturity of human islets. Human islets from organ donors (ESM Table 2) were cultured for 2 days as indicated and described under Methods. (**a**–**c**) Representative western blots of P-SMAD2/3 and SMAD2/3 and respective quantitative analysis expressed as mean ± SEM of *n* = 4 independent human islet preparations. (**d**) Confocal microscopy pictures of isolated human islet cells stained for insulin (red) and SMAD2/3 (green); nuclei were stained with DAPI (blue); scale bar 100 μm. (**e**) Relative mRNA levels (ΔC_t_ vs RPS13) assessed by qRT-PCR and expressed as mean ± SEM of *n* = 3 independent preparations. (**f**) Affymetrix-based heat map showing expression level (Log_2_ copy number) of genes altered by fetuin-A (0.59 > fold change over HSA > 1.5) in isolated human islets cultured for 2 days with HSA or fetuin-A; red arrows indicate fetuin-A downregulated genes known to stimulate proliferation of beta cells. (**g**) GO terms significantly enriched in fetuin-A-altered DEGs. (**h**) Insulin secretion expressed as % of content presented as mean ± SEM of *n* = 8 replicates out of two independent human islet preparations. (**i**–**k**) Beta cell area was assessed in insulin-stained human pancreatic resections as described in the Methods. Correlation of beta cell area expressed as % of islet area with plasma levels of fetuin-A in (**i**) *n* = 22 non-diabetic human donors, (**j**) *n* = 32 donors with IGT/IFG and (**k**) *n* = 24 type 2 diabetic donors. Significant effects (*p* < 0.05, ANOVA) *p < 0.05 vs HSA; ^†^*p* < 0.05 vs HSA + TGFβ-1; ^§^*p* < 0.05 vs respective 2.8 mmol/l glucose. CLI, CLI-095 (TLR4 inhibitor); d, day; FC, fold change; Fet-A, fetuin-A; TGFβ, TGFβ-1

In order to translate these results to humans we examined whether fetuin-A impacts on TGFBR–SMAD2/3 signalling in isolated islets from organ donors. In human islets cultured for 2 days with fetuin-A, basal and TGFβ-1-stimulated SMAD2/3 phosphorylation were inhibited (Fig. 7a–c). In accordance, fetuin-A reduced nuclear accumulation of SMAD2/3 in cultured islet cells (Fig. 7d). An Affymetrix-based transcriptome analysis of human islets cultured for 2 days with fetuin-A revealed *RANBP3L* as the top upregulated gene, while *POSTN*, *CDK1*, *CENPF*, *TOP2A* and *TPX2* occupied prominent positions among the downregulated ones (Fig. 7f). Of note, the qRT-PCR analysis confirmed the fetuin-A-induced upregulation of RAN binding protein 3L (*RANBP3L*), a protein that regulates nuclear–cytosolic shuttling of SMADs (Fig. 7e, [29]). The fetuin-A-altered gene set was enriched in GO terms related to calcium transport and ECM organisation, and the GO ranking suggests calcium transport into the cytosol as functional consequence (Fig. 7g and ESM Fig. 3). Fetuin-A abolished glucose responsiveness of human islets (from 1.7-fold increase of secretion [*p* < 0.05] to 1.1-fold [*p* > 0.05]) by increasing basal secretion, an effect maintained in the islets co-treated with the TLR4 inhibitor CLI-095 (Fig. 7h). Notably, we found a negative correlation of islet beta cell area with the level of plasma fetuin-A in non-diabetic humans (*p* = 0.013; Fig. 7i–k and ESM Fig. 4).

These results indicate that fetuin-A impairs functional maturity (i.e. GSIS, and TGFBR signalling) in adult islets. In addition, fetuin-A might compromise the adaptive increase of functional beta cell mass.

## Discussion

Investigating maturation of porcine neonatal islets, we found that fetuin-A disrupts both functional maturation and adaptive proliferation of beta cells. Fetuin-A inhibits TGFBR–SMAD2/3 signalling, a pathway essential for adequate expression of critical functional genes, while it diminishes beta cell proliferation in a TGFBR–SMAD2/3-independent manner.

### TGFBR–SMAD2/3 pathway sustains functional maturation of beta cells

During maturation, NICCs express and secrete TGFβ-1 which, in an autocrine/paracrine manner, stimulates TGFBR–SMAD2/3-dependent transcription, increasing *CDKN2A* expression. Thus, TGFBR–SMAD2/3 inhibition by SB431542 counteracted upregulation of *CDKN2A*, but also that of *NEUROD1*, *UCN3* and *ABCC8*. Of note, TGFBR–SMAD2/3 signalling repressed *ALDOB*, a disallowed gene in mature beta cells and a marker of functionally immature as well as of diabetic beta cells [8, 27, 28]. These results document a positive role of the TGFBR–SMAD2/3 pathway for functional maturation of beta cells. In accordance, mice with beta cell-specific deletion of *Smad2* display defective GSIS and overt diabetes along with increased islet proliferation and hyperplasia [30]. Likewise, iPSC-derived beta cells require active TGFBR–SMAD2/3 signalling for functional maturation, i.e. gain of glucose-responsive insulin secretion. In line with this, iPSC-derived beta cells exposed to SB431542 failed to restore normoglycaemia in recipient streptozotocin (STZ)-treated mice [14, 31].

### Fetuin-A impairs functional maturation of beta cells via inhibition of TGFBR signalling

The modest GSIS of matured NICCs is in accordance with previous observations, while transplantation augments their functional performance [22, 32–34]. However, glucose responsiveness was improved upon elevation of cellular cAMP via forskolin-induced activation of adenylate cyclase. A similar dependence of GSIS on cAMP has been previously observed in human neonatal islets and in adult islet cells [1, 2, 35].

Fetuin-A selectively inhibits GSIS and functional maturation of NICCs via modulation of gene expression. The reduced expression of genes governing endocrine identity and maturation (*PDX1,* *NEUROD1,* *UCN3*), cell connectivity (*GJD2*), hormone processing (*PCSK1/2*), stimulus-secretion coupling (*ABCC8*), calcium sensing (*SYT4/7*) and exocytosis (*SNAP25*) convey the inhibitory effect of fetuin-A on GSIS. An inadequate cell–cell communication owing to low *GJD2* expression may contribute to the low glucose responsiveness, since connexion-36 expression is required for Ca^2+^ oscillations and GSIS [36].

The transcriptome analysis identified inhibition of TGFβ-1–SMAD2/3-regulated transcription as an essential contributor to the phenotype of fetuin-A-treated NICCs. A positive role of TGFBR–SMAD2/3 for beta cell function is further substantiated by the increased expression of c*MYC*, a well-known target of TGFBR–SMAD2/3, upon inhibition of TGFBR either with SB431542 or with fetuin-A [37]. Particularly, the upregulation of cMYC in adult beta cells was reported to generate a neonatal-like phenotype, i.e. increased basal secretion and loss of GSIS [5, 38]. Fetuin-A-treated human islets display increased basal secretion, allowing the assumption that fetuin-A enforces a dematuration process, as increased basal secretion is characteristic of immature islets as well as of islets of humans with type 2 diabetes [1, 39]. In line with this, in human islets fetuin-A upregulated expression of *TMEM27*, a gene recently associated with beta cell juvenile phenotype [40].

We found an extensive fetuin-A- and SB431542-induced dysregulation of genes related to cell–cell communication, cell adhesion and ECM. TGFBR signalling is in fact the master regulator of ECM organisation, while ECM components such as matrix metalloproteinases (MMPs) activate latent TGFβ ligands. Moreover, there is increasing evidence that islet ECM determines beta cell function [41, 42]. Therefore, fetuin-A-induced inhibition of TGFBR signalling may impact on beta cell function also via alterations of islet ECM. One of the top genes downregulated by fetuin-A was the SMAD3 target *POSTN* (which encodes periostin), an ECM component secreted by islet pericytes and stellate cells. Whether ECM remodelling underlies the inhibitory effect of fetuin-A on human islet function needs further experimental evidence. Indeed, periostin was found to sustain beta cell regeneration and function recovery in STZ-treated mice [43–45].

Fetuin-A reduced phosphorylation and nuclear localisation of SMAD2/3 in human islet cells, and it upregulated *RANBP3L*, a chaperone involved in nuclear export of SMADs [29]. Since the epigenetic landscapes of neonatal and adult beta cells differ considerably, and SMAD action is subjected to epigenetic regulation [8], it is reasonable to assume that the fetuin-A-modulated TGFBR–SMAD pathway yields distinct outcomes in adult and neonatal islets. Yet, according to the GO ranking, cytosolic Ca^2+^ trafficking and ECM organisation are functional consequences of fetuin-A-altered gene expression in human islets. We previously showed that fetuin-A alters Ca^2+^ sensitivity of insulin secretion in adult human islets, an observation in conformity with its Ca^2+^-binding ability [21].

### TGFBR–SMAD2/3 inhibition and fetuin-A exert opposing effects on beta cell proliferation

Neonatal beta cells are proliferative and functionally immature [2, 46]. To achieve full glucose-responsive insulin secretion they undergo a maturation process accompanied by rapid decline of proliferative capacity [1, 40]. To the contrary, adult beta cells are differentiated and refractive toward proliferation, and they acquire an immature neonatal-like phenotype when primed to undergo cell cycling, i.e. following expression of non-oncogenic levels of cMYC [5]. In this respect, our results showing that fetuin-A diminished both functional maturation and proliferation of NICCs were unexpected.

Fetuin-A-induced upregulation of c*MYC* did not suffice to boost beta cell proliferation, since expression of *CDKN2A*, the potent inhibitor of beta cell proliferation, remained high [47]. Of note, the TLR4 inhibitor CLI-095 counteracted the fetuin-A-mediated increase of *CDKN2A* (see Fig. 6a). Fetuin-A is an endogenous ligand of TLR4 [19]; therefore contribution of this receptor to the effect of fetuin-A on *CDKN2A* cannot be excluded. Fetuin-A, a physiological inhibitor of insulin receptor (IR), reduced expression of *FOXM1*, a transcription factor acting downstream of IR and a master regulator of a proliferative pathway comprising *TOP2A*, *CDK1*, *PLK1* and *CENPA* and also operating in human adult beta cells. Unlike cMYC-dependent proliferation, FOXM1-driven proliferation does not harm beta cell function, thereby allowing compensatory increase of functional beta cell mass [5, 48]. Indeed, we found a reduced number of proliferative beta cells in fetuin-A-treated NICCs.

*TOP2A*, *CDK1*, *CENPF* and *TPX2* (red arrows, Fig. 7f) are top upregulated genes in proliferating beta cells, while reduced *TPX2* expression was recently found in human islets exposed to glucolipotoxicity [49, 50]. Downregulation of these genes in fetuin-A-treated human islets suggests a persistent inhibitory effect of fetuin-A on proliferation. In line with this, in non-diabetic humans, plasma fetuin-A level was negatively correlated with beta cell area, suggesting that fetuin-A constrains expansion of functional beta cell mass required by an increased insulin demand.

Distinct to fetuin-A, SB431542 reduced *CDKN2A* expression and increased NICC proliferation, suggesting that *CDKN2A* downregulation is mandatory for beta cell proliferation [47]. However, inhibition of TGFBR impaired functional maturity of beta cells, i.e. cMYC and ALDOB upregulation, which might explain why SB431542 did not increase beta cell number (Fig. 6j). Thus, inhibition of this pathway is an unsuitable strategy for increasing beta cell mass.

In conclusion, we propose that fetuin-A is a negative modulator of function and proliferation of beta cells. The perinatal drop in fetuin-A production favours postnatal maturation and proliferation of neonatal beta cells. During adult life, a chronic fatty liver-induced elevation of serum fetuin-A impairs both functional maturity and the adaptive increase of adult beta cell mass, thereby accelerating the onset of type 2 diabetes.

## Supplementary Information

ESM (PDF 2.93 mb)

## Data Availability

The RNAseq datasets and computer code produced in this study are available in the Gene Expression Omnibus (GEO): GSE144950; https://www.ncbi.nlm.nih.gov/geo/query/acc.cgi?acc=GSE144950

## Abbreviations

ALDOB: Aldolase B
BMP: Bone morphogenic protein
DEG: Differentially expressed genes
ECIT: European Consortium for Islet Transplantation
ECM: Extracellular matrix
GO: Gene Ontology
GSIS: Glucose-stimulated insulin secretion
HSA: Human serum albumin
IGT: Impaired glucose tolerance
IFG: Impaired fasting glucose
iPSC: Induced pluripotent stem cell
IR: Insulin receptor
Log_2_FC: Log_2_ fold change
NICC: Neonatal islet cell cluster
RNAseq: RNA sequencing
qRT-PCR: Reverse transcription quantitative real-time PCR
STZ: Streptozotocin
TGFBR: TGFβ receptor
TLR4: Toll-like receptor 4
