# Supplementary material for: The hepatokine fetuin-A disrupts functional maturation of pancreatic beta cells
Source: Diabetologia. 2021 Mar 25;64(6):1358–74. doi: 10.1007/s00125-021-05435-1 (PMC8099843; doi:10.1007/s00125-021-05435-1)
Supplement: Supplementary file 1 — (PDF 2.93 mb) [file 125_2021_5435_MOESM1_ESM.pdf]

# *Electronic Supplementary Material*

**Isolation and culture of porcine neonatal islet cell clusters**

Freshly isolated NICCs were cultured in petri dishes containing maintenance medium (1:1 mixture of Ham's F12 and medium 199) containing (in mmol/l): 10 glucose, 10 L-glutamine, 0.5 Pefabloc, 10 HEPES, 10 nicotinamide, 0.5% BSA, 0.062% glutathione, 100 U/ml penicillin, and 0.1 mg/ml streptomycin, 2.5 µg/ml amphotericin, 25 U/ml heparin, 100 KIU aprotinin, 10 µmol/l TroloxTM, 1x BME vitamins, 15.2 µmol/l zinc sulphate and 1 µmol/l liraglutide. Since FCS contains high amounts of fetuin-A, NICCs maturation was conducted in FCS-free medium. At culture day 5, NICCs were placed in FCS-free Ham's F10 maturation medium containing 10 mmol/l glucose, 50 µmol/l isobutylmethylxanthine, 2 mmol/l L-glutamine, 10 mmol/l nicotinamide, 1.25 mmol/l $CaCl_2$, 100 U/ml penicillin, 0.1 mg/ml streptomycin and supplemented with 0.6 mg/ml human serum albumin (HSA).

**Isolated human islets**

The isolated human islets were cultured overnight in CMRL1066 medium supplemented with (in mmol/l): 5 glucose, 2 L-glutamine, 10 HEPES, and 10% FCS.

**Transcriptome analysis**

The mRNA samples were then subjected to the workflow for strand specific RNAseq library preparation (Ultra Directional RNA Library Prep II, NEB). Following ligation, adapters were depleted by 1X XP bead purification. Indexing was carried out during the following PCR enrichment (15 cycles, 65 °C). The libraries were quantified using Qubit dsDNA HS Assay Kit (Invitrogen) and used for 75bp single read sequencing on Illumina NextSeq 500, resulting in an average of 28 Mio sequenced fragments per sample. For the bioinformatics analysis, the workflow nf-core/rnaseq (https://nf-co.re/rnaseq, release 1.2) was set-up as following: FASTQC was used to determine the quality of the FASTQ files; adapter and quality-based trimming were subsequently performed with Trim Galore; STAR aligner (v2.6.1) was used to

map the quality controlled reads to the pig genome (Ensembl release 94). Read counting on the features was performed with feature counts. MultiQC (v1.6) was used to aggregate the final results into one html report. For differential expression analysis, the row read count table resulting from the nf-core/rnaseq workflow was processed with the R package DESeq2 v1.22.1.

**Western blotting**

NICCs and human islets were lysed in RIPA buffer containing (mmol/l): 25 Tris/HCl (pH 7.5), 150 NaCl, 2 EDTA, 10 NaF, 1 Na3VO4, 10% glycerol, 1% Nonidet-P40, 0.1% SDS, 0.1% C24H39NaO4 and protease inhibitors.

**ESM Table 1 Reagents and Resources.**

**ESM Table 2 Human islets checklist: characteristics of the isolated human islets and islets donors.**

**ESM Table 3 Characteristics of the human donors of pancreatic resections.**

**ESM Table 4 Differentially expressed genes in NICCs following in vitro maturation.** RNA sequencing results showing all genes with a significantly altered expression level (base Mean >50; -1>log2FC>1; p adjusted < 0.05) in NICCs maturated in standard medium (HSA/d5 versus HSA/d1).

**ESM Table 5 Differentially expressed genes in NICCs isolated from 4 days old animals upon culture  the presence of fetuin-A.** RNA sequencing results showing all genes with a significantly altered expression level (base Mean > 50; -1>log2FC>1; p adjusted < 0.05) in NICCs isolated from 4d old animals and cultured in medium with fetuin-A (Fetuin-A/d5 versus HSA/d5).

**ESM Table 6 Differentially expressed genes in NICCs isolated from 4 days old animals following in vitro maturation in the presence of TGFBRI inhibitor SB431542.** RNA sequencing results showing all genes with a significantly altered expression level (base mean

>50, -1>log2FC>1; p adjusted < 0.05) in 4 days old NICCs maturated in the presence of SB431542 (HSA + SB/d5 versus HSA/d5).

ESM Table 1

| REAGENT or RESOURCE | REFRENCE OR SOURCE | IDENTIFIER |
|---|---|---|
| **Antibodies** | | |
| ALDOB | ProteinTech Group, Manchester, UK | 18065-1-AP |
| SMAD2/3 | Cell Signaling Technology, Massachusetts, USA | 5678 |
| P-SMAD2 (Ser465/467) / P-SAMD3 (Ser423/425) | Cell Signaling Technology | 8828 |
| PDX1 | Abcam, Cambridge, UK | 47383 |
| P16/Ink4a | Santa Cruz Biotechnology, Texas, USA | 1661 |
| cMYC | Cell Signaling Technology | 5605 |
| Ki67 | Abcam | 15580 |
| Insulin | Abcam | 8304 |
| Insulin | Dako Agilent, CA, USA | A0564 |
| GAPDH | Cell Signaling Technology | 2118 |
| Tubulin | Cell Signaling Technology | 2148 |
| Goat anti-Mouse IgG Secondary Antibody, Alexa Fluor 488 | Thermo Fischer Scientific, Massachusetts, USA | A28175 |
| Goat anti-rabbit IgG Secondary Antibody, Alexa Fluor 488 | Thermo Fischer Scientific | A11008 |
| Goat anti-Rabbit IgG Secondary Antibody, Alexa Fluor 546 | Thermo Fischer Scientific | A11035 |
| Opti-View DAB IHC detection | Roche, Switzerland | 760-700 |
| Horse radish peroxidase-coupled secondary antibody | GE Healthcare, Germany | NA934 |
| **Chemicals, Peptides, and Recombinant Proteins** | | |
| Fetuin-A (α2-hs-Glycoprotein from human plasma) | Sigma Aldrich, Missouri, USA | G0516 |
| Human Serum Abumin 20% (HSA) | CSL Behring, Marburg, Germany | |
| Bovine Serum Albumin (BSA- fraction V) | Sigma Aldrich | A3294 |
| Forskolin | Sigma Aldrich | F6886 |
| DAPI | Sigma Aldrich | D9542 |
| TOPRO3 | Invitrogen | N1 |
| SB431542 | Merck Millipore, Massachusetts, USA | 566405 |
| Palmitic acid | Sigma Aldrich | P0500 |

| | | |
|---|---|---|
| CLI-095 | InvivoGen,<br>California, USA | Tlrl-cli95 |
| Human TGFβ-1 | R&D Systems,<br>Minnesota, USA | 100-B |
| Human Recombinant Prolactin | R&D Systems | 682-PL |
| Collagenase V | Sigma Aldrich | C9263 |
| Illumina NextSeq 500 | Illumina,<br>California, USA | |
| Porcine Insulin ELISA | Mercodia,<br>Uppsala, Sweden | 10-1200-01 |
| Transcriptor First Strand kit | Roche, Basel,<br>Switzerland | 04379012001 |
| Human TGFβ-1 ELISA | R&D Systems | DB100B |
| **Deposited Data** | RNAseq Data | GEO:<br>GSE144950 |
| **Oligonucleotides** | | |
| *Sus Scrofa* | | |
| INS<br>F 5’-GTGGCATCGTGGAGCAGT-3’<br>R 5‘-CGGCCTAGTTGCAGTAGTTCTC-3’ | | |
| PDX1<br>F 5’-TGTGGTTTCCAGATTGCTTG-3’<br>R 5‘-TCCACAAGCATCACCTGAAC-3‘ | | |
| MAFA<br>F 5‘-TGCCAGAAAGAGCCAAGAA-3‘<br>R 5‘-GTAGGAGGTGGCGTCGTG-3‘ | | |
| NEUROD1<br>F 5’-AAGCCATGAATGCGGAAG-3’<br>R 5’-TCCTCTTCCAAGTCCTCGTC-3’ | | |
| CDKN2A<br>F 5’-CACTTTGGTGGTCCTGCAC-3’<br>R 5’-CCCTCTCCTCAGCCAGGT-3’ | | |
| SMAD7<br>F 5’-TCAGTGCCACCAAACACAGT-3’<br>R 5’-GCTACGATGACCCCAAGGT-3’ | | |
| PRLR<br>F 5’-TCCTACCAACTACACGCTGACTT-3’<br>R 5’-GTCTGGACATTCATGGGTGA-3’ | | |
| FOXM1<br>F 5’-TCAAGACACCCGTTAAGGAGA-3’<br>R 5’-ATGGGGAGGACAGATTTGCT-3’ | | |
| CCNB1<br>F 5’-GGCTCTTCTTGTTTTATGTACTTGG-3’<br>R 5’-CAAGATGACTGAGCTTACATACAGTG-3’ | | |
| CENPA<br>F 5’-TGACCAGACAACGTGCATCT-3’<br>R 5’-TCATCTGGTCTTTGTGAAGTGG-3’ | | |
| CDK1<br>F 5’-GACGCTGACGTGGTAGCC-3’<br>R 5’-GGATGTGGTAGATCCCAGCTTA-3’ | | |
| TOP2A<br>F 5’-TCCACTGAGTGTACGCTTATCC-3’<br>R 5’-GGCCTGAAACTGCCAAAGT-3’ | | |

| | | |
|---|---|---|
| ABCC8<br>F 5’-TACGCCATGGTGTTCACAGT-3’<br>R 5’-AGACGTGACAAGGCACAGC-3’ | | |
| G6PC2<br>F 5’-TCATGTGTCTGGTATGTCATGG-3’<br>R 5’-ATCCATCCACCTGATGGTGT-3’ | | |
| UCN3<br>F 5’-TCACAGGCATTTCCACGAT-3’<br>R 5’-GGGCTGCAATCAGCATCT-3’ | | |
| GJD2<br>F 5’-CACTGCAGCTCGATCCAAG-3’<br>R 5’-TGGATAATGTAGAAGCGGGAGA-3’ | | |
| PCSK1<br>F 5’-ACCCTACCTCATGGCAACAG-3’<br>R 5’-TTCAAGACTGGCTGGGTAGC-3’ | | |
| SNAI1<br>F 5’-ACTTGTTGAGGGCCCAAGT-3’<br>R 5’-CAGAAATAGTTCCGGGAGACA-3’ | | |
| GCK<br>F 5’-CTGGCTGCAGTACAGATGCT-3’<br>R 5’-CTTTTCCTTCTTGGCGCTCT-3’ | | |
| SYT4<br>F 5’-ATTGCGTCAATTCGGGTTT-3’<br>R 5’-TGTTCCTGTTTTGGCTCTTCA-3’ | | |
| SYT7<br>F 5’-GGCGTCCGGACACTACAG-3’<br>R 5’-CCCATCGGGAATGGAACT-3’ | | |
| IL1B<br>F 5’-GGCAGATGGTGTCTGTCATC-3’<br>R 5’-CCTGGGAGGAGGGATTCTT-3’ | | |
| RBP3L<br>F 5’-GAAAATGTACCAAGTGCCAGAA-3’<br>R 5’-TGTAGCACCTAAAACATTCTGGTTAG-3’ | | |
| *Homo Sapiens* | | |
| FOXM1<br>F 5’-ACTTTAAGCACATTGCCAAGC-3’<br>R 5’-CGTGCAGGGAAAGGTTGT-3’ | | |
| PRLR<br>F 5’-CCAGCGACCTTCATTCAGATA-3’<br>R 5’-GCCACTGCCCAGACAATAAT-3’ | | |
| TMEM27<br>F 5’-TTGTGGTTACAGACCCTTCAAA-3’<br>R 5’-GCATTGTTGATCCGGTTCTT-3’ | | |
| G6PC2<br>F 5’-TGTCTGTGGGATGGATAAGTTCT-3’<br>R 5’-GCCTGGAGTGTGTTCAAAGG-3’ | | |
| SLC30A8<br>F 5’-AGTAATTCTCTCAGCTCATGTTGC-3’<br>R 5’-GCAATTTCTCTCCGAACCAC-3’ | | |
| PCSK1<br>F 5’-CGGGGTTGGAGTTGCATA-3’<br>R 5’-CCTCAATAGCATCCGTCACA-3’ | | |

| | | |
|---|---|---|
| UCN3<br>F 5’-ACAAGTTCATGGGGACGTG-3’<br>R 5’-CGGCATCAGCATCTCTCC-3’ | | |
| **Software** | | |
| Image Lab 5.2.1 | BioRad Laboratories, Feldkirchen, Germany | |
| Graph Pad 8.4.0 | NA | |
| Ingenuity Pathway Analysis Software<br>https://analysis.ingenuity.com/pa/ | Qiagen, Hilden Germany | |
| R package DESeq2 v1.22.1 | NA | |

ESM Table 2

| Islet preparation | 1 | 2 | 3 | 4 | 5 | 6 | 8 | 9 |
|---|---|---|---|---|---|---|---|---|
| Unique Identifier | HP1012 | H1267 | H993 | HP1354 | HI9 | HI1326 | HI1332 | HI1333 |
| Donor Age (years) | 55 | 54 | 63 | 58 | 59 | 63 | 62 | 54 |
| Donor sex (M/F) | M | M | M | M | F | F | F | M |
| Donor BMI (kg/m2) | 23.5 | 26.5 | 25.9 | 27.2 | 23.7 | 21.8 | 29.3 | 24.2 |
| Source of islets | ECIT | ECIT | ECIT | ECIT | ECIT | ECIT | ECIT | ECIT |
| Isolation center | Lille | Milano | Lille | Milano | Geneva | Milano | Milano | Milano |
| Donor's history of diabetes | no | no | no | no | no | no | no | no |
| Purity (%) | 90 | 75 | 80 | 80 | 84 | 85 | 90 | 90 |
| Viability (%) | 95 | 95 | 98 | 90 | 90 | 95 | 95 | 95 |
| Total culture time (d) | 8 | 6 | 6 | 7 | 8 | 5 | 6 | 6 |

ESM Table 3. Charactersitics of the donors of human pancreatic resections

| Donor identifier | SEX | AGE years | BMI | Fasting glucose mg/dl | Fasting insulin pmol/l | HbA1c % | Plasma fetuin-A µg/ml | Beta cell area % islet area | |
|---|---|---|---|---|---|---|---|---|---|
| P1 | f | 57 | 29.7 | 78 | 84 | 5.6 | 555.90 | 68.38 | ND |
| P2 | f | 79 | 27.1 | 66 | 15 | 4.7 | 680.60 | 31.85 | ND |
| P3 | 1 | 74 | 25.0 | 83 | 68 | 5.2 | 245.99 | 67.80 | ND |
| P4 | f | 63 | 22.0 | 85 | 90 | 4.7 | 592.66 | 52.86 | ND |
| P5 | m | 54 | 27.8 | 98 | 110 | 5.1 | 537.51 | 57.45 | ND |
| P6 | m | 33 | 24.2 | 100 | 62 | 4.8 | 408.16 | 46.80 | ND |
| P7 | m | 68 | 27.5 | 93 | 105 | 5.5 | 421.08 | 64.57 | ND |
| P8 | f | 77 | 29.0 | 89 | 61 | 5.4 | 428.73 | 61.93 | ND |
| P9 | f | 72 | 29.1 | 80 | 35 | 4.9 | 462.99 | 31.18 | ND |
| P10 | m | 49 | 31.1 | 97 | 51 | 5.1 | 500.90 | 41.82 | ND |
| P11 | m | 46 | 26.1 | 93 | 48 | 5.2 | 567.10 | 64.85 | ND |
| P12 | m | 75 | 23.9 | 89 | 55 | 5.2 | 498.76 | 52.42 | ND |
| P13 | m | 59 | 25.6 | 72 | 19 | 5.5 | 467.58 | 65.75 | ND |
| P14 | m | 36 | 26.6 | 82 | 155 | 5.6 | 439.88 | 71.53 | ND |
| P15 | f | 84 | 22.0 | 77 | 23 | 4.6 | 296.74 | 56.00 | ND |
| P16 | m | 65 | --- | 88 | 60 | 5.5 | 304.91 | 66.81 | ND |
| P17 | m | 63 | 23.5 | 95 | 102 | 5.5 | 442.44 | 56.41 | ND |
| P18 | m | 59 | 24.1 | 100 | 80 | 5.1 | 646.60 | 29.23 | ND |
| P19 | f | 58 | 20.3 | 74 | 24 | 5.1 | 285.56 | 60.53 | ND |
| P20 | f | 52 | 23.2 | 85 | 48 | 5.5 | 431.38 | 68.67 | ND |
| P21 | f | 37 | 21.0 | 88 | 39 | 5.5 | 356.96 | 61.69 | ND |
| P22 | f | 72 | 19.5 | 96 | 41 | 5.6 | 435.15 | 60.62 | ND |
| P23 | f | 54 | 22.5 | 105 | 67 | 5.2 | 432.96 | 65.30 | IFG |
| P24 | f | 66 | 24.2 | 113 | 47 | 5.6 | 251.02 | 50.36 | IFG |
| P25 | m | 60 | 27.9 | 122 | 95 | 5.5 | 664.16 | 69.93 | IFG |
| P26 | m | 55 | 25.9 | 107 | 128 | 5.3 | 546.05 | 65.47 | IFG |
| P27 | m | 70 | 23.7 | 102 | 51 | 5.5 | 612.03 | 51.16 | IFG |
| P28 | m | 61 | 25.3 | 117 | 72 | 5.9 | 202.48 | 46.43 | IGT |
| P29 | m | 64 | 24.8 | 125 | 43 | 5.0 | 328.33 | 44.79 | IFG |
| P30 | m | 76 | 25.0 | 107 | 88 | 6.4 | 385.66 | 66.57 | IGT |
| P31 | m | 64 | 25.8 | 103 | 70 | 6.4 | 338.34 | 65.89 | IGT |
| P32 | m | 49 | 23.5 | 104 | 64 | 5.7 | 496.53 | 43.73 | IGT |
| P33 | f | 76 | 16.9 | 106 | 45 | 5.9 | 380.57 | 36.26 | IGT |
| P34 | m | 50 | 21.0 | 102 | 34 | 5.3 | 605.33 | 65.15 | IFG |
| P35 | m | 66 | 24.8 | 115 | 46 | 6.1 | 329.79 | 64.61 | IGT |
| P36 | m | 56 | 19.5 | 102 | 25 | 6.1 | 300.95 | 50.20 | IGT |
| P37 | f | 62 | 38.2 | 121 | 62 | 5.9 | 386.72 | 25.98 | IGT |
| P38 | m | 77 | 26.5 | 77 | 18 | 5.9 | 331.92 | 34.47 | IGT |
| P39 | f | 49 | 25.9 | 93 | 39 | 5.8 | 494.29 | 43.56 | IGT |
| P40 | f | 56 | 19.8 | 117 | 24 | 6.2 | 449.74 | 56.74 | IGT |
| P41 | m | 51 | 24.7 | 110 | 54 | 5.6 | 358.99 | 66.58 | IFG |
| P42 | m | 58 | 29.4 | 102 | 97 | 5.8 | 808.88 | 48.03 | IGT |
| P43 | f | 76 | 32.9 | 124 | 135 | 5.6 | 466.33 | 72.39 | IFG |
| P44 | m | 79 | 25.7 | 81 | 13 | 6.3 | 298.61 | 58.30 | IGT |
| P45 | f | 66 | 20.1 | 112 | 30 | 6.1 | 471.18 | 68.80 | IGT |
| P46 | m | 76 | 24.8 | 93 | 42 | 5.9 | 415.99 | 61.00 | IGT |
| P47 | m | 87 | 25.8 | 106 | 43 | 5.2 | 212.48 | 57.49 | IFG |
| P48 | m | 51 | 18.5 | 102 | 8 | 5.3 | 75.16 | 60.71 | IFG |
| P49 | m | 75 | 29.3 | 101 | 150 | 5.4 | 755.95 | 61.23 | IFG |
| P50 | f | 62 | 24.2 | 102 | 38 | 6.0 | 310.47 | 51.66 | IGT |
| P51 | f | 63 | 21.6 | 109 | 52 | 6.1 | 443.02 | 67.05 | IGT |
| P52 | f | 75 | 24.5 | 81 | 82 | 6.0 | 473.62 | 54.26 | IGT |
| P53 | m | 67 | 28.7 | 94 | 148 | 6.0 | 347.16 | 52.81 | IGT |
| P54 | f | 74 | 22.1 | 116 | 67 | 5.9 | 626.16 | 47.77 | IGT |
| P55 | m | 49 | 21.4 | 116 | 108 | 6.7 | 323.64 | 61.10 | T2DM |

| | | | | | | | | | |
|---|---|---|---|---|---|---|---|---|---|
| P56 | f | 61 | 25.2 | 107 | 22 | 6.5 | 310.19 | 44.97 | T2DM |
| P57 | m | 78 | 21.0 | 115 | 29 | 4.6 | 633.13 | 35.48 | T2DM |
| P58 | m | 69 | 30.3 | 362 | 76 | 9.4 | 592.89 | 56.38 | T2DM |
| P59 | m | 64 | 42.4 | 156 | 271 | 6.6 | 593.10 | 64.35 | T2DM |
| P60 | f | 59 | 23.6 | 126 | 73 | 6.9 | 523.92 | 55.86 | T2DM |
| P61 | f | 70 | --- | 366 | 1056 | 12.6 | 358.38 | 61.78 | T2DM |
| P62 | m | 75 | 22.6 | 135 | 97 | 6.0 | 376.34 | 58.29 | T2DM |
| P63 | f | 45 | 24.5 | 142 | 77 | 7.0 | 740.06 | 63.54 | T2DM |
| P64 | m | 64 | 23.7 | 179 | 91 | 8.5 | 447.64 | 62.34 | T2DM |
| P65 | f | 77 | 18.8 | 148 | 32 | 6.8 | 411.49 | 62.91 | T2DM |
| P66 | m | 79 | 22.3 | 92 | 19 | 6.5 | 330.87 | 59.39 | T2DM |
| P67 | m | 55 | 23.0 | 111 | 59 | 6.9 | 582.79 | 57.08 | T2DM |
| P68 | f | 75 | 24.0 | 163 | 110 | 7.9 | 484.98 | 58.65 | T2DM |
| P69 | f | 70 | 26.6 | 181 | 42 | 7.3 | 450.59 | 61.92 | T2DM |
| P70 | m | 63 | 25.3 | 128 | 93 | 5.6 | 286.24 | 70.55 | T2DM |
| P71 | f | 76 | 29.6 | 98 | 92 | 6.8 | 367.59 | 52.92 | T2DM |
| P72 | f | 71 | 28.0 | 124 | 35 | 7.4 | 653.89 | 35.20 | T2DM |
| P73 | m | 75 | 23.2 | --- | --- | 7.2 | 593.81 | 46.03 | T2DM |
| P74 | m | 76 | 24.0 | 130 | 53 | 5.2 | 862.65 | 67.20 | T2DM |
| P75 | f | 75 | 23.8 | 144 | 59 | 8.0 | 384.81 | 54.34 | T2DM |
| P76 | m | 66 | 26.1 | 105 | 127 | 7.9 | 655.35 | 48.68 | T2DM |
| P77 | m | 77 | 31.1 | 136 | 55 | 6.3 | 525.58 | 45.95 | T2DM |
| P78 | m | 79 | 28.1 | --- | 58 | 8.3 | 349.38 | 56.28 | T2DM |

ESM Table 4: DEGs in NICCs follwing in vitro maturation

| Gene | Base Mean | log2FC | p adjusted | Gene | Base Mean | log2FC | p adjusted | Gene | Base Mean | log2FC | p adjusted |
|---|---|---|---|---|---|---|---|---|---|---|---|
| GCG | 277078 | 2.05 | 0.000210743 | BAALC | 560 | 1.00 | 0.00888218 | ADGRV1 | 147 | 1.77 | 9.55E-09 |
| CHGA | 83091 | 2.75 | 7.50E-10 | SLC7A14 | 556 | 1.46 | 0.00478387 | NEURL1B | 144 | 1.03 | 0.047226219 |
| INS | 43067 | 1.61 | 0.032114945 | KCNH6 | 551 | 1.36 | 0.0059594 | SULT4A1 | 144 | 1.04 | 4.09E-06 |
| SST | 36849 | 1.71 | 1.01E-06 | IRX2 | 540 | 1.41 | 0.00036019 | B3GALT5 | 143 | 1.63 | 4.90E-15 |
| CFD | 21617 | 1.14 | 0.002950341 | CLDN2 | 538 | -2.36 | 9.12E-05 | SNAP91 | 142 | 1.22 | 3.33E-05 |
| COL1A1 | 21532 | -1.53 | 0.015525605 | FBXL16 | 530 | 1.02 | 1.22E-06 | STEAP2 | 141 | 1.35 | 0.000158284 |
| RPS12 | 20939 | -1.00 | 1.57E-14 | MAPK8IP2 | 527 | 1.32 | 6.33E-05 | HCN4 | 137 | 1.71 | 4.00E-05 |
| CHGB | 20938 | 1.62 | 0.002966271 | PLEKHS1 | 523 | 2.68 | 0.00429352 | FAM184A | 137 | 1.62 | 1.14E-06 |
| PCSK1 | 14037 | 1.56 | 0.027312825 | NRCAM | 523 | 1.29 | 1.34E-12 | RCVRN | 136 | 1.30 | 0.001121921 |
| CPE | 13424 | 2.49 | 2.84E-08 | SLC25A53 | 518 | 1.13 | 0.03973873 | CBLN4 | 136 | 2.65 | 0.000355491 |
| CRTAC1 | 12909 | 1.47 | 9.05E-06 | HSD17B13 | 511 | -1.05 | 0.00015562 | ST8SIA3 | 136 | 1.29 | 0.000852214 |
| YBX3 | 11917 | -1.03 | 6.21E-05 | HSD17B13 | 511 | -1.05 | 0.00015562 | CNTN4 | 132 | 1.39 | 0.000775174 |
| PCSK2 | 11191 | 2.11 | 5.61E-05 | ANK2 | 507 | 1.05 | 0.00526573 | LTC4S | 132 | -1.81 | 0.02462859 |
| PPY | 10557 | 1.75 | 0.000300393 | ST18 | 501 | 1.31 | 0.00301927 | PKHD1L1 | 132 | 1.79 | 5.74E-08 |
| S100A6 | 10491 | -1.21 | 1.46E-05 | MGAM | 500 | 1.45 | 0.0007209 | FAM3B | 131 | 1.07 | 0.001008665 |
| SCG2 | 9725 | 1.88 | 0.000967341 | RAB9B | 489 | 1.29 | 0.01000654 | ASTN2 | 131 | 1.03 | 0.011149458 |
| PAPPA2 | 8577 | 2.95 | 2.79E-06 | PLCG2 | 479 | 1.26 | 5.74E-08 | BEX5 | 130 | 1.48 | 0.001384154 |
| SCGN | 8275 | 1.68 | 0.000821926 | C3orf70 | 477 | 1.12 | 0.00036019 | IGFBPL1 | 130 | 1.66 | 0.001893839 |
| SCG5 | 8234 | 2.03 | 8.25E-10 | PAPLN | 468 | 1.49 | 3.30E-10 | REG4 | 129 | 2.52 | 0.043724759 |
| CLPS | 7440 | -3.77 | 1.87E-09 | SNAI2 | 466 | -1.57 | 2.62E-06 | TMEM151A | 127 | 1.06 | 0.00015436 |
| TIMP1 | 6503 | -1.77 | 7.43E-08 | GPX3 | 458 | 1.52 | 0.00527801 | TLCD2 | 126 | 1.03 | 9.25E-13 |
| VGF | 5959 | 1.40 | 0.003512179 | ANG | 458 | -1.80 | 0.02505642 | GRIK4 | 126 | 1.46 | 0.00322882 |
| BTG2 | 5771 | -1.57 | 1.62E-13 | PNLIPRP1 | 458 | -1.65 | 0.00100117 | ABHD15 | 125 | 1.46 | 0.000140924 |
| PRSS2 | 5768 | 3.62 | 0.000601572 | ASB9 | 457 | 1.28 | 0.03924073 | LEPR | 124 | 1.90 | 1.49E-06 |
| SLC7A8 | 5724 | 1.09 | 0.005982663 | CRMP1 | 455 | 1.10 | 1.15E-05 | NEUROG3 | 124 | 1.10 | 0.000586464 |
| MMP9 | 5434 | 1.97 | 4.80E-13 | RBP4 | 450 | 1.60 | 0.00067418 | BMP3 | 123 | 1.12 | 0.000240169 |
| PDCD4 | 5365 | -1.08 | 0.014699279 | GLYCTK | 450 | 1.07 | 1.00E-06 | FAS | 123 | -1.09 | 1.63E-07 |
| SLC7A2 | 5273 | 1.35 | 0.002982104 | PKNOX2 | 446 | -1.37 | 0.00028101 | CCDC60 | 123 | 1.58 | 0.000597552 |
| HEPACAM2 | 5190 | 1.55 | 0.004663801 | ACE | 440 | 1.47 | 1.09E-10 | CCDC60 | 123 | 1.58 | 0.000597552 |
| APOA1 | 4856 | 1.44 | 5.74E-08 | SEL1L3 | 437 | 1.14 | 0.0001867 | AATK | 123 | 1.58 | 0.000210743 |
| ANKRD1 | 4729 | 1.59 | 0.017244845 | RNASE4 | 427 | -1.18 | 0.03114847 | ASPN | 122 | -1.85 | 0.031811515 |
| TNC | 4663 | -2.25 | 0.000568952 | PPP1R1A | 420 | 2.16 | 7.78E-08 | SLC24A2 | 120 | 3.19 | 0.000597552 |
| GRIA3 | 4295 | 1.79 | 6.38E-07 | TSPAN11 | 416 | 1.35 | 0.00071252 | DPYSL4 | 120 | 1.06 | 1.11E-06 |
| TTR | 4028 | 1.76 | 6.72E-05 | RADX | 413 | 1.52 | 0.00010081 | ACTG2 | 118 | -2.36 | 0.001589912 |
| CPLX2 | 3963 | 1.29 | 0.000160145 | SCN3A | 411 | 1.28 | 1.84E-05 | ADGRB3 | 117 | 1.13 | 0.001116713 |
| CACNA2D1 | 3945 | 1.26 | 9.61E-06 | MMP17 | 411 | 2.08 | 2.73E-05 | PRODH2 | 116 | 2.22 | 3.72E-12 |
| TF | 3886 | 4.06 | 3.97E-14 | CACNA1C | 406 | 1.06 | 0.00544477 | C1QB | 116 | 1.54 | 0.000817443 |
| PTPRN | 3830 | 1.81 | 7.77E-06 | C4BPA | 399 | 1.39 | 0.00012901 | HPX | 115 | 2.14 | 1.20E-07 |
| CNTN1 | 3702 | 2.04 | 4.05E-06 | C4BPA | 399 | 1.39 | 0.00012901 | USP35 | 115 | 1.02 | 5.68E-07 |
| SCG3 | 3684 | 1.40 | 3.68E-05 | SLC4A10 | 393 | 1.59 | 0.00073339 | FRRS1L | 115 | 1.80 | 3.43E-05 |
| LRRN3 | 3662 | 1.57 | 0.024988661 | RPS6KL1 | 393 | 1.15 | 3.80E-26 | TRPC5 | 115 | 1.16 | 0.018587034 |
| CBX6 | 3646 | 1.01 | 0.000415972 | CERKL | 392 | 1.37 | 0.00078517 | ASTN1 | 114 | 1.19 | 5.17E-05 |
| ABCC8 | 3418 | 1.78 | 0.001838096 | NELL2 | 391 | 1.38 | 0.00087423 | ZNF536 | 113 | 1.07 | 0.026207544 |
| BHLHE40 | 3357 | -1.16 | 1.14E-07 | PBK | 387 | 1.31 | 0.02646044 | NMB | 113 | -1.98 | 1.88E-06 |
| PTPRN2 | 3355 | 2.07 | 5.09E-07 | SLC4A8 | 384 | 1.46 | 1.40E-06 | GLP1R | 113 | 1.01 | 0.001939944 |
| TSC22D3 | 3285 | -1.10 | 0.009104797 | NFASC | 382 | 1.75 | 5.91E-08 | DAB1 | 112 | 1.38 | 1.79E-05 |
| LBH | 3248 | -1.04 | 0.002041128 | SEMA3E | 378 | 1.23 | 2.51E-07 | RF00002 | 111 | -1.30 | 4.24E-06 |

| | | | | | | | | | | | |
|---|---|---|---|---|---|---|---|---|---|---|---|
| GPNMB | 3227 | 1.50 | 3.05E-07 | MGAT3 | 377 | 1.08 | 2.37E-06 | TFF3 | 110 | 1.81 | 0.035340147 |
| DDIT4 | 3111 | -1.47 | 9.47E-18 | CAMK2B | 376 | 1.19 | 0.00038109 | FXYD1 | 110 | 1.37 | 9.54E-05 |
| C14orf132 | 3099 | 1.03 | 6.04E-05 | ITGA4 | 376 | 1.44 | 0.00369191 | CAPN6 | 110 | 2.48 | 0.031299357 |
| MT1A | 3004 | -2.72 | 0.001220079 | SLC25A34 | 375 | 1.11 | 1.14E-07 | ZNF483 | 109 | 1.02 | 1.56E-09 |
| MT1A | 3004 | -2.72 | 0.001220079 | PLCL2 | 373 | 1.36 | 0.00722632 | THSD4 | 109 | 1.44 | 1.86E-07 |
| ABCA1 | 2999 | 1.64 | 3.95E-18 | TNR | 371 | 2.56 | 1.53E-15 | RNF152 | 109 | 1.05 | 0.000574929 |
| PAX6 | 2938 | 1.24 | 0.003781555 | CABP7 | 368 | 1.84 | 5.17E-10 | SRPX2 | 108 | -1.36 | 0.046174532 |
| ESM1 | 2916 | 1.63 | 0.001608018 | ADM | 366 | -1.36 | 0.00470981 | AQP5 | 108 | -1.26 | 0.002835895 |
| NBEA | 2705 | 1.19 | 8.67E-08 | SPOCK3 | 361 | 1.13 | 1.60E-06 | GRIN3A | 107 | 1.82 | 7.24E-05 |
| SEMA3B | 2648 | 1.11 | 0.034303955 | EML6 | 360 | 1.98 | 2.60E-05 | COL4A4 | 107 | 1.63 | 0.020709488 |
| KIAA1324 | 2567 | 1.05 | 0.046002021 | EFNB3 | 359 | 1.42 | 2.22E-09 | MSR1 | 106 | 1.38 | 0.022265735 |
| KLF10 | 2556 | -1.00 | 0.000327298 | CDKN1C | 358 | -1.06 | 0.00094217 | YBX2 | 106 | -1.14 | 0.005416335 |
| MAPT | 2446 | 1.26 | 1.30E-05 | PDE1C | 354 | 1.01 | 0.00384597 | ISG20 | 106 | -1.69 | 2.21E-11 |
| SYT13 | 2432 | 1.61 | 0.001407124 | UCN3 | 351 | 2.13 | 0.0006727 | DCDC1 | 106 | 1.08 | 0.008223956 |
| SLC38A4 | 2415 | 1.85 | 1.12E-07 | PEX5L | 350 | 1.96 | 1.23E-05 | KCNC2 | 105 | 2.56 | 3.10E-07 |
| P4HA1 | 2399 | -1.05 | 8.55E-09 | SLC16A3 | 348 | -1.08 | 0.0303094 | ACSBG1 | 104 | 1.11 | 9.49E-06 |
| ABCG1 | 2393 | 1.84 | 1.32E-17 | PYY | 340 | 1.84 | 0.00384693 | KIAA0408 | 104 | 1.06 | 6.99E-07 |
| APLP1 | 2385 | 1.45 | 1.27E-08 | RYR2 | 340 | 1.62 | 0.00058985 | DPEP2 | 104 | 1.83 | 0.00227507 |
| PLA2G1B | 2335 | -4.40 | 6.79E-07 | DPP10 | 338 | 1.82 | 7.32E-05 | CPNE4 | 104 | 1.45 | 4.23E-06 |
| GPR155 | 2329 | -1.95 | 2.10E-06 | GPRC5A | 338 | -1.44 | 2.81E-08 | PLB1 | 104 | 1.62 | 0.002195334 |
| GNAO1 | 2265 | 1.32 | 0.000423906 | SYT17 | 336 | 1.02 | 0.00595851 | LOXL1 | 103 | -1.63 | 0.02054069 |
| POSTN | 2253 | -1.66 | 0.009506177 | SEZ6 | 335 | 1.65 | 0.00010062 | E2F2 | 103 | 1.31 | 0.014392839 |
| SPOCK2 | 2224 | 1.29 | 9.14E-13 | TFF2 | 335 | -2.10 | 0.02777322 | LRCH2 | 103 | 1.03 | 0.006241905 |
| CP | 2042 | 2.08 | 3.27E-06 | GPR63 | 321 | -1.27 | 2.37E-05 | ZFYVE28 | 103 | 1.04 | 5.96E-08 |
| RFX6 | 1935 | 1.73 | 4.33E-08 | SCIN | 317 | 1.20 | 0.03770471 | PTGFR | 103 | 1.51 | 0.00027236 |
| PCSK1N | 1918 | 1.19 | 0.002851175 | ANXA13 | 317 | -1.23 | 0.00074333 | DHH | 102 | 1.06 | 0.008525261 |
| CELF3 | 1874 | 1.52 | 1.39E-07 | PRKAR2B | 315 | 1.08 | 1.20E-07 | ARC | 102 | -1.04 | 0.009319151 |
| ABAT | 1850 | 1.24 | 0.003597621 | RARRES1 | 309 | 1.27 | 2.52E-06 | GPR119 | 102 | 2.19 | 0.000402586 |
| ZFP36 | 1833 | -1.00 | 1.84E-07 | CISH | 309 | -1.00 | 1.24E-07 | SPRED3 | 102 | 1.29 | 0.000668792 |
| RAB26 | 1827 | 1.20 | 0.001415 | GPER1 | 308 | -1.20 | 8.78E-07 | KNDC1 | 102 | 1.40 | 4.90E-07 |
| CACNA1H | 1779 | 1.13 | 8.74E-08 | CFAP74 | 305 | 1.48 | 5.11E-08 | NCF2 | 100 | 1.34 | 0.003092975 |
| SYT4 | 1759 | 1.72 | 5.21E-05 | SYBU | 301 | 1.10 | 2.56E-06 | KIAA0319 | 100 | 1.47 | 8.56E-08 |
| SCD | 1735 | 1.32 | 3.71E-13 | PLXDC2 | 300 | 1.16 | 1.98E-05 | FGL2 | 99 | 1.05 | 0.001082771 |
| GCA | 1653 | -1.37 | 0.000255357 | PTGER3 | 295 | 1.06 | 0.00121363 | C2orf88 | 99 | 1.31 | 0.00465216 |
| C6orf222 | 1622 | -1.00 | 0.025139127 | BSN | 294 | 1.34 | 1.22E-05 | RELN | 99 | 1.55 | 0.004595525 |
| ATF5 | 1568 | 1.11 | 2.92E-05 | OVOL1 | 291 | -1.00 | 8.73E-05 | CHRM4 | 99 | 1.04 | 6.90E-10 |
| L1CAM | 1560 | 1.53 | 1.09E-05 | NALCN | 283 | 1.48 | 0.00096486 | THBS3 | 98 | -1.02 | 2.37E-06 |
| SFRP5 | 1550 | 1.11 | 0.036672417 | SDF2L1 | 283 | 1.32 | 0.00357853 | LRFN1 | 98 | 1.01 | 0.000116986 |
| PFKFB2 | 1544 | 1.12 | 0.000216265 | FFAR3 | 282 | 1.62 | 0.00017969 | DNAH2 | 97 | 1.24 | 2.69E-07 |
| MLXIPL | 1538 | 1.63 | 4.97E-06 | ADGRB2 | 272 | 1.30 | 8.54E-06 | GALP | 97 | 1.58 | 0.027773217 |
| BDKRB2 | 1535 | 1.35 | 0.013714852 | CDK5R2 | 269 | 1.58 | 4.27E-05 | NPY | 96 | -1.07 | 0.031762907 |
| SEZ6L2 | 1533 | 1.95 | 1.23E-05 | TMEM130 | 267 | 1.18 | 0.00509481 | APOD | 94 | 1.25 | 0.019879981 |
| BNIP3 | 1523 | -1.30 | 2.02E-07 | ETV5 | 264 | 1.25 | 2.87E-06 | GEN1 | 94 | 1.05 | 0.027478448 |
| NEUROD1 | 1502 | 1.60 | 0.000260308 | GRM4 | 264 | 1.08 | 0.02799577 | LYPD8 | 94 | 2.57 | 0.003553897 |
| EPB41L3 | 1451 | 1.01 | 1.08E-05 | COL17A1 | 264 | -1.11 | 5.64E-10 | NYAP2 | 94 | 1.08 | 0.020719956 |
| KNG1 | 1431 | 2.25 | 0.00595851 | ARG2 | 263 | 1.14 | 0.00049588 | MAP3K15 | 94 | 1.72 | 2.47E-05 |
| SYP | 1430 | 1.26 | 0.000300146 | E2F7 | 260 | 1.14 | 0.00469376 | SCARA5 | 94 | 3.35 | 7.97E-12 |
| BAIAP3 | 1417 | 1.28 | 8.03E-06 | LYPD6 | 259 | 1.26 | 4.25E-05 | TMEM229B | 93 | 1.21 | 4.05E-12 |
| KIF5C | 1355 | 1.31 | 1.05E-13 | PSD3 | 258 | 1.05 | 7.10E-06 | DISP2 | 93 | 1.35 | 0.000321622 |

| | | | | | | | | | | | |
|---|---|---|---|---|---|---|---|---|---|---|---|
| INSM1 | 1350 | 1.47 | 0.000687074 | ITGB2 | 258 | 1.41 | 6.63E-05 | A1CF | 93 | 1.10 | 8.85E-06 |
| CNTN3 | 1349 | 1.49 | 8.35E-05 | GAL | 258 | 1.52 | 0.00047739 | PRKG2 | 92 | 1.00 | 0.000494842 |
| SORL1 | 1339 | 1.53 | 4.52E-05 | LGI4 | 254 | 1.06 | 0.00034762 | LRRN1 | 91 | 1.49 | 0.000850859 |
| ANKH | 1328 | 1.15 | 2.51E-05 | S100A13 | 253 | -1.08 | 4.24E-05 | C4BPB | 89 | 1.61 | 0.00181821 |
| PLCXD3 | 1304 | 1.91 | 0.000711893 | SHCBP1 | 250 | 1.13 | 0.0460372 | KCNF1 | 89 | 2.09 | 2.23E-05 |
| PI15 | 1295 | -1.06 | 0.01411393 | SEMA5A | 245 | 1.49 | 0.00113713 | IL1A | 88 | 2.37 | 0.03327108 |
| MAN1A1 | 1287 | 1.01 | 6.39E-12 | EML5 | 245 | 1.27 | 1.76E-05 | BEGAIN | 88 | 1.89 | 0.001384792 |
| MYO3A | 1284 | 2.02 | 0.005118268 | PRND | 244 | 3.83 | 9.97E-05 | GABBR2 | 88 | 1.25 | 0.004874993 |
| LRP11 | 1284 | 1.01 | 0.002564816 | CHRNB2 | 240 | 1.30 | 0.00029253 | CPA2 | 86 | -2.33 | 0.00474465 |
| DNER | 1278 | 1.33 | 0.000122857 | MYT1 | 237 | 1.74 | 6.65E-07 | C20orf96 | 86 | 1.47 | 0.000748991 |
| RAB3B | 1270 | 1.66 | 1.71E-05 | HMGCLL1 | 236 | 1.44 | 0.00141793 | RUNX3 | 86 | 1.17 | 0.031762907 |
| RF01957 | 1256 | 1.01 | 0.001533864 | SVIP | 233 | 1.13 | 1.66E-10 | LINGO1 | 84 | 1.21 | 7.92E-05 |
| OLFM1 | 1250 | 1.22 | 0.000381295 | AURKB | 233 | 1.03 | 0.00790766 | PRRX2 | 84 | -1.41 | 0.012810302 |
| PGAP1 | 1245 | 1.24 | 4.16E-12 | SLC35G2 | 231 | 1.27 | 0.00410937 | DPP6 | 84 | 1.91 | 2.03E-11 |
| VWF | 1219 | 1.45 | 0.001463951 | GABRB3 | 230 | 1.78 | 3.68E-06 | SLC2A5 | 84 | -2.69 | 0.000128839 |
| RF01956 | 1209 | 1.00 | 0.001848365 | C1QC | 226 | 1.59 | 0.00038811 | FGF10 | 83 | 1.06 | 0.000596194 |
| ANXA10 | 1189 | 1.28 | 0.005310652 | DNAH9 | 226 | 1.04 | 0.01301852 | SLC18A3 | 83 | 1.43 | 0.004925154 |
| OPCML | 1179 | 1.55 | 0.030064295 | TRABD2B | 223 | -2.14 | 1.54E-08 | FSTL5 | 82 | 1.50 | 4.04E-05 |
| NNAT | 1171 | 1.18 | 0.001737584 | SERPIND1 | 223 | 1.80 | 0.00011392 | GPR75 | 82 | 1.02 | 0.000147712 |
| GJD2 | 1153 | 1.92 | 0.004104282 | CILP | 221 | -2.38 | 0.03486529 | SYN2 | 81 | 1.21 | 6.73E-05 |
| OLFM4 | 1133 | 1.86 | 0.03490004 | FOLR1 | 221 | 1.47 | 7.38E-05 | ATP1B2 | 81 | 1.03 | 3.23E-05 |
| SCN7A | 1101 | 2.36 | 5.85E-07 | FOLR1 | 221 | 1.47 | 7.38E-05 | CYTL1 | 81 | 1.58 | 0.048012805 |
| CELSR3 | 1070 | 1.19 | 1.79E-06 | BPIFA1 | 219 | 3.08 | 3.37E-09 | CLCA1 | 80 | 1.97 | 0.032855565 |
| AKAP12 | 1057 | 1.09 | 1.16E-06 | IL17D | 217 | -1.01 | 2.08E-12 | GHSR | 80 | 1.35 | 0.005496077 |
| MPZ | 1044 | 1.73 | 2.88E-06 | IGFBP1 | 217 | 1.35 | 0.03740481 | RASGRF2 | 80 | 1.14 | 0.001458024 |
| LIMCH1 | 1024 | 1.16 | 4.95E-05 | HEYL | 216 | -1.45 | 0.02668806 | EXTL1 | 80 | 1.18 | 0.004476919 |
| SYT7 | 1018 | 1.37 | 0.000337864 | KCNQ2 | 215 | 1.33 | 7.76E-10 | PTPRC | 79 | 1.42 | 0.002875498 |
| ARFGEF3 | 1001 | 1.15 | 4.38E-07 | MED12L | 214 | 1.34 | 1.47E-07 | PPP1R1B | 78 | -1.14 | 0.001691595 |
| PLXNB3 | 999 | 1.35 | 0.001021019 | TMPRSS11F | 213 | 2.28 | 0.00072068 | BRINP1 | 78 | 1.92 | 1.23E-05 |
| ITIH5 | 998 | 1.38 | 0.003693384 | TARSL2 | 211 | -1.11 | 4.41E-05 | ARPP21 | 78 | 1.34 | 0.016148675 |
| METTL21A | 993 | -1.21 | 3.38E-15 | CACNB2 | 210 | 1.05 | 0.00771981 | PCP4 | 78 | 2.04 | 0.007877112 |
| ATP2A3 | 980 | 1.08 | 0.011637524 | FAM162B | 210 | 1.30 | 0.00022402 | ADAM11 | 77 | 1.47 | 7.36E-05 |
| GCH1 | 954 | 1.12 | 0.000443629 | FOXD3 | 207 | 1.00 | 0.04261734 | KCNIP1 | 76 | 1.84 | 0.000646638 |
| ADCY1 | 951 | 1.99 | 2.79E-08 | CENPA | 206 | 1.30 | 0.03165148 | DIRAS2 | 76 | 1.11 | 0.020841174 |
| TENM3 | 947 | 1.05 | 0.006627354 | KCNA4 | 204 | 2.03 | 0.00525411 | SYT14 | 76 | 1.61 | 2.85E-05 |
| VWA5B2 | 915 | 1.79 | 2.45E-06 | GALNT13 | 204 | 1.56 | 0.00011779 | RAB7B | 75 | 1.87 | 0.024109416 |
| CACNA1A | 913 | 1.39 | 0.000239361 | AMPH | 200 | 1.39 | 0.00026338 | CNN1 | 75 | -1.37 | 0.037032843 |
| SHISAL1 | 906 | 1.47 | 8.18E-07 | DISP3 | 199 | 1.39 | 0.0007406 | PCDH10 | 74 | 1.10 | 0.000685641 |
| SYT5 | 896 | 1.02 | 0.038458912 | SCEL | 199 | 1.62 | 3.81E-05 | DOK6 | 74 | 1.52 | 0.003070209 |
| MMP13 | 894 | 1.95 | 0.004819238 | TENM4 | 199 | 1.14 | 0.00249608 | MYRIP | 74 | 1.22 | 2.11E-06 |
| SYT11 | 886 | 1.00 | 0.000203752 | DOCK11 | 198 | 1.02 | 0.00040362 | CLEC2B | 73 | -1.75 | 2.19E-05 |
| SGSM1 | 886 | 1.15 | 1.29E-11 | KCNJ5 | 198 | 1.30 | 0.00930945 | PNCK | 73 | 1.18 | 2.40E-08 |
| SCN9A | 877 | 1.29 | 1.81E-10 | MAPK4 | 197 | 1.14 | 1.16E-10 | C5AR1 | 73 | 1.25 | 0.011915123 |
| RGS6 | 873 | 1.12 | 0.001428865 | INSC | 195 | 1.29 | 0.00599134 | RD3 | 72 | 1.56 | 0.004467751 |
| TFR2 | 870 | 1.65 | 1.45E-17 | SNAI1 | 195 | -1.14 | 0.01722915 | DIO2 | 72 | 1.89 | 0.004517532 |
| CHL1 | 869 | 1.26 | 0.000192933 | DAAM2 | 194 | 1.33 | 1.16E-05 | ADAMTS20 | 71 | 1.89 | 0.021111122 |
| PDE3B | 853 | 1.24 | 3.15E-07 | RFX2 | 194 | 1.12 | 0.00075801 | ST6GALNAC | 71 | 1.60 | 1.09E-05 |
| RIMBP2 | 841 | 1.49 | 4.05E-06 | C11orf87 | 194 | 1.72 | 7.97E-06 | KCNG3 | 70 | 2.02 | 0.002997038 |
| CASR | 826 | 1.68 | 0.000132164 | PRLR | 192 | 1.44 | 0.00926864 | SSTR2 | 70 | 1.54 | 0.00499625 |

| | | | | | | | | | | | |
|---|---|---|---|---|---|---|---|---|---|---|---|
| HLF | 820 | 1.01 | 5.08E-05 | ATP8A1 | 191 | 1.28 | 4.10E-09 | MYT1L | 70 | 1.73 | 3.34E-05 |
| TGFB3 | 820 | -1.28 | 6.21E-11 | OGDHL | 191 | 1.62 | 8.90E-06 | ADAM22 | 70 | 1.07 | 0.043957662 |
| KCNK16 | 806 | 1.62 | 0.019310502 | CFAP100 | 191 | 1.21 | 1.20E-08 | HSD11B2 | 70 | -1.13 | 0.007961975 |
| ARHGEF9 | 803 | 1.12 | 0.0136123 | TMEM132B | 190 | 1.62 | 0.00618494 | MAP7D2 | 70 | 1.36 | 0.000190757 |
| SOWAHA | 802 | 1.16 | 0.002102483 | ADCK1 | 190 | 1.05 | 7.60E-09 | KCNJ6 | 69 | 1.28 | 0.001315363 |
| VCAM1 | 790 | -1.19 | 0.000105868 | CPA5 | 190 | -2.96 | 5.26E-05 | PHF24 | 69 | 1.10 | 0.001609276 |
| TMEM59L | 787 | 1.66 | 3.31E-11 | CPA5 | 190 | -2.96 | 5.26E-05 | IL6 | 69 | 2.23 | 0.037432922 |
| SLC29A4 | 787 | 1.49 | 5.36E-13 | DSCAML1 | 189 | 1.50 | 4.35E-09 | BTBD17 | 69 | 2.35 | 0.000663225 |
| RF00100 | 778 | -1.79 | 2.39E-17 | SCUBE3 | 189 | 1.45 | 0.01027572 | ADAMTS14 | 68 | 1.26 | 0.002570643 |
| SNAP25 | 777 | 1.40 | 0.000555727 | NEGR1 | 189 | 1.21 | 0.00031946 | STK33 | 68 | 1.08 | 0.001010253 |
| GADD45B | 767 | -1.04 | 0.005452667 | AREG | 189 | 1.34 | 0.04841124 | SLC9A2 | 68 | 1.12 | 0.00062684 |
| GRIK1 | 744 | 1.99 | 4.70E-05 | CENPT | 187 | 1.09 | 0.04460668 | GIPR | 68 | 1.79 | 7.58E-05 |
| KCTD12 | 743 | 1.13 | 0.009518927 | ARHGAP25 | 186 | 1.00 | 0.00271269 | C1QL1 | 68 | 1.27 | 0.025749738 |
| UPP1 | 736 | -1.58 | 9.54E-13 | CELF4 | 184 | 1.33 | 9.61E-09 | PRLHR | 67 | 1.50 | 0.000951339 |
| RET | 732 | 1.16 | 1.16E-05 | C1QA | 183 | 1.64 | 0.00011511 | HHIPL2 | 67 | 1.86 | 0.00465216 |
| NOL4 | 729 | 1.45 | 0.000297892 | CKAP2L | 181 | 1.11 | 0.01677571 | B3GALT1 | 67 | 1.33 | 3.35E-10 |
| PIP5K1B | 728 | 1.29 | 3.53E-10 | TMEM163 | 180 | 1.40 | 2.17E-06 | CASKIN1 | 67 | 1.07 | 3.66E-05 |
| ISL1 | 708 | 1.42 | 0.000993613 | FRMD5 | 180 | 1.18 | 9.96E-05 | PIK3CG | 66 | 1.03 | 0.011618415 |
| SDK1 | 671 | 1.13 | 5.67E-21 | SYNDIG1L | 180 | 1.48 | 0.01447735 | COL13A1 | 66 | 1.10 | 2.01E-05 |
| CADM3 | 671 | 1.80 | 1.18E-07 | GPR158 | 179 | 1.15 | 0.00988449 | ATP8A2 | 65 | 1.05 | 0.002547703 |
| ADAMTS17 | 668 | 1.12 | 1.19E-06 | CALB1 | 178 | 1.58 | 0.03290682 | LYVE1 | 65 | 1.82 | 0.009230087 |
| FRMD3 | 660 | 1.09 | 9.95E-09 | APOH | 178 | 1.12 | 0.04333916 | SHISA7 | 65 | 1.32 | 0.000261586 |
| TCN1 | 646 | 1.76 | 0.004448106 | SLCO4A1 | 178 | 1.09 | 0.0018741 | PHF21B | 64 | 1.03 | 0.000170858 |
| CLSTN3 | 643 | 1.20 | 0.001678968 | CORIN | 176 | -2.06 | 0.006229 | MYO7B | 64 | 1.61 | 0.010077131 |
| SLC30A8 | 638 | 1.47 | 0.039553389 | IQSEC3 | 175 | 1.09 | 0.0051513 | ARHGAP30 | 62 | 1.11 | 0.007544152 |
| TMOD2 | 632 | 1.04 | 6.77E-05 | RIMS2 | 175 | 1.03 | 1.61E-06 | SHAS2 | 62 | -1.56 | 0.04023123 |
| GALNT18 | 626 | 1.05 | 0.003405027 | HAP1 | 174 | 1.28 | 8.50E-07 | REC8 | 62 | 1.04 | 0.002988398 |
| RENBP | 622 | 1.09 | 0.000509206 | MATN4 | 174 | -1.67 | 0.0157833 | OTOP3 | 61 | 1.00 | 0.025416614 |
| LSAMP | 622 | 1.97 | 3.24E-06 | GABRG2 | 174 | 1.56 | 7.08E-22 | MAMDC2 | 61 | 1.14 | 0.007613573 |
| DPP4 | 620 | 1.65 | 3.82E-05 | ELAVL4 | 173 | 1.04 | 7.10E-08 | FCER1G | 61 | 1.43 | 0.001981893 |
| SYNGAP1 | 617 | 1.14 | 7.39E-07 | ACVR1C | 171 | 1.57 | 0.01030005 | DNAH5 | 61 | 1.46 | 5.75E-07 |
| CHIA | 613 | 2.10 | 6.64E-21 | SRCIN1 | 170 | 1.70 | 0.00021018 | IRX1 | 61 | 1.65 | 0.000186426 |
| GDF15 | 613 | 1.58 | 0.002474495 | ARX | 170 | 1.08 | 0.01241824 | RF00045 | 60 | -1.09 | 0.002227552 |
| P2RY14 | 610 | 1.49 | 0.00291304 | TROAP | 169 | 1.29 | 0.01000706 | CLVS2 | 60 | 1.36 | 0.008662699 |
| TANC2 | 608 | 1.07 | 0.000132809 | PLPPR4 | 168 | 1.48 | 0.00276806 | B3GALT2 | 60 | 1.69 | 0.002055995 |
| CDH19 | 608 | 1.26 | 0.003847341 | DCX | 168 | 1.16 | 0.0156908 | SLC5A7 | 60 | 1.30 | 0.000330563 |
| CHST9 | 603 | 2.15 | 3.20E-07 | GC | 167 | 2.35 | 7.30E-31 | GBA3 | 59 | 2.57 | 1.33E-05 |
| ROBO2 | 602 | 1.29 | 0.015094277 | SULT1C4 | 165 | 1.41 | 0.01774942 | FREM1 | 59 | 1.81 | 0.005447663 |
| MXRA8 | 600 | -1.14 | 0.001570737 | LSP1 | 164 | -1.35 | 0.01781204 | IRX4 | 59 | -1.56 | 0.000508287 |
| FHOD3 | 596 | 1.48 | 1.15E-05 | SLITRK6 | 162 | 1.55 | 0.02056965 | SVOP | 58 | 1.44 | 0.000396107 |
| FHL3 | 593 | -1.03 | 2.12E-10 | GKN3 | 161 | 2.50 | 5.78E-06 | PADI1 | 57 | 1.62 | 0.039240735 |
| ITIH4 | 591 | 1.39 | 0.003832495 | HYKK | 161 | -1.19 | 0.00061925 | UNC5D | 57 | 1.31 | 8.95E-05 |
| ANXA9 | 587 | 1.08 | 4.38E-07 | KIF19 | 161 | 1.00 | 0.00062028 | CMAH | 57 | 2.09 | 8.56E-05 |
| PLCB4 | 586 | 1.24 | 1.36E-08 | MAST1 | 160 | 1.04 | 1.73E-05 | RAC2 | 57 | 1.04 | 0.040451238 |
| TRPA1 | 581 | 2.92 | 2.19E-05 | MALRD1 | 157 | 2.36 | 1.04E-06 | UNC45B | 57 | -1.15 | 0.017708824 |
| LACTB | 580 | -1.70 | 3.90E-10 | RAB39B | 157 | 1.12 | 6.99E-07 | WSCD2 | 56 | 1.35 | 0.046976153 |
| SPTBN4 | 579 | 1.14 | 0.000135299 | ATP1A2 | 157 | 1.47 | 0.00816475 | TMEM117 | 56 | 1.12 | 0.00517301 |
| ACTA2 | 577 | -1.31 | 0.042303101 | CCDC68 | 156 | 1.60 | 1.68E-06 | NCAM2 | 56 | 1.48 | 1.74E-05 |
| PCLAF | 577 | 1.35 | 0.031762907 | CCDC170 | 156 | 1.17 | 6.44E-05 | CD163 | 56 | 1.02 | 0.025744294 |

| | | | |
|---|---|---|---|
| UNC5A | 576 | 1.29 | 0.001407124 |
| S100A14 | 574 | -1.23 | 0.000599697 |
| PENK | 569 | 1.88 | 6.77E-05 |
| EGLN3 | 564 | -1.06 | 0.002469478 |
| STMN3 | 562 | 1.15 | 0.001446791 |
| UNC80 | 561 | 1.66 | 0.000300001 |
| PTF1A | 560 | -1.43 | 0.032880137 |
| CACNA1E | 156 | 1.59 | 1.72E-05 |
| NRXN1 | 152 | 2.11 | 1.39E-10 |
| MIOX | 151 | 1.05 | 0.00017792 |
| LRRN2 | 150 | 1.47 | 1.17E-05 |
| SNTG1 | 149 | 1.27 | 0.00710962 |
| SOX5 | 148 | 1.32 | 1.05E-06 |
| SSTR3 | 147 | 2.55 | 1.86E-09 |
| GRIK2 | 147 | 1.39 | 7.20E-09 |
| PNMA2 | 55 | 1.23 | 1.28E-05 |
| DPT | 55 | -1.87 | 5.11E-06 |
| VAV1 | 54 | 1.26 | 0.009144209 |
| GABRR2 | 54 | 1.44 | 0.037836379 |
| TMIE | 54 | 1.06 | 0.035561896 |
| SIDT1 | 53 | 1.41 | 0.031080008 |
| SYTL5 | 53 | -1.03 | 0.022428479 |
| NTM | 53 | 1.78 | 5.41E-05 |
| RAB39A | 53 | 1.24 | 0.001708472 |
| TGM1 | 51 | -2.54 | 8.64E-23 |
| PRR16 | 50 | 1.33 | 0.001262419 |

ESM Table 5: DEGs in NICCs isolated from 4 days old animals upon culture with fetuin-A

| Gene | Base Mean | log2FC | p adjusted | Gene | Base Mean | log2FC | p adjusted | Gene | Base Mean | log2FC | p adjusted |
|---|---|---|---|---|---|---|---|---|---|---|---|
| GCG | 277078 | -4.06 | 5.61E-33 | NEURL3 | 546 | 4.20 | 5.42E-07 | LSP1 | 164 | -2.34 | 0.000119046 |
| ATP1A1 | 113022 | 1.10 | 2.93E-06 | CPLX1 | 546 | 1.03 | 2.36E-05 | ESCO2 | 164 | -1.56 | 4.83E-09 |
| CHGA | 83091 | -2.16 | 6.05E-13 | IRX2 | 540 | -2.15 | 4.71E-10 | MKRN2OS | 163 | 1.23 | 2.79E-07 |
| CEL | 69792 | -2.94 | 0.011524988 | POLE | 536 | -2.48 | 7.56E-18 | GKN3 | 161 | -2.98 | 1.51E-07 |
| INS | 43067 | -2.23 | 0.0006457 | LOX | 528 | 1.00 | 0.042149294 | KIF19 | 161 | -1.41 | 5.50E-07 |
| SST | 36849 | -2.10 | 2.34E-11 | MAPK8IP2 | 527 | -1.17 | 0.000183946 | STC2 | 160 | 1.02 | 0.000379493 |
| SPARC | 35778 | -1.13 | 0.021750423 | ADGRL4 | 523 | -1.06 | 0.045937794 | PPP4R4 | 159 | 1.25 | 3.39E-06 |
| COL3A1 | 28990 | -3.37 | 4.16E-08 | CA4 | 523 | -1.92 | 0.000294972 | ELN | 159 | -3.48 | 0.003445876 |
| CFTR | 28453 | 1.11 | 0.00058138 | SLC25A53 | 518 | -3.20 | 4.86E-31 | MYBL1 | 159 | -1.48 | 1.92E-08 |
| CFD | 21617 | 1.70 | 3.54E-05 | TMEM151B | 517 | 1.17 | 1.25E-18 | KCNK3 | 159 | -1.43 | 0.009331262 |
| COL1A1 | 21532 | -3.19 | 5.09E-06 | CCNB1 | 515 | -3.41 | 3.09E-24 | SEMA4A | 159 | -1.15 | 3.22E-06 |
| FN1 | 21369 | -2.34 | 0.000209679 | GAD2 | 507 | -4.25 | 1.24E-32 | CCNB3 | 158 | -3.62 | 4.40E-17 |
| CHGB | 20938 | -3.13 | 1.01E-30 | SLC25A33 | 504 | 1.21 | 1.52E-07 | ALDH1L1 | 158 | 1.00 | 0.028380052 |
| TXNIP | 19178 | -1.40 | 8.10E-14 | CDC42EP2 | 500 | 1.45 | 2.83E-12 | MALRD1 | 157 | -2.55 | 1.74E-07 |
| COL6A3 | 17813 | -3.29 | 8.86E-08 | CD248 | 500 | -2.12 | 3.24E-05 | DUSP2 | 157 | 1.46 | 4.44E-06 |
| COL1A2 | 16193 | -2.81 | 5.93E-06 | UPF3A | 497 | -1.10 | 1.47E-05 | ARHGAP24 | 157 | -1.04 | 4.07E-05 |
| PCSK1 | 14037 | -3.86 | 5.31E-43 | PAK3 | 496 | -1.20 | 0.000107782 | CPED1 | 156 | -1.96 | 0.000128548 |
| DIRAS3 | 13930 | -2.92 | 7.89E-28 | CENPE | 496 | -2.71 | 4.56E-20 | CCDC68 | 156 | -1.43 | 7.46E-07 |
| TM4SF4 | 13446 | -1.21 | 8.36E-08 | DUOX2 | 490 | 2.02 | 0.008520779 | CACNA1E | 156 | -1.76 | 2.52E-06 |
| CPE | 13424 | -2.32 | 7.97E-13 | GPR160 | 489 | -1.19 | 0.000593858 | GPX8 | 155 | -1.43 | 0.001035014 |
| GATM | 11443 | -2.02 | 0.012985456 | RAB9B | 489 | -2.58 | 4.12E-22 | CDKN3 | 155 | -1.40 | 5.38E-08 |
| PCSK2 | 11191 | -3.02 | 9.43E-17 | EPPK1 | 486 | 1.12 | 0.047735623 | KIAA1755 | 154 | -2.91 | 0.000793464 |
| DMBT1 | 10629 | -1.24 | 0.041060281 | ST8SIA1 | 485 | -1.34 | 9.55E-10 | CEP128 | 154 | -1.19 | 2.60E-07 |
| PPY | 10557 | -2.06 | 0.016447863 | MAP6D1 | 482 | -1.70 | 0.000910279 | AGTR1 | 153 | -1.71 | 0.006708463 |
| TFRC | 10342 | 1.28 | 1.35E-09 | PLCG2 | 479 | -1.07 | 5.67E-05 | NRXN1 | 152 | -1.62 | 1.21E-05 |
| ACLY | 10239 | -1.29 | 3.69E-12 | TSPAN18 | 477 | -1.65 | 0.00089487 | TMEM145 | 151 | -1.07 | 0.000681621 |
| SCG2 | 9725 | -2.69 | 1.25E-18 | SULF1 | 476 | -1.67 | 0.001079832 | MIOX | 151 | 1.14 | 0.000585655 |
| PEG10 | 9427 | -2.50 | 1.96E-16 | CLSPN | 472 | -1.76 | 9.97E-20 | UCP2 | 151 | -1.02 | 0.04991111 |
| FGFR2 | 9406 | 1.39 | 3.39E-07 | TNFAIP3 | 467 | 1.14 | 0.000910206 | GUCA2B | 151 | -1.29 | 0.047626243 |
| PAPPA2 | 8577 | -3.44 | 5.43E-12 | CPM | 467 | -1.61 | 6.83E-06 | LRRN2 | 150 | -1.58 | 5.19E-05 |
| SCGN | 8275 | -2.73 | 8.53E-15 | LRRC32 | 465 | -1.79 | 0.003878596 | DAB2 | 150 | -1.16 | 0.00063944 |
| MMP2 | 8243 | -1.58 | 1.91E-06 | KIF22 | 464 | -2.05 | 2.57E-24 | SNTG1 | 149 | -2.03 | 1.57E-06 |
| SCG5 | 8234 | -1.46 | 5.95E-08 | NCALD | 463 | -1.94 | 6.35E-09 | SLPI | 148 | -1.25 | 0.004494813 |
| HIF1A | 7796 | 1.06 | 1.84E-18 | CHAF1A | 461 | -1.55 | 2.63E-25 | KLB | 147 | -1.78 | 1.75E-06 |
| HMGN2 | 7484 | -1.59 | 7.57E-20 | CTSZ | 461 | -1.61 | 2.95E-06 | SSTR3 | 147 | -1.39 | 0.000632727 |
| NUPR1 | 7448 | 1.54 | 5.55E-05 | PLCD3 | 460 | 1.01 | 7.98E-06 | ADGRV1 | 147 | -1.78 | 9.55E-10 |
| COL5A2 | 7390 | -1.19 | 0.002546245 | NEDD4 | 460 | 1.35 | 4.57E-16 | DDIAS | 145 | -1.01 | 0.000119225 |
| BIRC3 | 7312 | 1.36 | 8.07E-07 | KIFC1 | 459 | -2.48 | 1.20E-16 | TNFAIP6 | 145 | -1.58 | 0.001587416 |
| BIRC3 | 7312 | 1.36 | 8.07E-07 | GPX3 | 458 | -3.07 | 9.18E-26 | SPEF2 | 145 | -1.14 | 1.27E-05 |
| STOM | 6925 | 1.30 | 4.35E-15 | ASB9 | 457 | -3.20 | 5.09E-14 | FOXS1 | 145 | -1.56 | 0.00031232 |
| CTGF | 6912 | 1.16 | 2.67E-11 | AFP | 457 | -3.10 | 9.59E-08 | RMI2 | 145 | -2.94 | 3.52E-19 |
| ARHGAP21 | 6759 | 1.13 | 0.000128494 | FBLN7 | 455 | -1.86 | 2.65E-11 | CTHRC1 | 142 | -1.87 | 0.000398648 |
| COL5A1 | 6164 | -1.94 | 0.000125757 | ARHGEF28 | 452 | 1.10 | 1.25E-10 | C6 | 141 | -1.76 | 0.002603374 |
| RALGAPA2 | 6032 | 1.07 | 1.27E-12 | RBP4 | 450 | 1.55 | 9.44E-06 | DEPDC1 | 141 | -3.10 | 2.05E-15 |
| VGF | 5959 | -2.29 | 1.25E-18 | PPM1E | 450 | -2.36 | 1.67E-12 | CCNE2 | 139 | -1.48 | 9.45E-07 |
| PGA5 | 5909 | 1.61 | 4.62E-07 | FSD1L | 449 | -1.17 | 4.91E-13 | TDRKH | 139 | -1.07 | 0.00082854 |
| PRSS2 | 5768 | -5.88 | 5.93E-08 | CD74 | 448 | -1.00 | 0.024384886 | CDCA3 | 139 | -3.41 | 1.12E-19 |

| | | | | | | | | | | | |
|---|---|---|---|---|---|---|---|---|---|---|---|
| SLC7A8 | 5724 | -1.89 | 1.05E-09 | PKNOX2 | 446 | -1.43 | 4.01E-05 | UBE2T | 137 | -1.72 | 9.23E-08 |
| PDGFRB | 5569 | -1.57 | 0.001915103 | ALS2CL | 446 | 1.26 | 0.000641243 | KIF18A | 137 | -2.01 | 1.73E-10 |
| CGNL1 | 5451 | 1.01 | 6.01E-05 | TSPAN12 | 444 | -1.09 | 0.000181598 | SCML2 | 137 | -1.09 | 3.81E-06 |
| IGFBP4 | 5415 | -2.52 | 1.86E-06 | CHCHD10 | 444 | 1.11 | 1.90E-07 | HCN4 | 137 | -1.05 | 0.01971828 |
| CTSC | 5377 | 1.26 | 1.54E-05 | PRICKLE2 | 443 | -1.58 | 0.000124448 | FAM184A | 137 | -1.53 | 8.68E-05 |
| PMEPA1 | 5323 | -1.20 | 7.81E-06 | PRELID2 | 443 | 1.05 | 1.71E-06 | CAPS2 | 137 | -1.35 | 4.92E-09 |
| SLC7A2 | 5273 | -2.14 | 4.57E-13 | KIF23 | 442 | -1.89 | 4.22E-13 | CENPO | 136 | -1.74 | 6.90E-14 |
| HEPACAM2 | 5190 | -2.51 | 1.43E-19 | ACTA1 | 442 | 1.02 | 0.03718862 | SLC8A2 | 136 | -1.49 | 5.67E-05 |
| BGN | 5080 | -2.85 | 0.000108854 | C1S | 442 | -1.29 | 0.004394978 | UABP-2 | 136 | 1.31 | 0.037674938 |
| HMGA1 | 5046 | 1.58 | 4.41E-16 | KIF20A | 438 | -3.10 | 8.12E-14 | CBLN4 | 136 | -1.86 | 0.014135776 |
| STMN1 | 4959 | -1.08 | 1.83E-14 | EMB | 433 | -1.98 | 1.12E-09 | ST8SIA3 | 136 | -1.73 | 1.74E-08 |
| TPD52 | 4880 | -1.31 | 1.55E-10 | ADAMTS12 | 428 | -1.59 | 0.048490154 | CCDC198 | 134 | -1.17 | 4.77E-08 |
| IER3 | 4857 | 1.37 | 0.000533401 | KIAA1147 | 425 | 1.19 | 8.35E-10 | MASTL | 134 | -3.13 | 3.07E-18 |
| COL5A3 | 4843 | -1.28 | 0.039243306 | CCL2 | 424 | 1.11 | 0.000458862 | BRIP1 | 134 | -1.50 | 2.24E-08 |
| CYR61 | 4739 | 2.36 | 4.94E-11 | CMKLR1 | 422 | -3.08 | 6.01E-05 | DUOX1 | 134 | 2.60 | 3.83E-18 |
| ANKRD1 | 4729 | 1.88 | 3.08E-06 | HMMR | 421 | -3.08 | 6.86E-34 | XRCC2 | 134 | -1.65 | 9.44E-11 |
| CREB3L1 | 4704 | 1.19 | 8.22E-08 | PPP1R1A | 420 | -1.42 | 0.002828301 | HCN3 | 133 | -1.07 | 1.01E-05 |
| CXCL8 | 4516 | 2.67 | 0.000896086 | PIM1 | 418 | 2.39 | 9.48E-15 | CDC6 | 133 | -1.72 | 8.40E-11 |
| FSTL1 | 4364 | -2.15 | 9.85E-06 | RADX | 413 | -1.79 | 1.23E-22 | FBXO5 | 133 | -1.83 | 9.44E-11 |
| COL12A1 | 4350 | -1.03 | 0.001042588 | LRRC75A | 412 | 1.33 | 1.36E-15 | MELK | 133 | -2.24 | 1.06E-14 |
| GRIA3 | 4295 | -1.99 | 3.13E-09 | SCN3A | 411 | -1.69 | 9.78E-13 | KIF18B | 132 | -2.78 | 2.41E-10 |
| MEST | 4075 | -1.53 | 4.41E-05 | IQGAP3 | 408 | -2.62 | 8.17E-25 | EDIL3 | 132 | -2.57 | 0.000101996 |
| TTR | 4028 | -2.60 | 1.11E-25 | CACNA1C | 406 | -1.19 | 0.001577069 | LTC4S | 132 | -2.51 | 0.010836902 |
| CTRC | 3990 | -3.50 | 0.00201389 | MORC4 | 405 | 1.13 | 1.12E-12 | FAM83D | 132 | -1.85 | 5.75E-09 |
| CPLX2 | 3963 | -1.83 | 7.29E-10 | BRCA1 | 405 | -1.45 | 2.94E-15 | ASTN2 | 131 | -2.32 | 4.07E-11 |
| TF | 3886 | -3.05 | 2.09E-10 | FAH | 405 | -1.13 | 5.14E-08 | DTL | 131 | -3.41 | 1.80E-19 |
| TP53BP2 | 3869 | 1.01 | 0.000218176 | CLMP | 404 | -2.20 | 3.56E-10 | BEX5 | 130 | -2.42 | 3.39E-12 |
| PTPRN | 3830 | -2.03 | 3.00E-19 | KNTC1 | 403 | -2.73 | 1.97E-18 | IGFBPL1 | 130 | -2.64 | 1.49E-08 |
| CNTN1 | 3702 | -2.02 | 3.81E-11 | GNG4 | 403 | -1.11 | 1.31E-06 | BMPR1B | 130 | -1.75 | 0.00141468 |
| KIFC3 | 3692 | 1.50 | 2.80E-05 | BRINP2 | 401 | -1.64 | 0.000506262 | KIF26B | 128 | -1.06 | 0.015364084 |
| SCG3 | 3684 | -1.57 | 5.88E-10 | C4BPA | 399 | 1.13 | 0.039269246 | CAMK1D | 128 | -1.07 | 0.026956826 |
| RAP1GAP2 | 3679 | -1.09 | 0.011806052 | C4BPA | 399 | 1.13 | 0.039269246 | SPDL1 | 128 | -1.48 | 3.07E-07 |
| LRRN3 | 3662 | -3.06 | 1.47E-15 | TK1 | 399 | -2.14 | 1.44E-20 | PCK1 | 128 | 2.69 | 0.000385529 |
| CBX6 | 3646 | -1.12 | 1.88E-06 | DLK1 | 398 | -3.78 | 0.003701328 | TMEM200A | 128 | -1.78 | 0.000453324 |
| SDCBP2 | 3625 | 1.92 | 7.78E-07 | FANCD2 | 398 | -1.59 | 4.96E-14 | GAS1 | 127 | -1.50 | 2.07E-05 |
| RGS4 | 3614 | -1.49 | 3.67E-07 | KIAA1324L | 394 | -1.02 | 2.61E-07 | GRIK4 | 126 | -1.77 | 0.00088173 |
| PRXL2A | 3584 | 1.04 | 7.85E-15 | PHF19 | 393 | -1.48 | 1.58E-12 | SLC16A9 | 126 | -5.29 | 8.65E-16 |
| ABCC8 | 3418 | -2.82 | 1.23E-12 | SLC4A10 | 393 | -1.20 | 0.016042266 | MTFR2 | 126 | -2.53 | 1.83E-13 |
| PTPRN2 | 3355 | -2.16 | 1.16E-12 | CERKL | 392 | -1.96 | 1.03E-07 | CDCA2 | 125 | -2.04 | 1.37E-11 |
| ICAM1 | 3298 | 1.70 | 1.93E-07 | ADCY3 | 392 | -1.56 | 3.75E-07 | CITED4 | 125 | 1.89 | 0.020971848 |
| SLC4A7 | 3264 | 1.26 | 1.47E-06 | GOLGA7B | 391 | -1.28 | 6.31E-07 | NRG1 | 125 | 1.26 | 0.004399301 |
| CRIM1 | 3245 | 1.06 | 3.04E-07 | NELL2 | 391 | -2.35 | 2.30E-22 | ABHD15 | 125 | -1.53 | 0.000378941 |
| SUN2 | 3223 | 1.10 | 5.53E-26 | ADRA2B | 389 | -2.96 | 0.000544705 | RASL11A | 125 | -1.07 | 8.44E-06 |
| SOD2 | 3204 | 1.53 | 1.05E-14 | ACADL | 388 | -1.03 | 2.33E-06 | LEPR | 124 | -1.81 | 0.002024929 |
| COL16A1 | 3108 | -1.60 | 3.55E-07 | INA | 387 | -1.06 | 7.78E-12 | ADORA2B | 124 | 1.35 | 0.000245354 |
| GPRC5B | 3099 | 1.27 | 9.88E-05 | PBK | 387 | -3.16 | 1.45E-26 | SPC24 | 124 | -1.90 | 4.70E-08 |
| PDK4 | 3096 | -1.07 | 3.23E-10 | SLIT2 | 386 | -1.64 | 5.84E-11 | CCDC60 | 123 | -2.40 | 1.26E-07 |
| TUSC3 | 3066 | -1.14 | 1.23E-06 | PROM2 | 384 | 1.33 | 0.000669518 | CCDC60 | 123 | -2.40 | 1.26E-07 |
| TMPRSS2 | 3058 | 1.02 | 0.023655629 | SLC4A8 | 384 | -1.29 | 3.32E-06 | MAGEL2 | 122 | -1.71 | 3.23E-05 |

| | | | | | | | | | | | |
|---|---|---|---|---|---|---|---|---|---|---|---|
| NPTX2 | 3042 | -1.47 | 8.74E-13 | NFASC | 382 | -1.57 | 3.19E-06 | ASPN | 122 | -3.99 | 3.52E-05 |
| SPIDR | 3029 | 1.42 | 3.56E-07 | STRIP2 | 381 | -1.14 | 1.02E-10 | SGO1 | 121 | -3.54 | 2.03E-16 |
| WWC1 | 2955 | 1.13 | 4.93E-09 | MCRIP2 | 381 | 1.38 | 3.17E-13 | JAKMIP2 | 121 | -1.58 | 1.03E-05 |
| PAX6 | 2938 | -2.07 | 1.37E-10 | KIF2C | 381 | -2.20 | 1.74E-11 | CCR1 | 120 | -1.24 | 0.014471831 |
| ESM1 | 2916 | -1.41 | 0.030695777 | NCAPG | 380 | -2.00 | 8.16E-10 | MYO1F | 120 | -1.33 | 0.001987001 |
| RETREG1 | 2836 | 1.30 | 1.73E-21 | LRRK2 | 380 | -1.21 | 9.02E-07 | SLC24A2 | 120 | -3.33 | 3.95E-07 |
| PXDN | 2827 | -1.02 | 0.017188826 | RAPGEF4 | 377 | 1.08 | 0.000768686 | KIF24 | 120 | -2.03 | 5.62E-12 |
| ANTXR1 | 2810 | -1.69 | 2.90E-06 | CAMK2B | 376 | -1.42 | 2.34E-11 | IL1A | 119 | 3.09 | 0.000884519 |
| EREG | 2804 | 1.01 | 0.000271879 | SLC16A12 | 376 | -2.08 | 0.001312308 | BMX | 118 | -1.21 | 0.008095169 |
| FBN1 | 2770 | -2.43 | 3.68E-06 | ITGA4 | 376 | -2.19 | 4.72E-06 | CENPU | 118 | -1.65 | 2.17E-11 |
| SFRP1 | 2741 | -2.82 | 2.36E-05 | MXD3 | 375 | -1.28 | 1.07E-09 | NAP1L5 | 118 | -1.21 | 0.000581142 |
| SLC43A2 | 2685 | -1.96 | 3.37E-09 | LHFPL4 | 374 | -1.85 | 3.88E-15 | SLC18A1 | 118 | -2.10 | 0.000108134 |
| ARRDC4 | 2665 | -1.09 | 9.29E-09 | CYGB | 373 | -2.89 | 1.75E-05 | ACTG2 | 118 | -2.77 | 0.049943976 |
| LTA4H | 2627 | 1.10 | 3.34E-09 | PLCL2 | 373 | -2.17 | 3.40E-09 | CDCA7 | 117 | -1.13 | 0.000158513 |
| F3 | 2618 | 1.00 | 0.000923559 | TNR | 371 | -1.46 | 3.20E-05 | LMCD1 | 117 | -1.43 | 0.001967506 |
| KIAA1324 | 2567 | -2.66 | 8.53E-10 | CABP7 | 368 | -1.24 | 0.001553627 | IRF5 | 117 | 1.00 | 0.020027228 |
| EPS8L2 | 2561 | 1.45 | 1.62E-11 | ADM | 366 | 1.19 | 0.022758064 | PRODH2 | 116 | -1.35 | 2.58E-06 |
| KLF10 | 2556 | -1.44 | 1.53E-14 | SLC39A5 | 365 | -1.48 | 0.013561549 | CENPS | 116 | -1.44 | 6.04E-07 |
| ATP8 | 2488 | -3.71 | 0.041721548 | SLC39A8 | 365 | 1.35 | 0.003675912 | C1QB | 116 | -1.74 | 2.38E-06 |
| CEBPD | 2470 | 1.57 | 3.71E-05 | RASSF5 | 363 | -1.00 | 0.000587039 | FRMPD4 | 116 | -1.35 | 0.041297891 |
| SLC22A17 | 2468 | -1.28 | 0.000131654 | SLC35F3 | 361 | -1.41 | 0.000644756 | FRRS1L | 115 | -1.02 | 0.03577703 |
| MAPT | 2446 | -1.35 | 6.03E-28 | EML6 | 360 | -1.94 | 2.66E-08 | TRPC5 | 115 | -1.54 | 0.001067882 |
| BMP1 | 2445 | -1.42 | 2.63E-07 | CDKN1C | 358 | 1.41 | 3.24E-06 | MTHFD2L | 114 | 1.15 | 1.81E-05 |
| SYT13 | 2432 | -2.27 | 1.85E-10 | BVES | 357 | -1.98 | 0.002320713 | TRPM6 | 114 | 1.57 | 5.45E-06 |
| SLC38A4 | 2415 | -1.63 | 3.79E-06 | TIMELESS | 357 | -1.58 | 1.75E-05 | ASTN1 | 114 | -1.16 | 0.002211106 |
| APLP1 | 2385 | -1.11 | 7.95E-06 | KIF4A | 356 | -2.81 | 5.15E-17 | BST2 | 113 | 1.01 | 0.011715998 |
| ANPEP | 2340 | -1.82 | 0.000491422 | PDE1C | 354 | -1.02 | 1.22E-05 | FAM221A | 113 | 1.29 | 0.000118372 |
| ALDH1A1 | 2299 | 2.19 | 0.000831526 | SLC5A1 | 351 | 1.08 | 0.001171821 | SLC5A9 | 113 | 1.07 | 0.004090949 |
| SLC5A6 | 2282 | 1.18 | 9.24E-10 | UCN3 | 351 | -3.36 | 1.14E-15 | NMB | 113 | 1.12 | 0.010755605 |
| GNAO1 | 2265 | -1.78 | 3.58E-10 | ARHGDIB | 351 | -1.82 | 0.000431957 | HAPLN3 | 113 | 1.09 | 5.30E-06 |
| POSTN | 2253 | -2.98 | 0.000501645 | PEX5L | 350 | -1.63 | 0.000275594 | MR1 | 112 | 1.00 | 0.034911782 |
| ENPP2 | 2237 | -1.43 | 0.018091478 | BUB1 | 349 | -3.16 | 4.79E-23 | ZNF467 | 111 | 1.91 | 1.24E-10 |
| IGFBP7 | 2165 | -2.43 | 1.15E-05 | CDCA5 | 349 | -2.78 | 9.95E-29 | SERPINA11 | 111 | 2.11 | 1.09E-06 |
| MGST1 | 2134 | 1.17 | 1.55E-05 | HK2 | 349 | 1.63 | 0.001549819 | CAPN6 | 110 | -4.24 | 0.000408586 |
| GAS6 | 2121 | 1.24 | 4.72E-22 | TBX3 | 349 | -1.18 | 4.09E-05 | CYP7B1 | 109 | -1.19 | 0.003179528 |
| ADAMTS4 | 2111 | -1.07 | 0.011569647 | IL33 | 348 | -1.51 | 0.017172364 | RBP5 | 109 | -2.24 | 0.00808011 |
| SNTB1 | 2096 | -1.10 | 4.78E-05 | GLB1L | 345 | -1.06 | 3.24E-10 | DHRS3 | 109 | 2.21 | 2.44E-08 |
| NFKBIA | 2054 | 1.33 | 8.82E-05 | RASL11B | 343 | -1.00 | 0.000752615 | NT5E | 108 | -1.86 | 0.00630736 |
| RGS5 | 2021 | -2.28 | 0.000292509 | CHTF18 | 343 | -1.35 | 2.74E-18 | SRPX2 | 108 | -1.62 | 0.001185819 |
| LIPA | 1979 | -1.02 | 2.45E-08 | SGO2 | 341 | -1.59 | 9.54E-10 | ST8SIA2 | 108 | -2.06 | 1.15E-07 |
| UGCG | 1969 | 1.01 | 1.88E-08 | S1PR3 | 341 | -1.80 | 0.00091143 | P2RY2 | 108 | 1.86 | 1.16E-09 |
| PFKFB3 | 1965 | 1.24 | 8.90E-07 | PYY | 340 | -2.76 | 0.012070253 | HAPLN1 | 107 | -2.00 | 0.011617796 |
| TRH | 1948 | -3.71 | 1.28E-12 | LAMC3 | 339 | -3.98 | 0.000224571 | E2F8 | 107 | -3.42 | 1.83E-19 |
| TMEM30B | 1920 | 1.47 | 6.99E-09 | DPP10 | 338 | -2.70 | 5.07E-12 | COL4A4 | 107 | 2.30 | 0.000271341 |
| COL14A1 | 1880 | -1.41 | 3.43E-06 | ASPM | 338 | -2.86 | 4.09E-16 | MSR1 | 106 | -2.53 | 1.88E-09 |
| COL14A1 | 1880 | -1.41 | 3.43E-06 | SYT17 | 336 | -1.40 | 1.89E-05 | GLDC | 106 | -1.15 | 0.007988053 |
| CELF3 | 1874 | -1.15 | 1.48E-05 | EFEMP1 | 336 | -2.37 | 0.000354273 | SLC19A3 | 106 | 1.27 | 0.000774913 |
| ARRDC3 | 1858 | -1.16 | 2.54E-12 | SEZ6 | 335 | -2.43 | 6.70E-30 | YBX2 | 106 | 1.70 | 6.36E-05 |
| APOE | 1858 | -2.09 | 1.42E-06 | CITED1 | 335 | -4.12 | 2.09E-16 | NLRC5 | 106 | -1.17 | 0.020265999 |

| | | | | | | | | | | | |
|---|---|---|---|---|---|---|---|---|---|---|---|
| ABAT | 1850 | 1.54 | 0.044683861 | ZWILCH | 331 | -1.07 | 2.94E-07 | VSTM4 | 105 | -1.19 | 0.000593027 |
| UNC5CL | 1835 | 1.21 | 8.79E-05 | DLGAP5 | 329 | -2.71 | 3.16E-17 | SCN4B | 105 | -3.52 | 4.60E-10 |
| MCM6 | 1830 | -1.50 | 1.44E-21 | CNTLN | 326 | -1.74 | 2.67E-11 | ERCC6L | 105 | -3.20 | 2.16E-19 |
| RAB26 | 1827 | -1.51 | 1.07E-13 | SLC16A14 | 326 | -2.38 | 2.53E-19 | CENPW | 104 | -1.74 | 9.20E-06 |
| PDX1 | 1823 | -1.14 | 4.16E-13 | RUNX1T1 | 325 | -1.93 | 4.51E-08 | SYNPO2 | 104 | -1.69 | 0.003234721 |
| IGFBP2 | 1802 | -1.50 | 0.000377459 | SLC8A1 | 325 | -1.42 | 2.65E-09 | DPEP2 | 104 | -3.31 | 1.24E-12 |
| TOP2A | 1796 | -3.26 | 1.62E-19 | RAB29 | 325 | 1.21 | 6.41E-08 | CPNE4 | 104 | -1.57 | 1.92E-07 |
| GJA1 | 1793 | -1.10 | 0.000269759 | FANCI | 324 | -1.09 | 1.02E-10 | PLB1 | 104 | -2.35 | 3.76E-07 |
| LUM | 1771 | -2.72 | 6.24E-10 | BUB1B | 323 | -3.26 | 2.99E-19 | LOXL1 | 103 | -2.18 | 0.003071386 |
| THBS2 | 1769 | -2.54 | 0.008832528 | FANCA | 322 | -1.27 | 3.18E-12 | E2F2 | 103 | -3.04 | 2.63E-13 |
| SYT4 | 1759 | -1.49 | 2.61E-05 | PLXNA4 | 322 | -1.58 | 0.008104332 | LRCH2 | 103 | -1.28 | 0.003548154 |
| PARD6B | 1749 | 1.45 | 7.63E-10 | ISG12(A) | 321 | 1.58 | 2.22E-06 | GFPT2 | 103 | -2.04 | 5.80E-06 |
| GNG2 | 1747 | -1.33 | 3.78E-05 | PDGFRA | 321 | -1.84 | 3.63E-05 | AGT | 103 | -1.23 | 0.007842711 |
| ECM1 | 1742 | -1.19 | 0.002828936 | KLF15 | 321 | 2.00 | 3.90E-18 | PTGFR | 103 | -1.04 | 0.033667591 |
| SESTD1 | 1732 | 1.31 | 3.75E-11 | NUF2 | 320 | -2.41 | 8.07E-20 | BCAS1 | 103 | -1.19 | 0.003862313 |
| TINAGL1 | 1710 | 1.90 | 9.46E-08 | MAML3 | 320 | -1.21 | 0.000187157 | CCDC65 | 102 | -1.24 | 0.001522949 |
| PPARGC1A | 1702 | 1.36 | 5.54E-08 | CERCAM | 319 | -1.19 | 1.61E-05 | GPR119 | 102 | -3.97 | 1.30E-11 |
| KCNMA1 | 1701 | -1.52 | 0.00010979 | ANXA13 | 317 | 1.41 | 0.026032333 | LGALS2 | 102 | 1.49 | 0.01579432 |
| ENG | 1694 | -1.83 | 2.34E-06 | ESPL1 | 317 | -2.91 | 3.86E-15 | SPRED3 | 102 | -1.24 | 0.000298286 |
| LAMB3 | 1688 | 1.04 | 0.008204303 | FAM111A | 315 | -1.43 | 0.007616836 | ATP6V1B1 | 101 | -2.08 | 5.78E-07 |
| DDC | 1668 | -1.55 | 2.04E-08 | SHISA2 | 314 | -1.89 | 1.94E-09 | NCF2 | 100 | -1.05 | 0.000831526 |
| DNAJC12 | 1646 | -1.79 | 5.33E-13 | CIT | 313 | -2.27 | 8.08E-20 | KIAA0319 | 100 | -1.40 | 9.23E-07 |
| LMNB1 | 1615 | -1.09 | 2.42E-24 | LIPH | 310 | 1.11 | 0.013574894 | ZGRF1 | 100 | -2.71 | 5.73E-11 |
| RND1 | 1605 | 1.31 | 0.000165222 | UBE2C | 310 | -3.52 | 2.35E-15 | EYA2 | 99 | 1.28 | 4.35E-06 |
| OLFM4 | 1591 | 2.86 | 0.008113899 | ANKS4B | 308 | -1.14 | 7.07E-06 | PDYN | 99 | 1.75 | 0.000920992 |
| NRP1 | 1562 | -1.59 | 2.31E-06 | ZNF367 | 307 | -3.06 | 1.14E-20 | ARL4D | 98 | -2.15 | 2.65E-06 |
| SFRP5 | 1550 | -1.42 | 0.035354704 | SPTB | 306 | -1.25 | 2.41E-05 | PACRG | 98 | -1.72 | 1.96E-06 |
| RAB31 | 1543 | -1.59 | 6.82E-07 | P2RY1 | 304 | -1.94 | 1.20E-05 | INKA1 | 97 | -1.23 | 0.021180288 |
| CDH11 | 1538 | -3.60 | 8.07E-06 | KIF15 | 302 | -2.94 | 8.85E-21 | GALP | 97 | -2.47 | 2.33E-06 |
| MLXIPL | 1538 | -1.31 | 0.000639862 | PLXDC2 | 300 | -1.31 | 3.05E-08 | KLHL41 | 96 | -1.79 | 0.000284983 |
| SEZ6L2 | 1533 | -1.96 | 8.27E-12 | EGFLAM | 298 | -3.11 | 1.25E-06 | SLC2A12 | 95 | 1.39 | 0.026250446 |
| CXCL14 | 1527 | -3.07 | 3.53E-06 | SPOCK1 | 297 | -1.52 | 0.044886681 | EXO1 | 95 | -2.52 | 9.34E-13 |
| PLVAP | 1502 | -1.32 | 0.000852303 | PGBD5 | 296 | -1.34 | 1.02E-08 | LRRC49 | 94 | -1.76 | 1.79E-07 |
| NEUROD1 | 1502 | -2.43 | 8.81E-18 | COLEC12 | 296 | -2.71 | 4.01E-08 | GEN1 | 94 | -2.12 | 1.39E-08 |
| CDK18 | 1482 | 1.86 | 1.98E-17 | DOCK8 | 293 | -1.17 | 2.97E-05 | NYAP2 | 94 | -1.37 | 0.010829883 |
| TGFBI | 1466 | -2.23 | 1.39E-12 | IL17RB | 292 | 1.43 | 1.86E-07 | SCARA5 | 94 | -1.16 | 0.007210632 |
| FZD7 | 1456 | 1.00 | 2.32E-05 | COL8A1 | 291 | -3.41 | 0.000661264 | ZWINT | 93 | -1.85 | 6.94E-07 |
| CXCL2 | 1454 | 2.72 | 0.000378006 | S100A4 | 291 | 1.41 | 0.000986054 | DISP2 | 93 | -1.96 | 4.55E-09 |
| CXCL2 | 1454 | 2.72 | 0.000378006 | MCM10 | 290 | -2.02 | 1.02E-13 | CPXM1 | 93 | -2.18 | 0.008453551 |
| MRC2 | 1453 | -2.98 | 2.86E-05 | PLSCR4 | 286 | -1.29 | 0.000124456 | A1CF | 93 | -1.15 | 0.000161919 |
| COQ9 | 1450 | 1.04 | 9.72E-17 | CHST11 | 286 | -1.47 | 0.000130126 | FAP | 93 | -3.06 | 0.000174348 |
| PHYH | 1444 | 1.10 | 7.70E-11 | PCSK5 | 285 | -1.50 | 2.95E-06 | C3orf14 | 93 | -1.16 | 0.000473221 |
| SMC2 | 1444 | -1.52 | 3.14E-13 | ITGA1 | 284 | -1.04 | 0.001409272 | NPL | 93 | -1.47 | 3.97E-05 |
| KNG1 | 1431 | 3.03 | 2.42E-07 | NALCN | 283 | -1.91 | 4.11E-07 | CALML4 | 93 | -1.27 | 0.000166839 |
| NOV | 1431 | 1.82 | 7.00E-05 | FEN1 | 283 | -1.18 | 4.52E-08 | CEP55 | 92 | -1.98 | 9.82E-08 |
| TANC1 | 1431 | 1.19 | 1.01E-14 | ISLR | 282 | -4.84 | 0.000343361 | CDC23 | 92 | -1.04 | 0.000509225 |
| SYP | 1430 | -1.25 | 5.28E-08 | FFAR3 | 282 | -2.18 | 1.73E-09 | ARNTL2 | 91 | -1.12 | 0.000145379 |
| ADAM12 | 1418 | -3.02 | 2.92E-06 | FNDC1 | 282 | -1.63 | 0.001549857 | LRRN1 | 91 | -1.48 | 0.000330998 |
| BAIAP3 | 1417 | -1.13 | 7.36E-05 | PEAR1 | 281 | -1.14 | 0.016226938 | NPTX1 | 90 | -1.45 | 0.001371209 |

| | | | | | | | | | | | |
|---|---|---|---|---|---|---|---|---|---|---|---|
| PLAU | 1399 | 1.28 | 3.14E-05 | TTK | 279 | -3.31 | 6.22E-31 | C4BPB | 89 | -2.78 | 6.58E-12 |
| MCM4 | 1394 | -1.45 | 2.71E-20 | DDIT3 | 278 | 1.10 | 1.17E-06 | KCNF1 | 89 | 1.90 | 0.000178609 |
| COL15A1 | 1367 | -1.76 | 0.000133802 | CSPG4 | 278 | -2.18 | 0.000435493 | TSHZ3 | 89 | -1.48 | 0.000287904 |
| CNTN3 | 1349 | -1.36 | 0.000150991 | ROR2 | 277 | -1.64 | 0.000104022 | GABBR2 | 88 | -1.56 | 3.48E-05 |
| LRP4 | 1342 | 1.76 | 2.09E-18 | E2F1 | 277 | -2.80 | 4.31E-22 | SAMD15 | 87 | -1.46 | 9.55E-06 |
| TMEM184A | 1341 | 1.02 | 3.95E-09 | ARHGAP33 | 277 | -1.03 | 1.43E-07 | HHIP | 87 | -1.58 | 0.01709187 |
| ALDH1A3 | 1335 | -3.49 | 9.04E-05 | CDT1 | 276 | -1.92 | 5.68E-21 | SVEP1 | 87 | -2.05 | 0.002449729 |
| ANKH | 1328 | -1.21 | 5.21E-07 | PDE4A | 276 | 1.14 | 6.14E-10 | C20orf96 | 86 | -1.67 | 3.16E-05 |
| EMILIN1 | 1318 | -1.59 | 6.03E-05 | ACP5 | 275 | -1.97 | 0.000723012 | DES | 86 | -3.39 | 0.027197789 |
| BCL10 | 1309 | 1.09 | 2.31E-07 | CENPH | 274 | -1.14 | 3.00E-09 | KCP | 85 | 1.78 | 0.000324202 |
| PLCXD3 | 1304 | -2.03 | 2.44E-16 | ADGRB2 | 272 | -1.13 | 3.28E-07 | COL8A2 | 84 | -3.62 | 0.000843115 |
| HMGB2 | 1297 | -1.67 | 4.94E-20 | WNT7B | 272 | 1.28 | 2.24E-10 | SLC35D3 | 84 | -1.14 | 0.010941957 |
| CCL20 | 1293 | 3.70 | 3.44E-05 | NEK2 | 271 | -3.57 | 1.16E-20 | FAM167A | 84 | -1.47 | 0.000352977 |
| OXCT1 | 1290 | 1.03 | 8.62E-18 | CDK5R2 | 269 | -1.46 | 3.64E-05 | PRRX2 | 84 | -2.26 | 0.001844756 |
| MYO3A | 1284 | -3.58 | 1.31E-16 | TMEM130 | 267 | -1.93 | 1.01E-10 | PIMREG | 84 | -3.06 | 3.44E-11 |
| LRP11 | 1284 | -1.64 | 1.53E-14 | MAP3K8 | 264 | 1.21 | 0.001083266 | C7 | 83 | -5.13 | 0.000230572 |
| LCN2 | 1283 | 1.28 | 0.004893369 | GRM4 | 264 | -2.82 | 1.87E-17 | HJURP | 82 | -4.15 | 1.56E-12 |
| HTRA3 | 1273 | -2.89 | 4.00E-14 | CYP3A39 | 264 | 1.94 | 0.005749354 | HPSE | 82 | -2.10 | 3.65E-09 |
| RAB3B | 1270 | -1.81 | 2.45E-09 | CYP3A39 | 264 | 1.94 | 0.005749354 | PTCH2 | 81 | -1.03 | 0.028445393 |
| ERO1B | 1263 | -1.86 | 3.09E-10 | CYP3A39 | 264 | 1.94 | 0.005749354 | SKAP1 | 81 | -1.41 | 0.002332145 |
| VASH2 | 1262 | -1.03 | 0.030597398 | CYP3A39 | 264 | 1.94 | 0.005749354 | F2RL2 | 81 | -1.42 | 0.0079175 |
| CA2 | 1253 | 1.05 | 1.21E-07 | ARG2 | 263 | 1.23 | 2.06E-08 | TMEM154 | 81 | -1.38 | 2.50E-07 |
| LIF | 1243 | 1.63 | 0.00752078 | DDR2 | 263 | -1.12 | 0.000812293 | TICRR | 80 | -2.16 | 7.32E-10 |
| BCL6B | 1237 | -1.23 | 0.042300724 | C1QTNF1 | 263 | -1.33 | 0.000291865 | GHSR | 80 | -2.04 | 0.000156124 |
| CPEB2 | 1230 | 1.03 | 9.37E-07 | LDHD | 263 | 1.61 | 3.42E-14 | BLOC1S3 | 80 | 1.21 | 0.009256559 |
| PTGS2 | 1228 | 2.75 | 0.015108474 | E2F7 | 260 | -2.14 | 4.44E-14 | EXTL1 | 80 | -2.20 | 4.69E-07 |
| PTN | 1211 | -2.61 | 4.08E-12 | VRK1 | 260 | -1.57 | 3.01E-22 | PSMC3IP | 79 | -2.74 | 2.88E-13 |
| NFKBIZ | 1208 | 1.31 | 0.00132326 | JAK3 | 259 | 1.78 | 4.82E-08 | EFCAB8 | 79 | 1.68 | 0.003877492 |
| FZD4 | 1195 | 1.15 | 7.10E-12 | GJA4 | 259 | -1.63 | 0.003055724 | SSPO | 79 | 1.10 | 0.018609181 |
| ANXA10 | 1189 | -2.35 | 4.33E-07 | OXTR | 257 | -1.33 | 0.004084086 | EPS8L1 | 79 | 1.03 | 0.014722634 |
| ADAMTS1 | 1183 | 1.72 | 2.11E-10 | ANO1 | 257 | -2.82 | 4.81E-06 | PTPRC | 79 | -1.20 | 0.044220518 |
| OPCML | 1179 | -3.65 | 1.96E-07 | MMP19 | 256 | -1.05 | 0.025242478 | BRINP1 | 78 | -2.29 | 8.26E-10 |
| NNAT | 1171 | -1.91 | 2.78E-12 | CHAF1B | 256 | -1.27 | 7.32E-13 | NRK | 78 | -3.99 | 5.78E-08 |
| PLCD1 | 1168 | 1.17 | 7.20E-06 | NDC80 | 255 | -3.75 | 8.34E-26 | LEF1 | 78 | -2.18 | 0.000241468 |
| NFKB2 | 1156 | 1.03 | 1.47E-07 | E2F5 | 255 | 1.18 | 6.00E-06 | ARPP21 | 78 | -1.19 | 0.013561549 |
| GJD2 | 1153 | -2.71 | 5.21E-09 | ITGA11 | 255 | -4.02 | 5.80E-05 | FOXJ1 | 78 | 1.07 | 0.010847788 |
| NPNT | 1144 | -1.06 | 1.10E-05 | PRKAG3 | 254 | -1.46 | 9.75E-13 | PCP4 | 78 | 2.81 | 2.48E-07 |
| SFRP2 | 1143 | -3.52 | 0.000866311 | ROR1 | 254 | -2.02 | 1.08E-05 | CHADL | 77 | -1.38 | 0.002304125 |
| KITLG | 1137 | -1.06 | 0.00081594 | BICD1 | 253 | -1.43 | 2.64E-05 | MTBP | 77 | -2.99 | 7.81E-13 |
| CDH17 | 1134 | -1.60 | 6.06E-08 | SHCBP1 | 250 | -3.30 | 1.56E-18 | NPY1R | 77 | -1.55 | 0.003814953 |
| CRISPLD2 | 1134 | -1.95 | 1.20E-05 | NOVA1 | 248 | -1.35 | 2.56E-06 | RF00334 | 76 | 1.72 | 0.000661831 |
| ANGPTL2 | 1127 | -1.16 | 0.015243523 | SLC7A4 | 247 | -1.19 | 5.41E-08 | DIRAS2 | 76 | -1.99 | 4.60E-06 |
| MYC | 1119 | 1.72 | 1.12E-13 | CRYBA2 | 246 | -1.93 | 1.02E-12 | SYT14 | 76 | -1.04 | 0.001842306 |
| PSD | 1114 | -1.22 | 4.92E-06 | ASF1B | 246 | -3.65 | 4.56E-31 | TRIL | 75 | -3.19 | 1.75E-06 |
| FBLN1 | 1109 | -1.23 | 9.65E-06 | GPX7 | 246 | -1.38 | 0.002106542 | TRPC6 | 75 | -1.22 | 0.026493185 |
| SLC5A3 | 1108 | 2.70 | 2.96E-21 | SPC25 | 245 | -1.34 | 1.06E-09 | HAS1 | 75 | 1.00 | 0.014593301 |
| MCM2 | 1102 | -2.23 | 3.19E-30 | EML5 | 245 | -1.06 | 7.47E-06 | PCDH10 | 74 | -1.11 | 0.006997921 |
| ITGB3 | 1094 | 1.79 | 2.05E-12 | GGT5 | 243 | -1.78 | 0.000333322 | DOK6 | 74 | -2.85 | 5.21E-09 |
| ANGPT2 | 1079 | 2.23 | 2.86E-12 | KLHL32 | 243 | -1.50 | 3.78E-09 | HEMGN | 74 | -1.03 | 0.001031853 |

| | | | | | | | | | | | |
|---|---|---|---|---|---|---|---|---|---|---|---|
| FAM169A | 1065 | -1.03 | 3.42E-07 | CHST2 | 242 | -1.02 | 0.014023627 | C5AR1 | 73 | -1.95 | 0.00012365 |
| FBN2 | 1060 | -1.80 | 8.01E-13 | NEFL | 242 | -1.17 | 0.01515294 | ABCG2 | 73 | -1.30 | 0.000712793 |
| FLNC | 1030 | 1.24 | 0.000681621 | CDCA8 | 242 | -3.38 | 7.04E-20 | HECTD2 | 73 | -1.09 | 0.013971132 |
| RPH3AL | 1025 | -1.11 | 3.17E-06 | VDR | 242 | -2.45 | 4.38E-12 | RD3 | 72 | -2.29 | 2.68E-06 |
| LIMCH1 | 1024 | -1.12 | 2.15E-05 | CHRNB2 | 240 | -1.53 | 0.0002776 | SERTAD4 | 72 | -1.33 | 0.019729238 |
| SYT7 | 1018 | -1.26 | 4.83E-05 | CCNB2 | 239 | -3.50 | 1.40E-23 | NUP210L | 72 | 1.33 | 0.001709342 |
| BEX4 | 1012 | -1.41 | 1.04E-16 | MYT1 | 237 | -1.13 | 0.00797908 | XKR5 | 72 | -2.35 | 8.60E-09 |
| TENT5C | 1004 | -1.78 | 7.25E-19 | HMGCLL1 | 236 | -1.62 | 3.90E-06 | TMEM45A | 72 | -1.55 | 0.00014007 |
| CCDC88A | 980 | -1.27 | 8.93E-08 | MAP6 | 236 | -1.74 | 1.88E-08 | ADAMTS20 | 71 | 1.84 | 0.000152797 |
| ATP2A3 | 980 | -1.56 | 0.000400579 | SERPINA6 | 236 | 2.45 | 2.43E-05 | TRAIP | 71 | -2.00 | 2.38E-07 |
| ROS1 | 969 | -1.07 | 0.01341087 | DIAPH3 | 234 | -2.70 | 6.68E-17 | ST6GALNAC | 71 | -1.68 | 3.90E-06 |
| ADAMTS15 | 965 | -1.30 | 6.06E-06 | GAS2 | 234 | -1.40 | 0.000148256 | UNC5C | 71 | -1.95 | 2.91E-05 |
| MARC2 | 957 | 1.16 | 0.020737432 | AURKB | 233 | -1.71 | 8.85E-05 | CCR7 | 71 | -2.64 | 2.21E-07 |
| GCH1 | 954 | -1.29 | 2.15E-09 | TCF19 | 232 | -3.28 | 4.56E-31 | DUOXA1 | 71 | 1.44 | 0.0456332 |
| ADCY1 | 951 | -1.05 | 0.011738752 | CARMIL2 | 232 | -1.12 | 9.70E-07 | KCNG3 | 70 | -1.64 | 0.006665948 |
| ELMO1 | 949 | -1.34 | 0.000528456 | PPP1R3B | 232 | -1.08 | 0.000710902 | SSTR2 | 70 | -1.90 | 1.58E-05 |
| ZBTB4 | 938 | 1.08 | 1.96E-12 | SLC35G2 | 231 | -2.02 | 1.91E-09 | MYT1L | 70 | -1.35 | 0.003962842 |
| TNFRSF11A | 918 | -2.30 | 2.85E-21 | MDGA1 | 230 | -1.59 | 0.000141007 | PARPBP | 70 | -1.90 | 4.83E-06 |
| VWA5B2 | 915 | -1.84 | 6.54E-09 | GABRB3 | 230 | -1.54 | 0.000319744 | ATP2B3 | 70 | 1.53 | 8.56E-08 |
| CACNA1A | 913 | -1.58 | 5.55E-06 | RECQL4 | 227 | -1.79 | 6.94E-07 | NEIL3 | 70 | -2.64 | 2.13E-09 |
| SHISAL1 | 906 | -1.00 | 0.000250327 | BIRC5 | 227 | -1.61 | 4.55E-12 | ADAM22 | 70 | -1.76 | 2.78E-06 |
| SYT5 | 896 | -2.29 | 4.02E-19 | C1QC | 226 | -1.48 | 0.000986054 | HSD11B2 | 70 | -1.09 | 0.004663082 |
| MMP13 | 894 | 2.29 | 0.044886681 | ATAD5 | 226 | -1.26 | 1.64E-08 | MAP7D2 | 70 | -1.27 | 0.002983395 |
| PCOLCE | 892 | -1.05 | 0.000661264 | POC1A | 225 | -1.99 | 1.10E-19 | FANCL | 70 | -1.10 | 0.000237357 |
| ELK3 | 885 | -1.12 | 0.000281603 | SALL4 | 223 | 1.35 | 1.69E-06 | KCNJ6 | 69 | -1.26 | 0.019233513 |
| FLYWCH1 | 883 | 1.12 | 1.38E-08 | SERPIND1 | 223 | -1.87 | 1.73E-09 | FIGNL2 | 69 | 1.29 | 0.00143872 |
| CDC14A | 878 | 1.13 | 3.62E-12 | PASK | 222 | -1.45 | 2.34E-10 | SLC25A47 | 69 | 1.17 | 0.001582883 |
| RGS6 | 873 | 1.33 | 8.23E-08 | CILP | 221 | -3.87 | 0.000112157 | ARL9 | 69 | -1.04 | 0.008339725 |
| SLC2A8 | 865 | 1.14 | 6.20E-06 | MST1R | 221 | 1.23 | 2.03E-10 | IL6 | 69 | 2.99 | 0.000167792 |
| HAPLN4 | 858 | 1.10 | 0.000168313 | FOLR1 | 221 | -1.82 | 1.81E-08 | HTR2A | 69 | -1.88 | 7.55E-05 |
| PDE3B | 853 | -1.25 | 6.36E-09 | FOLR1 | 221 | -1.82 | 1.81E-08 | JAZF1 | 69 | -1.68 | 4.63E-05 |
| CDS1 | 849 | 1.04 | 0.000626414 | PTGIS | 220 | -3.32 | 6.49E-07 | BTBD17 | 69 | -3.48 | 6.92E-09 |
| CENPF | 847 | -2.83 | 1.57E-14 | UBASH3B | 219 | -1.56 | 9.56E-13 | PLEKHG6 | 68 | 1.36 | 0.000358597 |
| DCN | 840 | -2.03 | 2.43E-05 | TCF7L1 | 218 | 1.02 | 2.31E-05 | STK33 | 68 | -1.21 | 0.010060257 |
| TPX2 | 839 | -2.52 | 1.62E-26 | TRPS1 | 218 | -1.07 | 0.001054665 | SLC9A2 | 68 | -1.52 | 0.000342645 |
| CCDC3 | 837 | -2.15 | 6.33E-09 | IL10RA | 218 | -1.06 | 4.35E-07 | CFAP61 | 68 | -1.39 | 0.000225563 |
| TMEM223 | 827 | 1.22 | 6.00E-14 | SSTR1 | 217 | -2.64 | 1.09E-07 | GIPR | 68 | -2.23 | 3.76E-07 |
| CASR | 826 | -2.77 | 1.44E-18 | PLK1 | 217 | -2.58 | 5.89E-14 | PLEKHD1 | 68 | 1.24 | 0.011524988 |
| DTX1 | 823 | -1.66 | 2.42E-24 | NDST3 | 217 | -5.12 | 1.79E-13 | CEP72 | 68 | -2.01 | 2.89E-07 |
| PTGER4 | 815 | -1.37 | 0.000910279 | SRPX | 216 | -1.26 | 0.004204588 | TYROBP | 68 | -1.36 | 0.00367116 |
| CCNB1IP1 | 812 | 1.04 | 4.00E-14 | HEYL | 216 | -3.25 | 2.85E-08 | OGN | 67 | -1.66 | 0.04328517 |
| TPM2 | 812 | -1.48 | 0.049452441 | PLA2R1 | 215 | -2.14 | 0.00011766 | DGAT2 | 67 | 1.68 | 0.000507582 |
| CIP2A | 810 | -1.24 | 3.23E-16 | GINS4 | 214 | -1.18 | 1.14E-07 | PRLHR | 67 | -1.73 | 0.000206979 |
| KCNK16 | 806 | -4.45 | 3.43E-12 | ETV7 | 214 | 1.03 | 2.36E-06 | HHIPL2 | 67 | -2.48 | 0.000415968 |
| PAPSS2 | 805 | -1.48 | 1.27E-05 | TMPRSS11F | 213 | 2.93 | 1.53E-07 | RBP1 | 67 | -2.84 | 1.93E-07 |
| ARHGEF9 | 803 | -1.38 | 5.67E-08 | MFAP2 | 213 | -3.14 | 4.19E-06 | DIRAS1 | 67 | -1.51 | 0.006195372 |
| SOWAHA | 802 | -1.42 | 4.67E-05 | CPAMD8 | 211 | -1.57 | 0.012957248 | GFRA3 | 66 | -2.72 | 0.006047769 |
| DCXR | 798 | 1.06 | 3.00E-07 | CACNB2 | 210 | -1.14 | 0.000276344 | LYVE1 | 65 | -2.39 | 1.50E-05 |
| PIM3 | 797 | 1.26 | 1.59E-16 | FBLN5 | 210 | -1.23 | 0.011903336 | SHISA7 | 65 | -1.35 | 0.00122161 |

| | | | | | | | | | | | |
|---|---|---|---|---|---|---|---|---|---|---|---|
| TSPYL5 | 796 | -1.03 | 3.84E-06 | FAM162B | 210 | -1.24 | 0.012155717 | NKD1 | 65 | -2.11 | 0.003443352 |
| FKBP10 | 780 | -1.03 | 0.02211151 | IFNLR1 | 208 | 1.69 | 9.80E-12 | CCSER1 | 65 | 1.11 | 0.00044914 |
| MAFB | 780 | -2.43 | 1.54E-12 | CCNF | 207 | -1.84 | 1.19E-12 | CENPL | 64 | -1.89 | 4.03E-05 |
| SNAP25 | 777 | -1.29 | 1.36E-07 | KIF14 | 207 | -3.15 | 1.53E-14 | PTCHD1 | 64 | 1.13 | 0.023350976 |
| SORD | 776 | 1.25 | 0.003590416 | CENPA | 206 | -3.90 | 5.67E-21 | COL28A1 | 63 | -2.11 | 1.90E-05 |
| UHRF1 | 776 | -2.00 | 5.83E-24 | P4HA3 | 206 | -1.57 | 0.001151391 | YDJC | 63 | 1.07 | 0.00260513 |
| GTF2IRD1 | 769 | 1.11 | 2.33E-08 | NOX4 | 204 | -1.23 | 0.000700235 | CD37 | 63 | 1.67 | 0.001538919 |
| MDFIC | 752 | -1.43 | 1.20E-06 | KCNA4 | 204 | -3.67 | 1.32E-12 | C17orf53 | 63 | -1.38 | 0.000131654 |
| RGS16 | 751 | -1.88 | 6.45E-13 | GALNT13 | 204 | -2.33 | 1.33E-09 | ZNF550 | 63 | 1.30 | 0.001118647 |
| EDNRA | 747 | -1.82 | 0.009083873 | BARD1 | 203 | -1.49 | 4.04E-07 | TRPM8 | 63 | 1.19 | 0.001833687 |
| PHLDA2 | 744 | 1.23 | 3.48E-15 | AMPH | 200 | -1.58 | 1.46E-05 | KCTD7 | 63 | 1.27 | 5.52E-06 |
| GRIK1 | 744 | -2.78 | 4.34E-21 | MMS22L | 200 | -2.03 | 2.65E-14 | SHAS2 | 62 | -2.88 | 1.10E-05 |
| MCM3 | 739 | -1.78 | 2.84E-13 | SCEL | 199 | 1.14 | 0.000313121 | REC8 | 62 | -1.85 | 1.20E-05 |
| ADGRA2 | 737 | -1.26 | 0.026279667 | CENPN | 199 | -1.77 | 1.38E-12 | FANCB | 62 | -1.15 | 0.000962556 |
| ELP4 | 731 | -1.52 | 1.03E-07 | MGP | 199 | -3.88 | 4.40E-06 | CYTIP | 61 | -1.59 | 0.036762107 |
| ATAD2 | 731 | -1.44 | 4.36E-18 | MMP16 | 198 | -1.21 | 0.000772682 | FCER1G | 61 | -1.35 | 0.028296857 |
| NOL4 | 729 | -2.10 | 1.03E-08 | KCNJ5 | 198 | -1.46 | 0.005472548 | TCP11 | 61 | 1.67 | 0.013217273 |
| SLC2A2 | 721 | -2.70 | 4.65E-08 | SIX4 | 197 | 1.54 | 4.40E-06 | DNAH5 | 61 | -1.19 | 0.013862075 |
| FAM20A | 716 | -1.77 | 5.17E-10 | DUSP9 | 196 | -1.28 | 2.44E-05 | IRX1 | 61 | -2.39 | 1.02E-08 |
| RACGAP1 | 714 | -1.62 | 5.11E-16 | CDH4 | 195 | -1.75 | 0.000124456 | TMEFF2 | 60 | -2.15 | 1.57E-05 |
| ABCC9 | 713 | -1.69 | 7.93E-05 | ADCYAP1R1 | 195 | -1.37 | 0.000471073 | GRM7 | 60 | -2.36 | 9.99E-05 |
| MEGF11 | 712 | -3.49 | 1.40E-07 | SNAI1 | 195 | -1.57 | 0.000544445 | GBA3 | 59 | -1.96 | 0.029530093 |
| SELENOM | 711 | -1.16 | 6.31E-07 | DAAM2 | 194 | -1.17 | 0.0127953 | TOX2 | 59 | 1.76 | 5.90E-05 |
| OSBPL10 | 710 | 1.18 | 0.00131852 | RFX2 | 194 | -1.43 | 0.000186046 | FREM1 | 59 | -2.44 | 0.001108352 |
| MRPS6 | 709 | 1.10 | 1.27E-16 | PIR | 193 | 1.12 | 6.04E-06 | SVOP | 58 | -1.08 | 0.028145973 |
| ISL1 | 708 | -2.28 | 4.52E-08 | PRLR | 192 | -2.43 | 4.19E-13 | RNF150 | 58 | -1.02 | 0.031607365 |
| KLK1 | 703 | -3.67 | 6.34E-06 | OGDHL | 191 | -1.64 | 1.31E-08 | MMACHC | 58 | 1.00 | 0.001555345 |
| ROBO1 | 700 | -1.32 | 0.00280632 | NAP1L3 | 191 | -1.33 | 0.000436185 | TNFRSF9 | 57 | -1.81 | 0.025015319 |
| SH3BGRL | 697 | -1.40 | 2.91E-08 | PRKG1 | 191 | -2.45 | 3.64E-08 | SHH | 57 | 1.52 | 0.041160577 |
| CACNA2D2 | 689 | -2.22 | 5.78E-07 | PCDH9 | 191 | -1.13 | 1.32E-05 | SMOC2 | 57 | -2.26 | 0.001792299 |
| ECT2 | 687 | -1.14 | 8.53E-11 | G6PC2 | 190 | -7.74 | 3.90E-06 | RAC2 | 57 | 1.64 | 0.037643574 |
| SLC6A7 | 677 | 1.21 | 0.004369084 | SCUBE3 | 189 | -1.27 | 0.011261971 | AARD | 57 | -2.34 | 0.002221894 |
| ANLN | 665 | -1.31 | 6.19E-06 | NEGR1 | 189 | -1.63 | 9.93E-07 | ZNF792 | 57 | 1.08 | 0.015003239 |
| BEX1 | 662 | -1.46 | 1.98E-19 | SERPINF1 | 189 | -2.03 | 0.00636327 | WSCD2 | 56 | -1.62 | 0.031051172 |
| CKAP2 | 660 | -2.51 | 8.63E-16 | AREG | 189 | 1.50 | 0.009449633 | RNF39 | 56 | 1.25 | 0.004663082 |
| KIF11 | 646 | -2.16 | 1.35E-16 | CENPT | 187 | -3.14 | 2.54E-24 | ADAMTS3 | 56 | -1.46 | 0.02273582 |
| CLSTN3 | 643 | -1.56 | 2.60E-07 | HEY1 | 187 | -1.16 | 0.02695452 | ATP2B2 | 56 | -1.09 | 0.010958717 |
| RBPJL | 640 | -2.50 | 0.023201555 | PPP1R3G | 186 | 1.04 | 0.0011791 | TMEM117 | 56 | -1.59 | 0.000764288 |
| SLC30A8 | 638 | -3.69 | 3.91E-14 | ORC1 | 184 | -2.96 | 5.99E-19 | SLC8A3 | 56 | -1.67 | 0.001930826 |
| NCAPD3 | 636 | -1.36 | 3.02E-18 | IRAK3 | 183 | 1.99 | 2.01E-09 | LRRC17 | 56 | -2.21 | 0.000449321 |
| MYL9 | 629 | 1.09 | 0.027492259 | C1QA | 183 | -1.44 | 0.002378304 | VEGFD | 56 | -2.76 | 3.36E-09 |
| GALNT18 | 626 | -1.03 | 0.000539967 | ITGA8 | 182 | -3.11 | 7.75E-06 | LVRN | 56 | -1.52 | 0.027936402 |
| RENBP | 622 | 1.24 | 3.42E-14 | CKAP2L | 181 | -2.51 | 8.52E-14 | ATP8B3 | 56 | 2.01 | 0.00084419 |
| LSAMP | 622 | -1.06 | 7.28E-08 | ADRA2A | 180 | -1.64 | 0.001511852 | BMP5 | 55 | -1.74 | 0.043487739 |
| DPP4 | 620 | -1.81 | 8.25E-17 | FOXM1 | 180 | -2.73 | 1.45E-14 | TIMP4 | 55 | -1.37 | 0.001266445 |
| WNT5A | 619 | -1.30 | 0.003751764 | SYNDIG1L | 180 | -1.49 | 0.002144044 | CIDEA | 54 | 1.62 | 0.007109185 |
| GDF15 | 613 | 2.06 | 1.71E-06 | FAM198B | 180 | -1.98 | 0.000128794 | TCF21 | 54 | -1.68 | 0.006108876 |
| P2RY14 | 610 | -2.96 | 4.17E-17 | GPR158 | 179 | -1.78 | 8.59E-08 | GABRR2 | 54 | -1.57 | 0.017162077 |
| RTL5 | 606 | -1.23 | 1.30E-06 | ADAM33 | 179 | -1.25 | 0.009197168 | ZNF385D | 54 | -1.65 | 0.0168998 |

| | | | | | | | | | | | |
|---|---|---|---|---|---|---|---|---|---|---|---|
| ITPRIPL1 | 603 | 1.21 | 9.86E-14 | CALB1 | 178 | -3.45 | 4.36E-08 | TMIE | 54 | -2.66 | 7.26E-12 |
| CHST9 | 603 | -1.82 | 7.30E-09 | ADGRA1 | 178 | 1.31 | 0.000259971 | COCH | 54 | -2.09 | 0.047752248 |
| ESRRA | 602 | 1.17 | 3.51E-10 | PTPRD | 178 | -1.67 | 0.005029138 | SIDT1 | 53 | -2.79 | 0.000151941 |
| ROBO2 | 602 | -3.10 | 4.61E-21 | SLCO4A1 | 178 | 1.00 | 9.90E-05 | DMRT3 | 53 | -1.71 | 0.015227744 |
| MBOAT1 | 601 | 1.11 | 5.37E-11 | MARCH4 | 177 | -3.22 | 5.12E-13 | RAB39A | 53 | -1.84 | 3.79E-06 |
| FHOD3 | 596 | -1.28 | 1.17E-12 | CORIN | 176 | -3.64 | 7.22E-08 | SFTPB | 53 | -3.01 | 0.030733945 |
| RUNDC3A | 594 | -1.10 | 1.46E-05 | THBS4 | 175 | -3.50 | 5.05E-05 | SLC22A18 | 53 | 1.28 | 0.016967335 |
| ITIH4 | 591 | -1.61 | 0.000117109 | IQSEC3 | 175 | -1.57 | 8.60E-06 | ABCA4 | 52 | 1.50 | 0.004987044 |
| CDKN2C | 587 | -1.60 | 4.45E-11 | POLQ | 175 | -2.65 | 7.70E-15 | GPR83 | 52 | -1.80 | 0.042837526 |
| ZC3H12A | 584 | 1.40 | 0.001231129 | RAD51AP1 | 174 | -1.71 | 1.57E-15 | NKX2-3 | 51 | -1.86 | 0.000385529 |
| MAFF | 581 | 2.23 | 3.12E-21 | USP13 | 173 | -1.07 | 0.013353808 | GLI2 | 51 | -2.87 | 1.56E-05 |
| NCAPG2 | 579 | -1.40 | 7.48E-14 | ARHGEF39 | 172 | -1.58 | 2.55E-08 | PRR5L | 50 | -1.50 | 0.000902149 |
| ACTA2 | 577 | -2.38 | 0.036157429 | LRRC10B | 172 | -1.09 | 0.002213132 | CEP57L1 | 50 | -1.55 | 0.000494108 |
| PCLAF | 577 | -3.74 | 2.14E-50 | WISP1 | 172 | -2.22 | 0.002711061 | STOX1 | 50 | -1.13 | 0.001527958 |
| UNC5A | 576 | -1.65 | 1.79E-07 | PRR11 | 171 | -2.75 | 2.13E-17 | FAAP24 | 50 | -1.53 | 0.001048094 |
| COX7A1 | 576 | 1.06 | 1.06E-09 | ACVR1C | 171 | -3.69 | 1.87E-17 | MAFA | 50 | -1.87 | 0.031294365 |
| S100A14 | 574 | 1.04 | 0.00042221 | RAB40B | 171 | -1.25 | 2.06E-05 | SLC11A1 | 50 | -1.00 | 0.044471426 |
| SGPP2 | 574 | 1.54 | 4.21E-05 | AMBP | 170 | -1.34 | 0.009005139 | | | | |
| PENK | 569 | -2.70 | 7.78E-12 | ARX | 170 | -1.64 | 0.002472531 | | | | |
| CXCL12 | 567 | -1.09 | 6.86E-06 | SERPINB9 | 169 | -1.02 | 0.022511712 | | | | |
| STMN3 | 562 | -1.40 | 3.71E-05 | TROAP | 169 | -2.87 | 5.65E-16 | | | | |
| UNC80 | 561 | -2.31 | 3.52E-10 | PLPPR4 | 168 | -2.64 | 2.85E-09 | | | | |
| SGCB | 560 | -1.03 | 0.001514725 | DCX | 168 | -1.58 | 0.00551381 | | | | |
| LARGE2 | 558 | 1.06 | 7.70E-06 | EVA1C | 167 | 1.76 | 6.19E-06 | | | | |
| PXDC1 | 557 | 1.03 | 0.009461968 | GADD45G | 166 | -1.67 | 1.77E-07 | | | | |
| SLC7A14 | 556 | -2.76 | 3.63E-19 | SULT1C4 | 165 | -3.24 | 1.31E-10 | | | | |
| KIF20B | 552 | -1.04 | 1.53E-10 | | | | | | | | |
| KCNH6 | 551 | -2.01 | 1.24E-09 | | | | | | | | |
| EID1 | 549 | -1.02 | 0.006999657 | | | | | | | | |

ESM Table 6: DEGs in NICCs isolated from 4 days old animals following culture with SB431542

| Gene | base Mean | log2FC | p adjusted | Gene | base Mean | log2FC | p adjusted | Gene | base Mean | log2FC | p adjusted |
|---|---|---|---|---|---|---|---|---|---|---|---|
| MMP7 | 39682 | -1.36 | 0.035276836 | LOX | 528 | -1.63 | 0.002228017 | GUCA2B | 151 | 3.36 | 1.12E-09 |
| CFD | 21617 | 1.27 | 0.009731162 | LDLR | 491 | 1.29 | 0.002268006 | KCNA6 | 150 | -2.11 | 0.002618947 |
| FN1 | 21369 | -2.57 | 0.000198704 | RAB9B | 489 | -1.03 | 0.000744777 | IRF8 | 150 | 1.35 | 0.026840531 |
| ANXA4 | 15152 | 1.03 | 2.33E-05 | SULF1 | 476 | -1.87 | 0.000963627 | DAB2 | 150 | -1.26 | 0.000883438 |
| DIRAS3 | 13930 | -1.17 | 0.000199168 | ESPN | 468 | 1.64 | 0.000763767 | FMOD | 149 | -1.32 | 0.008065442 |
| OLFML2A | 8352 | -1.32 | 0.000993603 | LRRC32 | 465 | -1.79 | 0.013486796 | SOX5 | 148 | -1.00 | 0.005558204 |
| NUPR1 | 7448 | 1.26 | 0.004782783 | PNLIPRP1 | 458 | 1.61 | 2.61E-11 | SLPI | 148 | -1.84 | 0.000102646 |
| COL5A2 | 7390 | -1.21 | 0.007800906 | CTSL | 457 | -1.14 | 0.000300205 | TNFAIP6 | 145 | -2.82 | 7.78E-08 |
| PKHD1 | 6963 | -1.61 | 3.91E-14 | AFP | 457 | 2.47 | 3.73E-05 | FOXS1 | 145 | -1.48 | 0.002618947 |
| IL1R1 | 6724 | -1.17 | 1.24E-05 | FBLN7 | 455 | 1.32 | 7.14E-06 | NEURL1B | 144 | 1.62 | 0.000364071 |
| THBS1 | 6466 | -2.17 | 6.08E-29 | C1orf115 | 455 | 1.42 | 5.62E-08 | GYPC | 143 | 1.42 | 5.86E-07 |
| SPHK1 | 6415 | 1.38 | 3.99E-20 | EPHA4 | 450 | -1.17 | 1.34E-05 | CTHRC1 | 142 | -1.45 | 0.021844979 |
| COL5A1 | 6164 | -1.44 | 0.019515584 | ACTA1 | 442 | -1.91 | 0.000214448 | MT3 | 139 | 2.31 | 0.023155338 |
| SLC7A8 | 5724 | -1.29 | 0.000272898 | C1S | 442 | 1.50 | 0.00210357 | C11orf96 | 138 | 1.06 | 0.036567994 |
| PMEPA1 | 5323 | -3.16 | 2.09E-35 | RNASE4 | 427 | 1.31 | 0.007561183 | GPR39 | 138 | -1.12 | 0.000514602 |
| CYR61 | 4739 | -1.00 | 0.033922162 | CCL2 | 424 | -1.43 | 4.94E-05 | EFS | 137 | -1.13 | 0.016246885 |
| TNC | 4663 | -3.15 | 9.72E-08 | CLMP | 404 | -2.37 | 7.37E-11 | UABP-2 | 136 | -1.97 | 0.005431187 |
| FSTL1 | 4364 | -1.53 | 0.008837579 | TGFB2 | 391 | -1.01 | 0.000398511 | CBLN4 | 136 | -3.47 | 1.07E-05 |
| COL12A1 | 4350 | -2.19 | 5.21E-13 | CYGB | 373 | 2.67 | 0.000105408 | SAMD11 | 136 | -1.31 | 0.008507162 |
| PTK7 | 4202 | -1.09 | 1.76E-06 | UCN3 | 351 | -1.04 | 0.047176314 | EBF1 | 132 | 2.13 | 1.58E-05 |
| BMF | 3665 | -1.65 | 5.99E-14 | HSF4 | 350 | 1.53 | 0.023458296 | EDIL3 | 132 | -3.37 | 1.86E-06 |
| RGS4 | 3614 | -1.08 | 0.001492554 | RYR2 | 340 | -1.14 | 0.012380366 | BMPR1B | 130 | -2.99 | 3.48E-07 |
| ABCC8 | 3418 | -1.08 | 0.038379868 | FAM124A | 326 | -1.11 | 6.14E-05 | VGLL1 | 129 | -1.08 | 0.000416422 |
| LTBP1 | 3406 | -1.50 | 0.000754091 | PLXNA4 | 322 | -2.12 | 0.001050457 | TRPC4 | 127 | -1.32 | 7.16E-08 |
| NEDD9 | 3248 | -1.15 | 0.000458717 | EDN1 | 322 | -1.80 | 5.69E-09 | SLC16A9 | 126 | -2.09 | 0.000120851 |
| COL16A1 | 3108 | -2.29 | 5.76E-13 | PDGFRA | 321 | -1.28 | 0.018840092 | GDNF | 124 | -1.84 | 0.00029544 |
| C14orf132 | 3099 | -1.08 | 4.89E-09 | KLF15 | 321 | 1.11 | 3.99E-05 | LIPG | 122 | -2.29 | 4.91E-06 |
| ANTXR1 | 2810 | -1.20 | 0.004919819 | ADCY7 | 315 | -1.67 | 7.19E-15 | NFATC1 | 122 | -1.11 | 0.012853208 |
| SFRP1 | 2741 | -2.26 | 0.003729171 | ITGA7 | 304 | 1.68 | 0.003262811 | ASPN | 122 | -3.49 | 0.001245145 |
| SLC1A1 | 2520 | -1.09 | 0.01272644 | P2RY1 | 304 | -1.21 | 0.027259573 | PPP1R14A | 118 | 1.14 | 0.024197387 |
| LOXL2 | 2339 | -1.42 | 8.60E-05 | ACSL5 | 302 | 1.03 | 0.008000803 | TM7SF2 | 117 | -1.34 | 1.00E-05 |
| PLA2G1B | 2335 | 2.41 | 8.77E-05 | TNFAIP8L1 | 302 | 1.11 | 0.02107044 | LMCD1 | 117 | -1.14 | 0.046799531 |
| ENPP2 | 2237 | -1.54 | 0.032247923 | BMP4 | 298 | -1.12 | 2.82E-07 | S100A12 | 115 | -6.09 | 1.01E-10 |
| PHGDH | 2047 | 1.13 | 2.27E-24 | CAVIN3 | 296 | 1.37 | 0.009636192 | CES3 | 114 | 1.49 | 0.000654592 |
| CP | 2042 | -1.67 | 0.001089954 | NPC1L1 | 292 | 1.29 | 0.014376763 | RUNX2 | 110 | -1.45 | 0.048947126 |
| TRH | 1948 | -7.44 | 1.11E-45 | COL8A1 | 291 | -5.90 | 1.78E-08 | RBP5 | 109 | 2.27 | 0.008837579 |
| ENTPD5 | 1818 | 1.33 | 0.000327368 | CLMN | 288 | -1.64 | 7.45E-08 | NT5E | 108 | -2.81 | 0.000117717 |
| LUM | 1771 | -2.33 | 1.19E-06 | CHST11 | 286 | -1.55 | 0.000247378 | SRPX2 | 108 | -3.03 | 1.26E-08 |
| THBS2 | 1769 | -4.40 | 9.80E-06 | MASP2 | 285 | 1.22 | 0.036871886 | ALOX15 | 107 | -1.26 | 0.011367558 |
| ECM1 | 1742 | -1.77 | 2.62E-05 | ITGA1 | 284 | -1.85 | 2.25E-08 | ALOX15 | 107 | -1.26 | 0.011367558 |
| IL11 | 1727 | -2.62 | 5.21E-13 | FNDC1 | 282 | -1.78 | 0.002157441 | GCNT1 | 106 | -1.70 | 0.014295166 |
| TINAGL1 | 1710 | 1.50 | 0.000193293 | PEAR1 | 281 | 1.97 | 1.99E-05 | PLA1A | 106 | 1.10 | 0.000771802 |
| ENG | 1694 | -1.73 | 5.28E-05 | DDIT3 | 278 | 1.02 | 5.25E-05 | SCN4B | 105 | -4.61 | 5.16E-14 |
| PLAUR | 1677 | -1.10 | 2.99E-07 | PLA2G4A | 271 | -1.28 | 0.003262811 | KCNV1 | 105 | -1.74 | 0.000172688 |
| NTN4 | 1620 | -1.19 | 6.13E-13 | ADAMTS6 | 261 | -1.64 | 1.30E-10 | CARD11 | 104 | -1.58 | 0.002106542 |
| VCAN | 1585 | -1.39 | 9.85E-05 | RHPN1 | 258 | -1.30 | 0.00165217 | PLB1 | 104 | -1.22 | 0.027109274 |
| VCAN | 1585 | -1.39 | 9.85E-05 | OXTR | 257 | -1.26 | 0.021836615 | LOXL1 | 103 | -2.39 | 0.00468944 |

| | | | | | | | | | | | |
|---|---|---|---|---|---|---|---|---|---|---|---|
| ATF5 | 1568 | 1.19 | 2.22E-07 | ITGA11 | 255 | -2.59 | 0.037339258 | BCAS1 | 103 | 2.09 | 5.33E-10 |
| SFRP5 | 1550 | -2.02 | 0.006593964 | PRND | 244 | 2.68 | 0.019002953 | NEXN | 102 | -2.95 | 6.19E-11 |
| LPL | 1544 | 1.61 | 4.11E-05 | GGT5 | 243 | -1.49 | 0.011669961 | ARC | 102 | 1.16 | 0.000511845 |
| RAB31 | 1543 | -1.26 | 0.000568688 | NEFL | 242 | -1.76 | 0.000653904 | LGALS2 | 102 | 3.00 | 6.71E-07 |
| BDKRB2 | 1535 | -1.95 | 1.83E-21 | VDR | 242 | -1.64 | 2.80E-05 | COL11A1 | 100 | -1.95 | 0.008773164 |
| CXCL14 | 1527 | -3.65 | 1.54E-07 | SMAD7 | 240 | -2.86 | 1.90E-17 | MMP28 | 97 | -1.84 | 0.005830279 |
| PLVAP | 1502 | -1.50 | 0.000572775 | SERPINA6 | 236 | 2.21 | 0.000745241 | ITPKA | 96 | 1.42 | 0.01637757 |
| TGFBI | 1466 | -1.22 | 0.000953076 | LDB2 | 234 | 1.42 | 0.028915185 | LYPD8 | 94 | 2.51 | 5.57E-06 |
| MRC2 | 1453 | -3.50 | 4.57E-06 | OLFML2B | 228 | -2.40 | 0.000492723 | NYAP2 | 94 | -1.33 | 0.039101955 |
| TACSTD2 | 1444 | 1.14 | 3.58E-10 | SLA-DQB1 | 225 | 1.33 | 0.043184419 | IL2RA | 91 | -1.34 | 0.008435952 |
| FSTL3 | 1342 | -1.38 | 1.70E-12 | APOLD1 | 222 | 1.56 | 0.000754091 | C4BPB | 89 | -1.20 | 0.006699821 |
| SKIL | 1331 | -2.30 | 8.14E-35 | CILP | 221 | -4.60 | 3.20E-05 | MSC | 89 | -2.37 | 4.50E-12 |
| OXCT1 | 1290 | 1.01 | 4.55E-16 | DEPP1 | 221 | 1.54 | 0.003950258 | BEGAIN | 88 | -1.31 | 0.02576501 |
| VASH2 | 1262 | -1.94 | 6.26E-05 | GUCA2A | 221 | 4.20 | 3.41E-05 | COL8A2 | 84 | -5.81 | 3.21E-06 |
| CA2 | 1253 | 1.44 | 5.03E-13 | LTBP2 | 219 | -1.55 | 0.00363448 | CAMK1G | 84 | -1.27 | 0.008773164 |
| VWF | 1219 | 1.08 | 0.034320026 | TRPS1 | 218 | -1.04 | 0.005461917 | S1PR5 | 82 | 1.60 | 0.000312144 |
| LAMA3 | 1189 | -1.21 | 2.81E-20 | BDKRB1 | 218 | -2.81 | 9.20E-15 | BCL2L15 | 81 | 1.13 | 0.000664274 |
| SERPINE2 | 1183 | -1.65 | 3.06E-05 | NDST3 | 217 | -1.64 | 0.03504068 | STAC | 80 | -2.98 | 2.87E-06 |
| GSTA2 | 1129 | 2.33 | 4.91E-07 | PLA2R1 | 215 | -2.23 | 0.000269902 | BLOC1S3 | 80 | 1.20 | 0.03041282 |
| GSTA2 | 1129 | 2.33 | 4.91E-07 | MFAP2 | 213 | -4.59 | 1.50E-10 | NHS | 79 | -1.80 | 0.010813307 |
| GSTA2 | 1129 | 2.33 | 4.91E-07 | HGF | 211 | -1.42 | 0.000793024 | LEF1 | 78 | -1.76 | 0.011781151 |
| ANGPTL2 | 1127 | -2.31 | 1.45E-06 | CPAMD8 | 211 | 1.97 | 0.003234632 | BNC2 | 77 | -1.78 | 0.037591422 |
| PALLD | 1122 | -1.10 | 0.008742252 | NAV3 | 211 | -1.28 | 0.034522343 | TRIL | 75 | -2.34 | 0.001710407 |
| FBN2 | 1060 | -1.10 | 0.00012795 | FILIP1 | 210 | 1.38 | 0.020236534 | RAB7B | 75 | 2.44 | 1.26E-08 |
| PPP1R16B | 1042 | 1.26 | 0.008354745 | EPHB6 | 210 | -1.30 | 5.17E-05 | TRPC6 | 75 | -1.31 | 0.047264181 |
| DCLK1 | 949 | -1.18 | 0.039101955 | ARSI | 210 | -1.69 | 0.035276836 | CLEC2B | 73 | 1.47 | 0.002027844 |
| ELMO1 | 949 | -1.69 | 5.16E-05 | P4HA3 | 206 | -2.01 | 0.000123844 | CRYBG2 | 73 | 1.81 | 6.30E-05 |
| | 890 | -1.35 | 2.87E-05 | NOX4 | 204 | -1.60 | 4.63E-05 | DIO2 | 72 | -3.38 | 4.11E-14 |
| ELK3 | 885 | -1.36 | 4.84E-05 | DISP3 | 199 | -1.25 | 0.027117052 | TMEM45A | 72 | -1.08 | 0.024542782 |
| MEGF6 | 874 | -1.01 | 6.57E-05 | COX6B2 | 197 | -1.47 | 6.19E-11 | ACE2 | 71 | 1.77 | 5.33E-06 |
| CEMIP | 834 | -2.45 | 5.41E-07 | SNAI1 | 195 | -1.79 | 0.000313776 | UNC5C | 71 | -1.21 | 0.031814089 |
| DTX1 | 823 | -1.10 | 1.51E-10 | PTP4A3 | 191 | 1.25 | 0.016755396 | CCR7 | 71 | -2.09 | 0.00017681 |
| GRIP1 | 785 | -1.00 | 1.08E-15 | SCUBE3 | 189 | -1.83 | 0.000754091 | SSUH2 | 71 | 1.84 | 0.01637757 |
| FKBP10 | 780 | -1.10 | 0.040370049 | FAM198B | 180 | -1.29 | 0.044817348 | IL34 | 70 | 1.49 | 0.004179728 |
| INHBA | 773 | -3.70 | 1.51E-45 | ZNF469 | 177 | -1.02 | 0.002031613 | PHF24 | 69 | -1.05 | 0.013128072 |
| GADD45B | 767 | -1.36 | 0.005021651 | MARCH4 | 177 | -1.37 | 0.00762695 | SMPD2 | 69 | 1.14 | 0.038718259 |
| RGS16 | 751 | -1.40 | 9.63E-07 | MYCL | 176 | -1.11 | 0.012299152 | HTR2A | 69 | -2.47 | 2.31E-06 |
| PHLDA2 | 744 | 1.01 | 1.61E-09 | HLA-DRA | 176 | 2.06 | 0.015918259 | EHD3 | 68 | -1.01 | 0.018308176 |
| SPSB1 | 740 | -1.41 | 3.92E-09 | FZD2 | 173 | -1.13 | 0.000123462 | RBP1 | 67 | -1.61 | 0.012038034 |
| MEGF11 | 712 | -2.57 | 0.000678893 | PTGS1 | 173 | -2.43 | 2.13E-05 | S100A9 | 66 | -3.90 | 9.85E-05 |
| GRB7 | 667 | -1.00 | 3.96E-05 | WISP1 | 172 | -2.68 | 0.001017358 | SHAS2 | 62 | -2.88 | 6.26E-05 |
| EGF | 637 | 1.40 | 0.009690906 | IRAK2 | 172 | 1.23 | 0.002018411 | OTOP3 | 61 | 1.11 | 0.049390041 |
| MYL9 | 629 | -1.27 | 0.028088014 | AMBP | 170 | 1.88 | 0.000291598 | CYTIP | 61 | -2.81 | 0.000557332 |
| WNT5A | 619 | -3.49 | 5.20E-16 | KANK3 | 168 | 1.11 | 0.011941447 | NAAA | 58 | -1.39 | 0.000175945 |
| CHST1 | 615 | 1.48 | 0.000283294 | FOXF1 | 166 | -1.12 | 0.000586638 | PADI1 | 57 | -2.41 | 0.007147303 |
| C1R | 597 | 1.65 | 5.58E-07 | LSP1 | 164 | -1.54 | 0.040377346 | TNFRSF9 | 57 | -2.60 | 0.003948809 |
| FHOD3 | 596 | -1.25 | 2.40E-11 | SRGN | 163 | -1.80 | 0.00058275 | TLR2 | 57 | -1.90 | 0.000116677 |
| HABP2 | 594 | -1.49 | 2.08E-12 | SLITRK6 | 162 | -2.35 | 0.001103736 | NRROS | 56 | 1.46 | 0.0149231 |
| AEBP1 | 578 | -2.35 | 0.00016883 | GKN3 | 161 | -1.67 | 0.015146958 | ALPL | 56 | -2.37 | 0.007322529 |

| | | | |
|---|---|---|---|
| SERPINE1 | 576 | -1.91 | 9.58E-05 |
| FABP5 | 569 | 1.36 | 0.013211519 |
| SLC7A14 | 556 | -1.24 | 0.000491299 |
| KCNH6 | 551 | -1.24 | 0.001317038 |
| CPLX1 | 546 | 1.17 | 7.98E-06 |
| INHBB | 535 | 1.32 | 0.001050457 |
| CYS1 | 531 | -1.17 | 2.89E-16 |

| | | | |
|---|---|---|---|
| LYPD1 | 160 | -1.01 | 9.27E-05 |
| SNED1 | 160 | 1.43 | 1.05E-06 |
| RASL12 | 160 | 1.66 | 0.008773164 |
| KCNK3 | 159 | -1.46 | 0.024248803 |
| DUSP2 | 157 | 1.14 | 0.002223723 |
| CPED1 | 156 | -1.31 | 0.038532699 |
| GPX8 | 155 | -1.07 | 0.047493418 |
| C21orf62 | 153 | -1.27 | 0.03291443 |

| | | | |
|---|---|---|---|
| LVRN | 56 | -2.73 | 0.000295003 |
| CUX2 | 54 | -1.94 | 0.009367197 |
| CNTFR | 54 | 1.38 | 0.019720629 |
| SIDT1 | 53 | -1.91 | 0.026601761 |
| NHSL2 | 53 | 1.28 | 0.002230638 |
| SFTPB | 53 | 3.42 | 8.43E-08 |
| GLI2 | 51 | -1.80 | 0.02044867 |
| ABI3BP | 50 | 2.58 | 4.43E-05 |
| SLC11A1 | 50 | -1.19 | 0.044368604 |

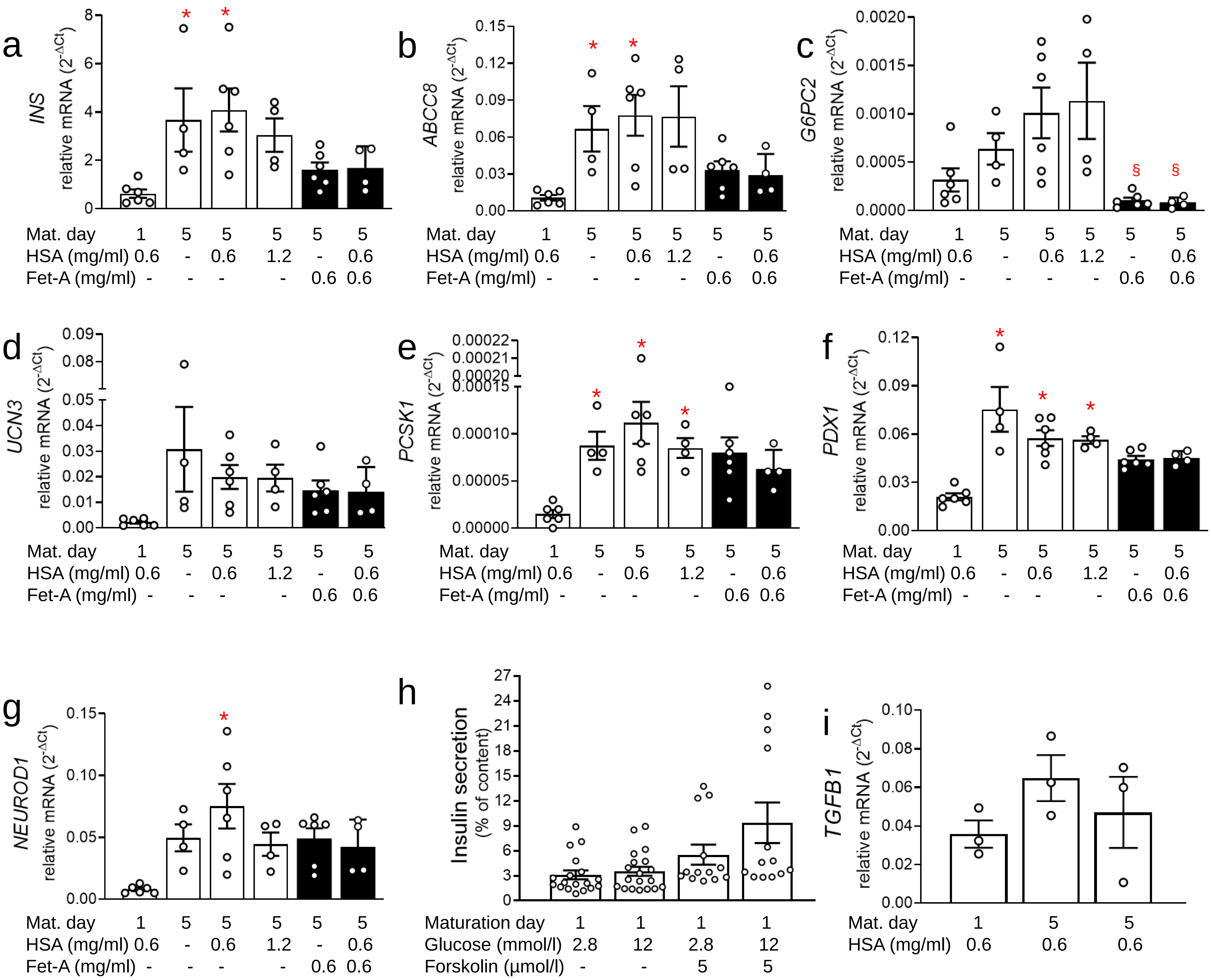


**ESM Figure 1 Functional maturation of NICCs does not depend on HSA.** (a-g, i) NICCs were maturated for 5d in HSA-free medium or in medium supplemented with human serum albumin (HSA) or fetuin-A (Fet-A) at different concentrations as indicated and described under Methods. (a-g, i) Relative mRNA levels (ΔCt against RPS13) assessed by RT-PCR of (a) *INS* (insulin), (b) *ABCC8*, (c) *G6PC2*, (d) *UCN3*, (e) *PCSK1*, (f) *PDX1*, (g) *NEUROD1* and (i) *TGFB1* and expressed as mean ± SEM of (a-g) n = 6 and (i) n = 3 independent NICCs preparations (h) Insulin secretion expressed as % of insulin content of immature NICCs (HSA/d1) cultured in standard medium and presented as mean ± SEM of n = 13-19 replicates out of 6 independent NICCs preparations. $p < 0.05$ * significant vs maturation d1; § vs respective maturation d5.

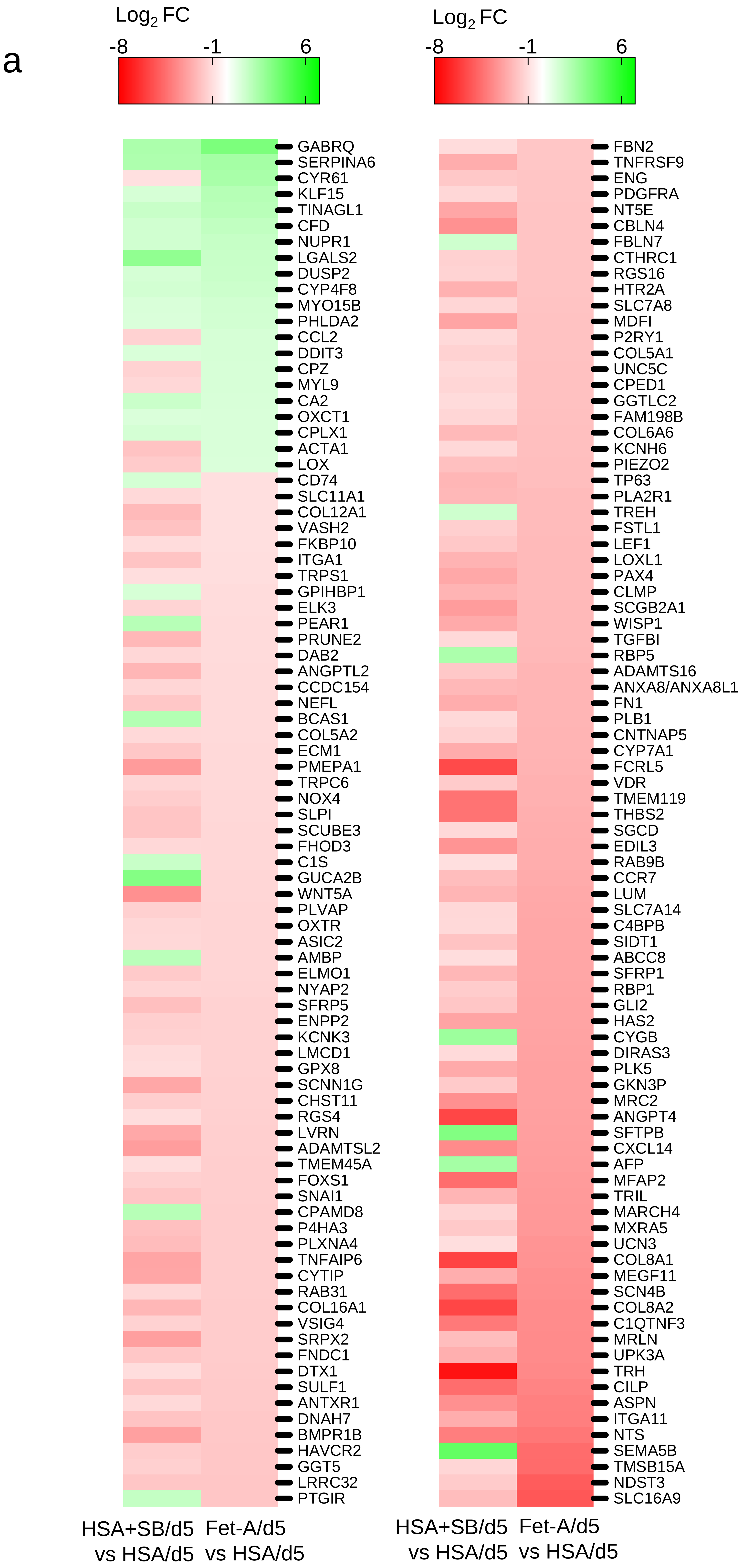


**ESM Figure 2 Fetuin-A and SB431542 inhibit TGFBR-SMAD2/3 signalling and NICCs maturation.** (a) RNAseq-based heat map showing common genes altered (1> $Log_2FC$ < -1) by fetuin-A and SB431542 in NICCs maturated for 5d in medium containing fetuin-A (Fet-A/d5 vs HSA/d5) or HSA + SB (SB/d5 vs HSA/d5)

b

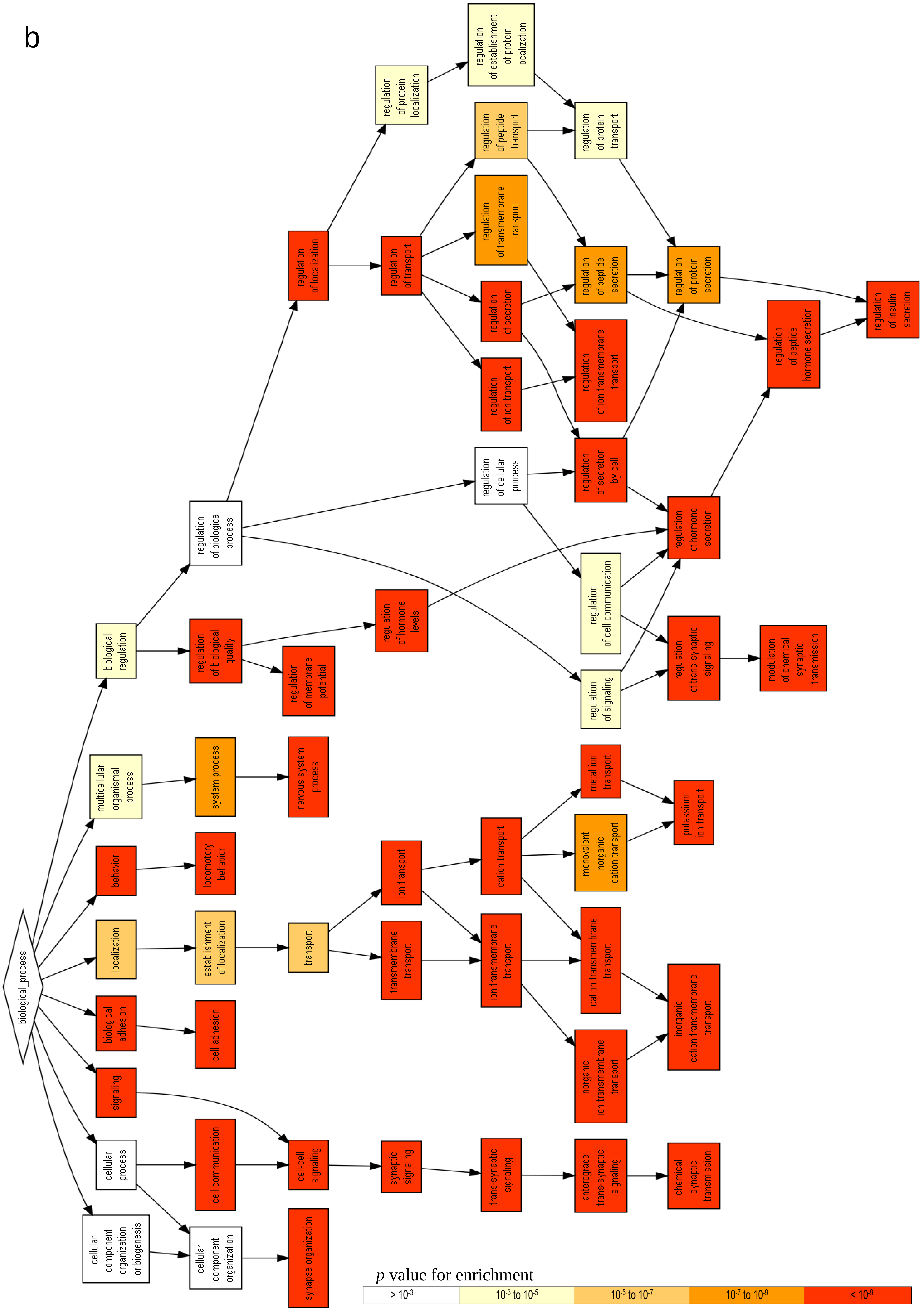


**ESM Figure 2 Fetuin-A and SB431542 inhibit TGFBR-SMAD2/3 signalling and NICCs maturation.** (b) Ranking of GO terms significantly enriched in DEGs upregulated in NICCs upon maturation in standard medium. False Discovery Rate < 2.

c

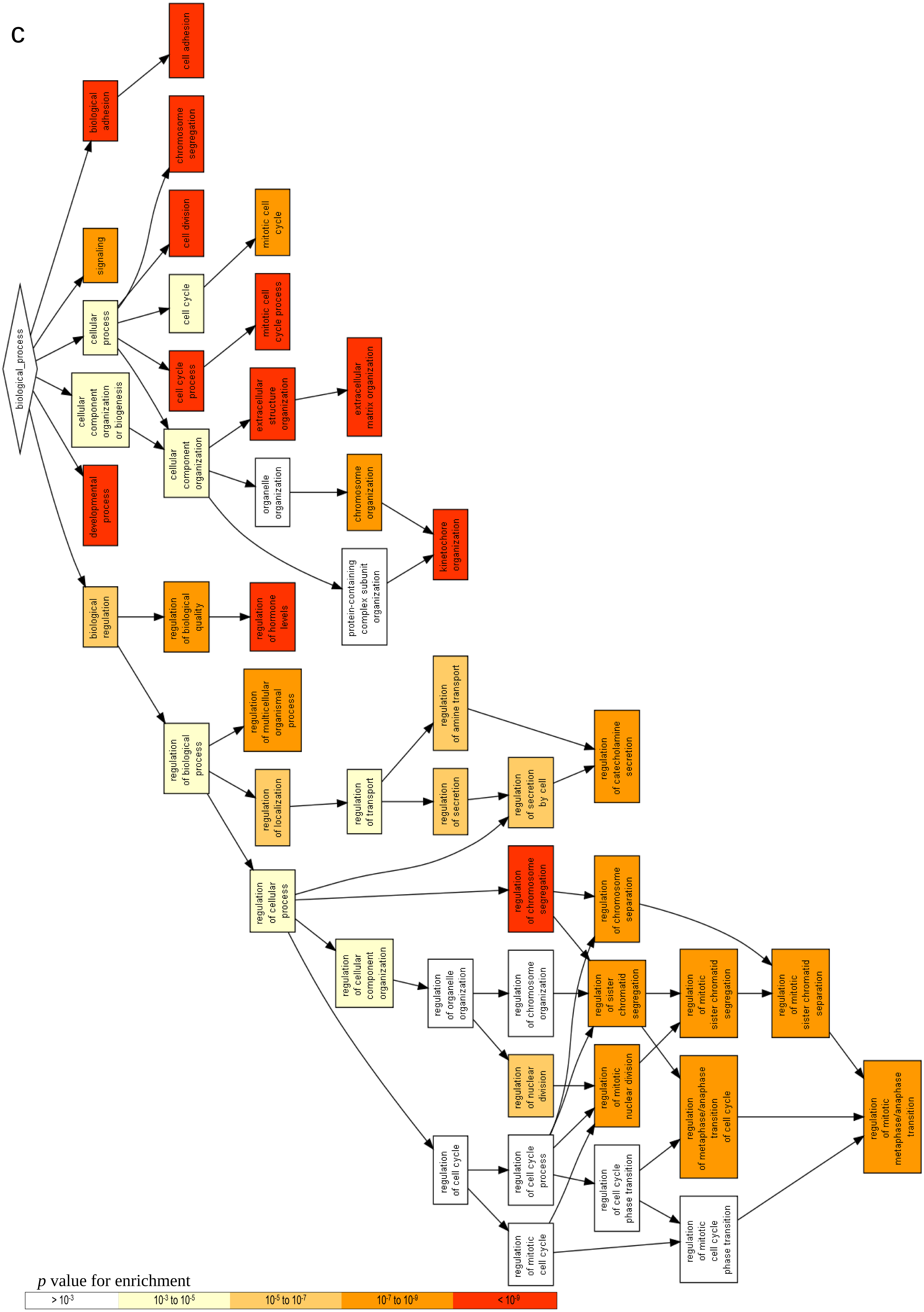


**ESM Figure 2 Fetuin-A and SB431542 inhibit TGFBR-SMAD2/3 signalling and NICCs maturation.** (c) Ranking of GO terms significantly enriched in DEGs downregulated in NICCs cultured in fetuin-A containing medium. False Discovery Rate < 2.

d

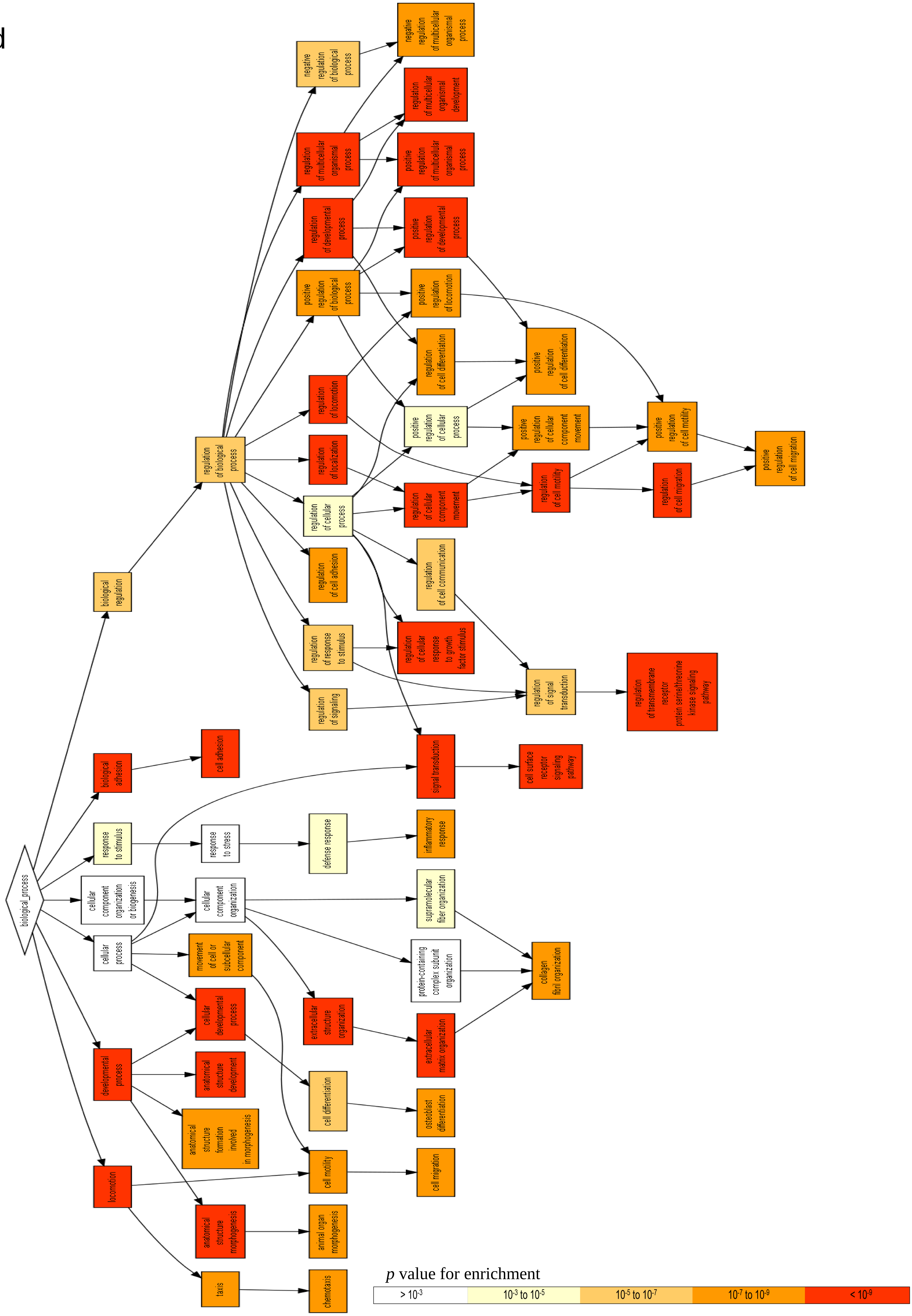


**ESM Figure 2 Fetuin-A and SB431542 inhibit TGFBR-SMAD2/3 signalling and NICCs maturation.** (d) Ranking of GO terms significantly enriched in DEGs downregulated in NICCs upon culture in HSA+SB-containing medium; False Discovery Rate < 2.

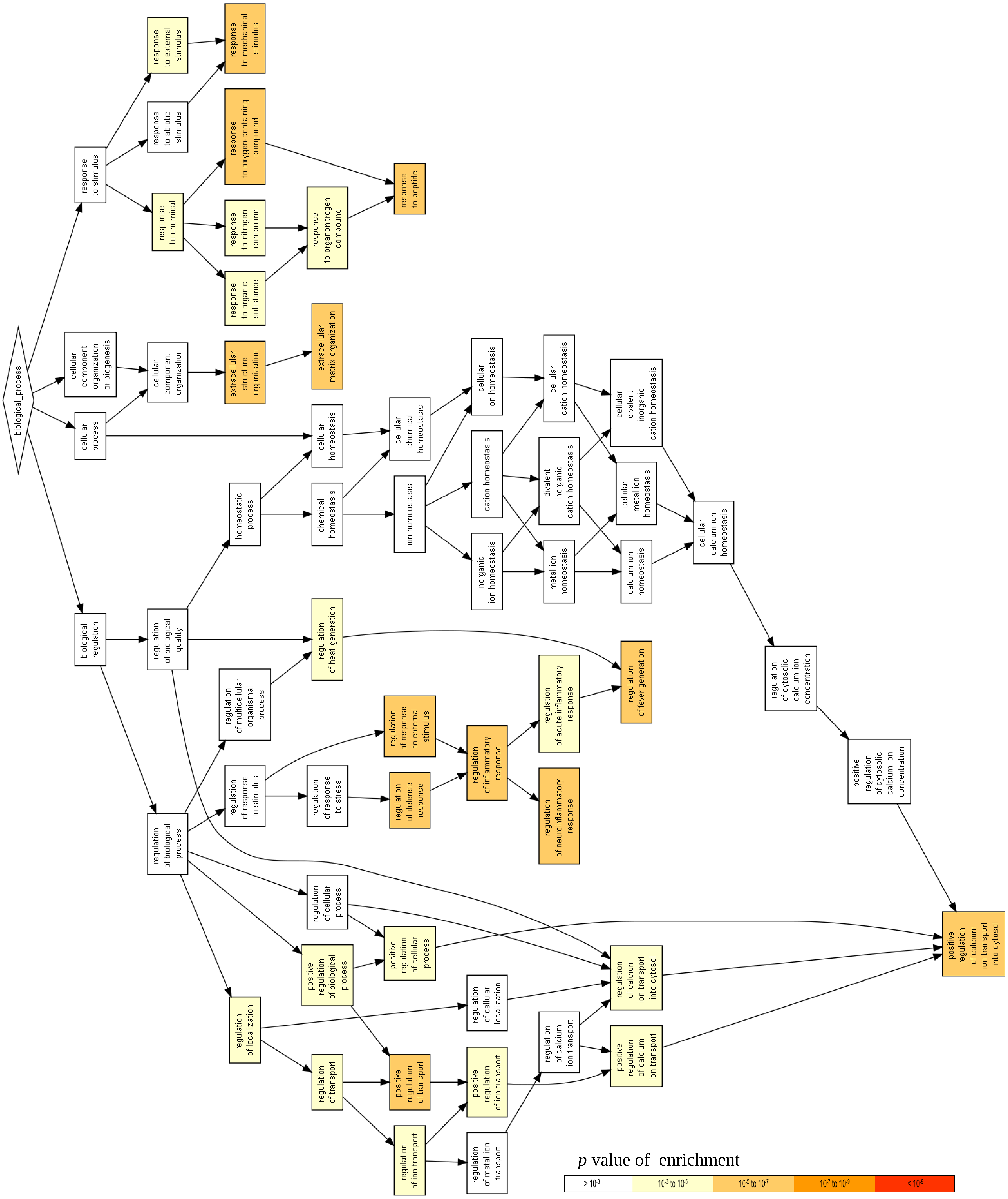


**ESM Figure 3 Fetuin-A inhibits funtional maturation of adult human islets.** Ranking of GO terms significantly enriched in genes altered in human islets following 2d culture in the presence of fetuin-A. False Discovery Rate < 2.

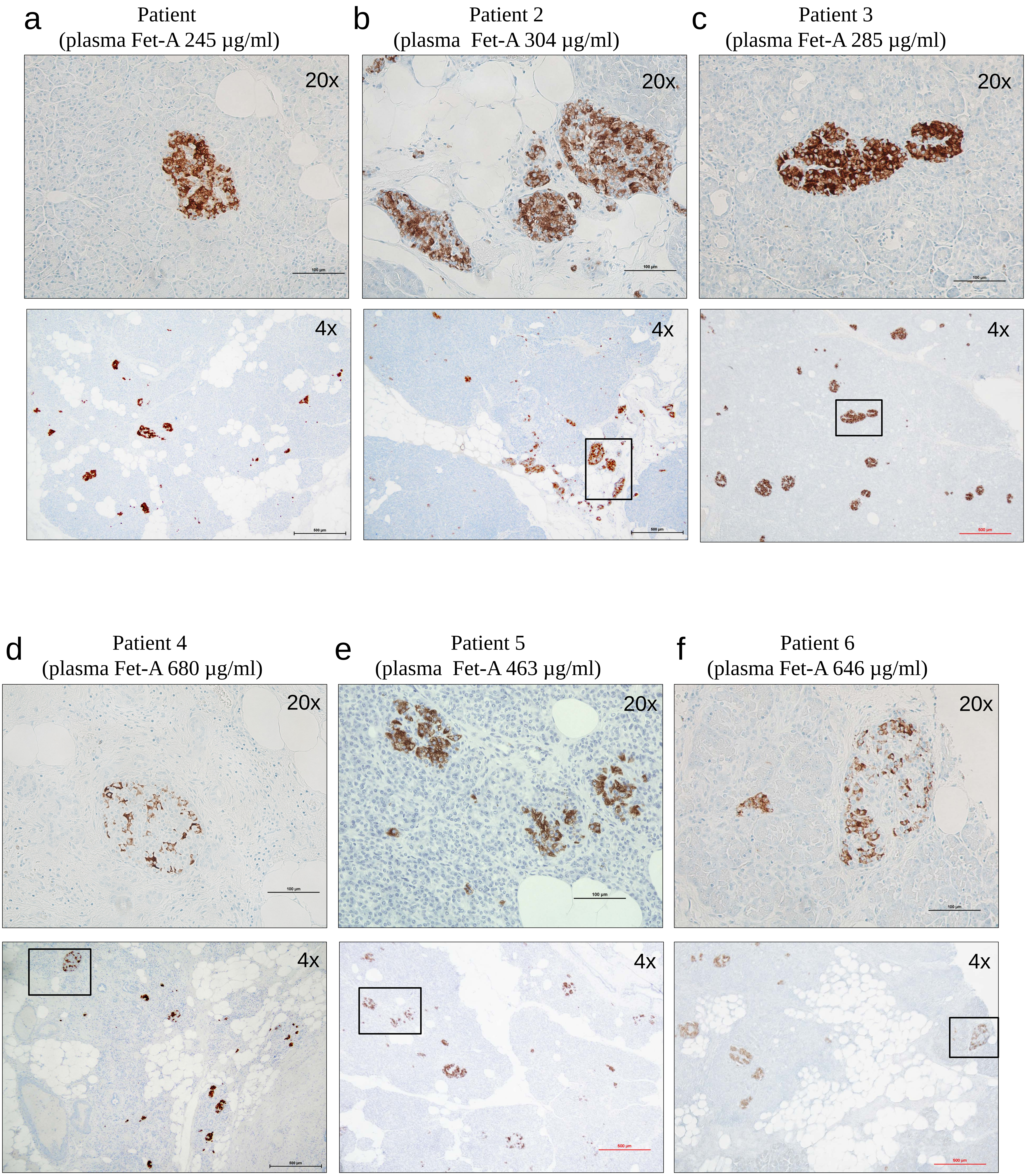


**ESM Figure 4 Human pancreatic islet morphology and elevated fetuin-A levels.** Representative microscopy images of human pancreatic sections from (a-c) n = 3 patients with plasma fetuin-A levels < 400 µg/ml and (d-f) n = 3 patients with plasma fetuin-A levels > 400 µg/ml; the pancreatic sections were stained for insulin (brown) and hematoxylin was used as counter-staining. Upper panels: 20x and lower panels: 4x magnifications.
